# Transcriptional activity of transposable elements in maize

**DOI:** 10.1186/1471-2164-11-601

**Published:** 2010-10-25

**Authors:** Carlos M Vicient

**Affiliations:** 1 Departament of Molecular Genetics, Centre for Research in Agricultural Genomics, CSIC (IRTA-UAB), Jordi Girona, 18, 08034 Barcelona, Spain

## Abstract

**Background:**

Mobile genetic elements represent a high proportion of the Eukaryote genomes. In maize, 85% of genome is composed by transposable elements of several families. First step in transposable element life cycle is the synthesis of an RNA, but few is known about the regulation of transcription for most of the maize transposable element families. Maize is the plant from which more ESTs have been sequenced (more than two million) and the third species in total only after human and mice. This allowed us to analyze the transcriptional activity of the maize transposable elements based on EST databases.

**Results:**

We have investigated the transcriptional activity of 56 families of transposable elements in different maize organs based on the systematic search of more than two million expressed sequence tags. At least 1.5% maize ESTs show sequence similarity with transposable elements. According to these data, the patterns of expression of each transposable element family is variable, even within the same class of elements. In general, transcriptional activity of the *gypsy*-like retrotransposons is higher compared to other classes. Transcriptional activity of several transposable elements is specially high in shoot apical meristem and sperm cells. Sequence comparisons between genomic and transcribed sequences suggest that only a few copies are transcriptionally active.

**Conclusions:**

The use of powerful high-throughput sequencing methodologies allowed us to elucidate the extent and character of repetitive element transcription in maize cells. The finding that some families of transposable elements have a considerable transcriptional activity in some tissues suggests that, either transposition is more frequent than previously expected, or cells can control transposition at a post-transcriptional level.

## Background

Transposable elements (TEs) are DNA sequences that move from one location to another within the genome or can produce copies of themselves. Eukaryotic TEs are divided into two classes, according to whether their transposition intermediate is RNA (class I) or DNA (class II). Each class contain elements that encode functional products required for transposition (autonomous) and elements that only retain the *cis *sequences necessary for recognition by the transposition machinery (non-autonomous). Class I elements can be divided into several subclasses: SINEs, LINEs, long terminal repeat (LTR) retrotransposons and TRIMs (Terminal-repeat Retrotransposons In Miniature), which are LTR non-autonomous elements [1]. Class II elements comprise autonomous and non-autonomous transposons, including the MITEs (Miniature Inverted-repeat Transposable Elements) [2].

TEs are major components of most eukaryotic genomes and are particularly abundant in plants. TEs represent 80% of the maize and 90% of wheat genomes [3]. All the classes of TEs found in Eukaryotes are also present in plant genomes, but LTR retrotransposons are the most abundant in terms of copy number and percentage of genome [4]. 95% of maize TEs are LTR retrotransposons [5].

TEs play an important role in genome and gene evolution. TE insertion can disrupt genes and mediate chromosome rearrangements and can provide alternative promoters, exons, terminators and splice junctions [6]. Several rice genes contain TE derived sequences [7]. However, TE influence in gene expression is not restricted to physical modification of chromosomes. TEs were first characterized in maize as gene ''controlling elements'' [8]. Maize "controlling elements" change the expression of some genes due to the transcription of non-coding RNAs (ncRNA) from the transposon promoters which contribute to the epigenetic regulation of neighbouring genes through mechanisms such as RNAi, transcriptional interference and anti-silencing [9]. The methylation of a SINE element close to the FWA gene, a gene submitted to imprinting, allows the proper epigenetic control in *Arabidopsis thaliana *[10]. TEs also produce short double-stranded RNAs (dsRNAs) which contribute to the epigenetic gene regulation. Analyses in maize, tobacco, wheat or rice have shown that transcriptional readout from retrotransposon LTRs may generate sense and antisense transcripts of adjacent genes, altering their expression [11]. Given the large number of retrotransposon copies in plant genomes and their frequent location near genes, it becomes clear the high potential impact of the TE transcription on the expression of the nearby genes [12,13]. For this reason, TE transcription was believed to be severely repressed in plants. This point of view was supported by the fact that during long time transcription activity was only demonstrated for a few plant TEs, and only activated under certain precise circumstances as, for example, pathogen infection, physical injuries or different abiotic stresses [14,15]. Inactivity of TEs may be due to the accumulation of mutations that have altered their structure. However, although transpositionally inactive due to insertions, deletions, rearrangements or mutations, some copies of the TEs may retain the capacity to direct transcription from their own promoters. In addition to the direct inactivation, cells have also developed mechanism for TE control including silencing by DNA methylation or the small RNA pathways [16]. TEs producing double-stranded or aberrant RNAs are silenced by a post-transcriptional gene silencing mechanism (PTGS) and active TEs are inactivated by transcriptional gene silencing (TGS) [17].

Despite mutations and cell control, TEs manage to be transcriptionally and transpositionally active. Phylogenetic analysis of TE families in maize revealed recent events of extreme TE proliferation [18] and recent transposition activity has also been demonstrated in rice [19]. The use of sensitive techniques for gene expression analysis like deep transcriptome sequencing provide increasing data on the presence of TE-transcripts in several plants and cell types [20-23].

Maize is the plant species from which more ESTs have been sequenced and the third species only after human and mice [24]. More than two million maize ESTs have been sequenced from many libraries corresponding to several maize organs, developmental stages and conditions. It provides a strong basis for the development of computer-based procedures for the *in silico *analysis of expression profiles. The present work aimed to produce a body map of TE transcription in maize plants. We show that the fraction of TE-related transcripts varies greatly among TE classes and among organs.

## Results

### Maize TEs are widely represented in EST databases

The number of ESTs in a large transcript database can be used to estimate relative transcriptional rates. More than two million maize EST sequences are deposited in the NCBI EST database (Zm-dbEST). Such a large amount of sequences provides an opportunity to perform virtual analysis of gene expression in this species. We used a representative sequence of 56 well-characterized TEs (available in the repeats and retrotransposon databases) to query BLASTN against the maize EST database (Zm-dbESTs). 1,5% of the total maize ESTs (25.282 sequences) showed significant sequence homology (e-value < 1E^-20^) with one of the 56 analysed TE families (Additional file 1).

**Figure 1:**
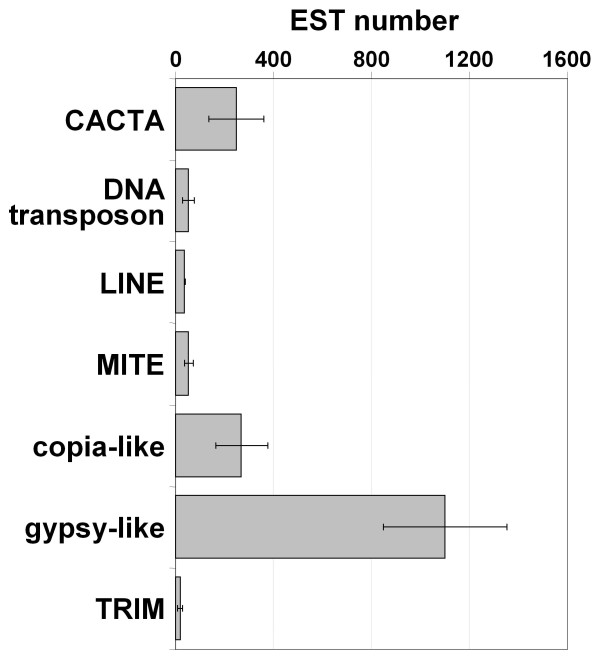
**Transcription levels of the different TE classes**. Number of ESTs in maize databases showing similarity to TEs of different classes. Bars are the average number of ESTs showing similarity to the different members of the indicated TE class and the error bars are the standard errors.

Considering the different TE classes separately, the average number of TE-ESTs obtained for gypsy-like LTR retrotransposons (gypsy) is more than three folds higher than for any other TE class, followed by copia-like LTR retrotransposons (copia) and CACTA (Figure 1). The values within each TE class have a large variability. For example, among gypsy elements, Prem has 4310 ESTs and jaws only two, and among copia elements Opie has 2296 and Hopscotch only one. However, the predominance of gypsy elements is not a result of high presence in EST databases of one or a few families, but generally most of gypsy families have a higher level of transcription compared with other elements. The comparison of the number of TE-ESTs of the families of copia and gypsy elements show a clear overall greater presence of gypsy families in databases (Table 1). Thus, while seven gypsy families have over a thousand TE-ESTs, only *Opie *exceeds this value among copia elements.

**Table 1:** TE families analysed in this study

Class	Family	% genome	Copy number	Total Size	LTR size	ESTs
**CACTA**	En/Spm	n.a.	n.a.	8270	-	46
	Misfit	n.a.	n.a.	10630	-	430
	Yote	n.a.	n.a.	12259	-	267

**DNA Transposon**	Ac/Ds	n.a.	n.a.	4565	-	28
	Doppia	n.a.	n.a.	8463	-	218
	Jittery	n.a.	n.a.	3916	-	6
	Mutator	n.a.	n.a.	4998	-	30
	Mx	n.a.	n.a.	3731	-	3
	PIF	n.a.	n.a.	3712	-	71
	rDT	n.a.	n.a.	704	-	12
	Shooter	n.a.	n.a.	5060	-	54

**LINE**	Cin4	n.a.	n.a.	6822	-	34
	Colonist1	n.a.	n.a.	1658	-	37
	Colonist2	n.a.	n.a.	2575	-	42

**MITE**	Frequent flyer	n.a.	n.a.	526	-	119
	Heart breaker	n.a.	n.a.	315	-	55
	Heart healer	n.a.	n.a.	185	-	122
	mPIF	n.a.	n.a.	343	-	55
	Pangrangja	n.a.	n.a.	607	-	18
	Stowaway	n.a.	n.a.	178	-	2
	Tourist	n.a.	n.a.	150	-	4

**LTR retrotransposon (Copia)**	Bs1	n.a.	n.a.	3.203	302	15
	Eninu	n.a.	n.a.	7.219	1.386	102
	Fourf	0.69	293	6.888	1.210	133
	Giepum	0.55	233	11.756	1.689	559
	Hopscotch	n.a.	n.a.	4.884	227	1
	Ji	18	7650	12085	1.481	209
	Machiavelli	0.48	204	6.153	857	81
	Milt	2	850	9.344	729	424
	Opie	16	6800	8880	1.256	1488
	Raider	n.a.	n.a.	5.865	465	34
	Rire1	0.48	204	7.815	1.270	176
	Ruda	1.64	697	6.431	1.415	507
	Stonor	0.07	29	4.542	560	42
	Victim	0.14	59	5.427	101	6

**LTR retrotransposon (gypsy)**	CentA	n.a.	n.a.	4.634	1.303	147
	Cinful	5	2125	9.568	653	2181
	CRM	n.a.	n.a.	7.571	930	1078
	Dagaf	1.17	497	10.197	3.579	612
	Danelle	4	1700	15.397	4.602	2601
	Diguus	0.69	293	11.679	4.366	634
	Flip	0.62	263	13.555	5.928	704
	Grande	3	1275	13.769	631	365
	Gyma	3	1275	12.246	3.850	520
	Huck	15	6375	11312	1.800	322
	Jaws	0.14	59	6.074	384	1
	Kake	0.07	29	5.914	173	39
	Prem	5	2125	9.469	1.314	3806
	Prem1	1.37	582	10.397	3.383	2198
	Shadowspawn	2	850	11.526	469	399
	Tekay	1.17	497	12.786	3.396	395
	Xilon	4	1700	11.514	2.467	1865
	Zeon	8	3400	7.459	698	1930

**TRIM**	Cassandra/TIM-1	1.10	467	734	260	18
	Magellan	n.a.	n.a.	1313	-	1
	ZmTRIM	n.a.	n.a.	809	-	36

**Figure 2:**
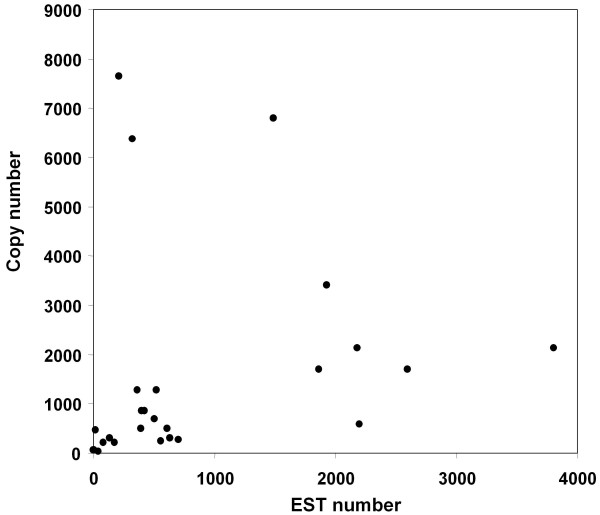
**Dot-plot correlation between TE copy number and number of EST similar to the TE**. Correlation between the TE copy number and the number of ESTs in databases showing sequence similarity to a TE family.

Previous works suggest that a negative correlation exists between retrotransposon copy numbers and transcription levels [25]. We compared the copy numbers of each TE family with the numbers of ESTs and we did not find correlation (positive or negative) (r = 0.209) (Figure 2). There are TE families with low copy numbers and several ESTs (Flip has 263 copies and 704 ESTs), and TE families with high copy numbers and few ESTs (Ji has 7.650 copies and only 209 ESTs). The lack of correlation between copy and EST numbers is also an indication that no extensive genomic contamination is present in the maize dbEST. We also checked for possible correlations between copy number and EST numbers looking for each of the libraries individually and we found no significant correlations in any of them (data not shown).

The distribution of the EST matches along the TE sequence was examined (Additional file 2). The EST distribution was variable depending on the TE element family, but a general similar behaviour was observed within classes. For example, in LINEs, most of the ESTs showed similarity with the 3'end. On the other hand, in LTR retrotransposons and TRIMs, most of the ESTs are similar to the LTR regions. These non-random distribution is probably a consequence of the different transcription mechanism characteristic of each TE family.

**Figure 3:**
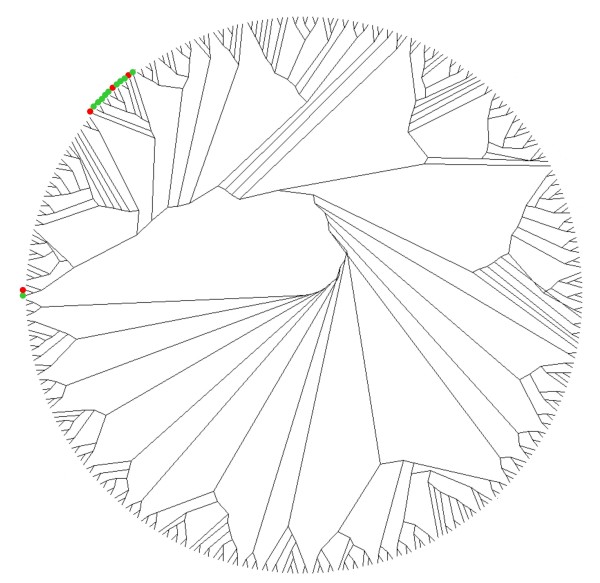
**Phylogenetic analysis of retrotransposon Grande TE-ESTs and genomic sequences**. Phylogenetic analyses were performed using the neighbor-joining algorithm from distance matrices according to Kimura's two-parameter method. Randomly selected genomic sequences are indicated by unlabeled nodes. ESTs are indicated by circles. The colour of the circle indicates the organ from which the cDNA library was constructed: red, male flower; green, shoot apical meristem.

TE families are comprised by hundreds or thousands of copies inserted in the genome. A question arises of how many of these copies are transcriptionally active. Are the TE transcripts produced by several copies or only by one or a few of them? In order to answer this question we aligned randomly selected genomic sequences and transcribed sequences of some of the TE families and we constructed phylogenetic trees (an example is in Figure 3 and more data are available in Additional file 3). Several clades of genomic sequences were detected, some of them composed by many closely related copies which probably represent recent transposition bursts. EST sequences are located only in a few of these clades, which indicate that only few evolutionary branches of the TEs have retained transcriptional competence. A similar situation was observed in all families analysed (Additional file 3). It does not exist a correlation between the active subfamily and transcription in certain organs. These results also suggest that genomic contamination in the EST databases, if exists, is negligible and that the observed EST frequencies are indicative of transcriptional activity of the TE families in maize.

### Profiles of TE transcription in different maize organs and conditions

**Figure 4:**
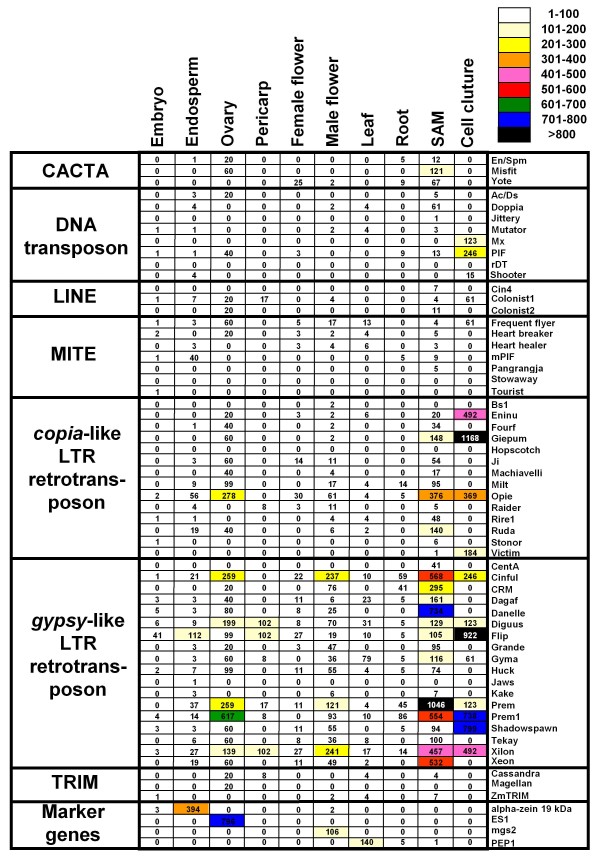
**Patterns of TE-EST presence in different maize organs and conditions**. Lines represent different TE families grouped by classes, and columns different organs and conditions. Numbers are the normalized values of EST abundance. Background colours indicate the TE abundance as indicated by the colours scale. Marker genes are: alpha zein 19 kDa (Acc.Number: J01244), female gametophyte-specific protein ES1 precursor (ES1, Acc.Num. AF180131), male gametophyte-specific 2 (mgs2, Acc.Num. L20139) and phosphoenolpyruvate carboxylase 1 (PEP1, Acc.Num. X15238).

**Figure 5:**
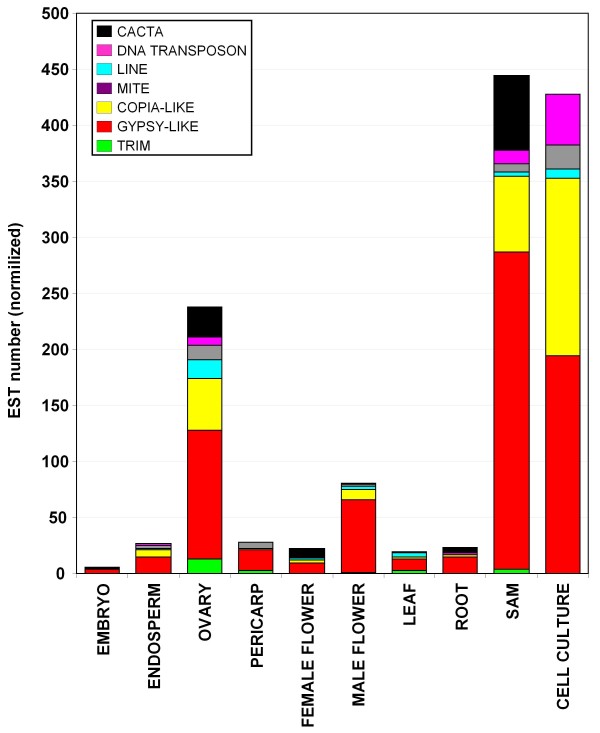
**Presence of TE-ESTs of different TE classes in maize organs and conditions**. Average presence of ESTs homologous to TE in different maize organs and conditions.

Many of the maize ESTs were obtained from libraries constructed with RNA extracted from dissected organs. This allows us to study organ-specific expression of the different TE families using a "virutal northern" strategy based on the abundance of the ESTs on each library. ESTs matching the TEs were divided according to the library they were sequenced from. Due that for some of the libraries the number of ESTs was not enough to guarantee a deep analysis, we grouped the libraries constructed from the same or related organs. Each group of libraries contains a different number of sequences, so, direct comparison between organs is not possible. A normalization process was done dividing the matches by the total number of sequences in each of the groups. The resulting numbers reflect the overall expression level of each family among the different organs and conditions tested. As a result, we obtained expression values for each of the 56 TE families in 12 different organs or conditions (Figure 4). As a control, we used the same analysis for four genes of known and precise patterns of expression: *zein*, *es1*, *mgs2 *and *PEP1*. In all cases the analysis gave us the expected patterns of expression for all marker genes, *zein *in endosperm, *es1 *in ovary, *mgs2 *in male flower and *PEP1 *in leaves. The TE expression patterns vary considerably between and within classes. Whereas many TE families show low levels of expression in all organs and conditions, others have especially high levels in certain organs. Several retrotransposons seem to be specially expressed in apical meristem like Prem or Danelle. Giepum, Flip, Prem1 and Shadowspawn are specially expressed in cultured cells and Prem1 is also highly represented in ovary. Considering all the TE classes, shoot apical meristem and cell culture are clearly the organs with a higher presence of TE-ESTs, followed by ovary and male flower (Figure 5). As we showed previously (Figure 1), gypsy elements are the most abundantly represented in the EST databases, and this is also true if we consider separately the different organs. CACTA are specially represented in SAM and ovary, DNA transposons in cultured cells, LINEs in ovary, and TRIMs in ovary.

**Figure 6:**
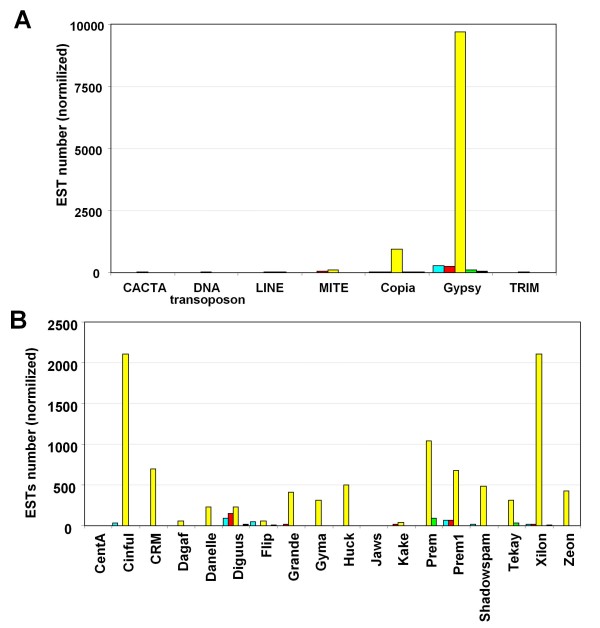
**Presence of ESTs similar to TEs in maize male flower libraries**. A) Presence of TEs of different classes in EST collections obtained from different parts of maize male flowers. Bars indicate the average normalized values for each class and EST collection. Colours indicate the tissues or cells from which the libraries were constructed: blue, anther; red, pollen; yellow, sperm cell; green, tassel; and black, tassel primordium. B) Presence of different families of gypsy-like LTR-retrotransposon in ESTs collections obtained from different parts of maize male flowers. Bars indicate the average normalized values for each family and EST collection. Colour code as in A.

Relatively high number of TE-ESTs have been observed in male reproductive organs (Figure 5). This is interesting because this organ can potentially lead to the germ line cells, allowing a possible transposition event to be inherited. However, "Male flower" group is composed by sequences obtained from six cDNA libraries obtained from dissected anther, pollen, sperm cell, tassel and tassel primordium. So, we decided to examine in more detail the origin of the TE-ESTs for this category doing the same analysis, but considering separately the different libraries. We observed that the majority of the TE-ESTs were originated in the sperm cell library (Figure 6A). Gypsy-like retrotransposons are specially represented in the male flower category and most of the ESTs were originated from the sperm cell library. Looking at the different gypsy-like families individually, predominance of sperm cell was observed in all of them (Figure 6B).

## Discussion

"Virtual northern" analysis provides an easy and cheap alternative to the study of transcriptional profiling. An advantage of EST profiling compared with other methods is that it does not require prior knowledge of the gene sequences. The accuracy of "virtual northern" analyses will depend on the diversity of biological samples and in the number of sequence tags to provide sufficient depth to identify low-abundant transcripts. An additional problem will be the possibility to distinguish between closely related genes in the basis of partial sequences. EST profiling have been used for the identification of reference genes for quantitative RT-PCR normalization in wheat [26] and barley [27], expression profiling of storage-protein gene families in wheat [28], identification of differentially expressed transcripts from sugarcane maturing stem [29], or the identification of cancer gene-markers in humans [30]. The application of EST profiling to maize TEs is particularly appropriate. First, the analysis of TE families, and not single genes, virtually eliminates the problem of distinguishing between closely related sequences. Second, maize is the third organism in number of ESTs, and, finally, several of the cDNA libraries were constructed from precise, well-defined, dissected organs. The applicability of EST profiling in maize is demonstrated by the expected results for some marker genes (figure 4). One possible problem is the presence of sequences originated from contaminant genomic DNA in the EST collections. This problem is especially serious in the case of TEs because some of them are present in high copy numbers in the genome. Although we cannot totally exclude the presence of some genomic contamination, our results indicate that, if any, it may be considered anecdotic (Figure 2).

Once integrated in the genome, TEs accumulate mutations and become transpositionally inactive. However, even partial or rearranged TE copies may retain their capacity to initiate transcription. Cells have active mechanisms to protect their genome integrity against TE activity including transcriptional silencing [31] and short-interfering RNAs (siRNAs) [32]. Under certain circumstances some TEs can escape this cell control and transcribe and, sometimes, transpose [19]. For example, different TE families are transcribed in response to biotic or abiotic stresses or in cell culture [33-37]. In addition to these "stress response" transcription, increasing data demonstrate that some TEs may have at least low transcriptional activities under normal circumstances in plant life. For example, transcription in leaves has been demonstrated for barley BARE, maize Grande and tomato Rider retrotransposons, and in different sorghum TEs [38-41]. Different EST based analysis, including the data presented here, demonstrate the presence of TE-transcripts in several organs and cell types [20-22,42,43]. According to our data, at least 1.5% of the ESTs correspond to TEs. This is an underestimation because only well characterized maize TEs were considered in our analysis and because ESTs libraries only contain data on polyadenylated mRNAs and it is not clear which percentage of TE transcripts contain a polyA track. For example, it has been estimated that only 15% the transcripts of the barley retrotransposon BARE1 are polyadenylated [44]. In any case, the percentage is different according to the organs analysed ranging from 7.7% in SAM and 6.2% in cultured cells, to only 0.2% in female flowers and 0.1% in embryo.

TE transcripts are specially abundant in SAM, cultured cells (Figure 4; [23]) and sperm cells (Figure 5; [31,45-47]). A common feature of SAM, pollen and cell cultures is that they contain pluripotent cells. Animal totipotent cells like oocytes and two-cell mouse embryos also exhibit high levels of TE transcription [48]. The acquisition of totipotency depends, among other things, on epigenetic reprogramming [49] and activation of TEs has also been associated with reductions on DNA methylation [50]. For example, DNA in plant cultured cells undergoes hypomethylated and these cells show a transcriptional activation of specific TEs [31]. Tobacco Tnt1 retrotransposon is silenced when introduced in Arabidopsis, but reversion of Tnt1 silencing is obtained when the number of Tnt1 elements is reduced to two by genetic segregation [51]. Microarray expression profiling of Arabidopsis mature pollen revealed that many of the genes involved in siRNA biogenesis and silencing are not expressed in pollen or expressed at low levels [52]. Although epigenetic changes may explain activation of certain TEs in some tissues, not all TE families accumulate equally in SAM or sperm cells, suggesting that the phenomenon requires some family specific mechanisms rather than simply being the result of a genome-wide activation of retrotransposons. One possible explanation may be the presence of *cis *specific signals in the TE promoter that may enhance their expression in certain cells. For example, pollen promoter specific signals have been detected in the LTR of Grande (personal unpublished data).

## Conclusions

The use of powerful high-throughput sequencing methodologies allowed us to elucidate the extent and character of repetitive element transcription in maize cells. Next-generation sequencing of transcriptomes and genomes will enable further studies on TE transcription and their consequences.

## Methods

### Data sources and analysis

Model TE sequences were obtained from the TIGR Plant Repeat Databases [53], Retrotransposon Database [54] and GeneBank. Only well characterized elements were included in our analysis (Table 1). Model sequences were compared with the maize (*Zea mays *L.) entries in NCBI dbEST release 092509 [24], which contained 2,018,634 sequences from 315 cDNA libraries, using BLASTN analysis [55]. BLAST analysis were performed using an expected threshold of 10, a word size of 11, a match/mismatch of 2-3 and gap cost existence of 5 and extension of 2. We only considered positive such sequences showing an e-value < 1E^-20^. For organ-specific expression analysis libraries in the UniGene data set constructed from the same (or related) organ(s) were assigned into common pools. Libraries with insufficient information regarding the source organ or constructed from mixed parts of the plant were excluded from the analysis. These efforts resulted in organ groupings each containing different numbers of libraries and ESTs (Table 2). The differences in the clone numbers between organ groups do not allow a direct comparison of EST numbers. A normalization process was done dividing the number of ESTs by the total number of sequences in the organ group. Normalization values were expressed as 1 per 10.000.

**Table 2:** Organ/condition maize EST databases used in this analysis

Organ/conditions	Number of libraries	Number of ESTs
Embryo	14	23.334
Endosperm	15	67.625
Ovary	8	33.604
Pericarp	4	10.440
Female flower	15	49.972
Male flower	12	43.842
Leaves	31	38.133
Root	20	39.810
Shoot apical meristem	15	593.966
Cell culture	6	14.058

Sequence alignments were performed using CLUSTALW and phylogenetic trees using neighbour joining method. Graphic representation of phylogenetic trees were prepared using Dendroscope v.2.7.4 [56].

## List of Abbreviations

TE: transposable element; TE-EST: EST: with sequence similarity to a transposable element; LTR: long terminal repeat; LINE: long interspersed transposable elements; MITE: Miniature Inverted Transposable Element; TRIM: Terminal-repeat Retrotransposons In Miniature.

## Authors' contributions

The author did all the analyses and wrote the manuscript.

## Supplementary Material

Additional file 1 **Maize ESTs displaying sequence homology with TEs**. Click here for file

Additional file 2 **Distribution of homologies of the maize TE-ESTs along the corresponding TE sequences**. Schematic representation of the regions of the different TE families showing similarity to a maize EST. Bar in the top represent the TE element and smaller bars on the bottom the different ESTs. When the number of ESTs was high only the ESTs showing higher similarities were represented. Click here for file

Additional file 3 **Phylogenetic analysis of TE-ESTs and genomic sequences of different transposable elements**. Phylogenetic analyses were performed using the neighbor-joining algorithm from distance matrices according to Kimura's two-parameter method. Randomly selected genomic sequences are indicated by the accession number of the sequence from which were obtained. ESTs are indicated by their accession numbers. The position of the ESTs is also indicated by circles. The colours of the circles indicate the organ from which the cDNA library was constructed. Click here for file
