# Supplementary material for: Transcriptional activity of transposable elements in maize
Source: BMC Genomics. 2010 Oct 25;11:601. doi: 10.1186/1471-2164-11-601 (PMC3091746; doi:10.1186/1471-2164-11-601)
Supplement: Additional file 2 — Distribution of homologies of the maize TE-ESTs along the corresponding TE sequences. Schematic representation of the regions of the different TE families showing similarity to a maize EST. Bar in the top represent the TE element and smaller bars on the bottom the different ESTs. When the number of ESTs was high only the ESTs showing higher similarities were represented. [file 1471-2164-11-601-S2.PPT]

## Slide 1
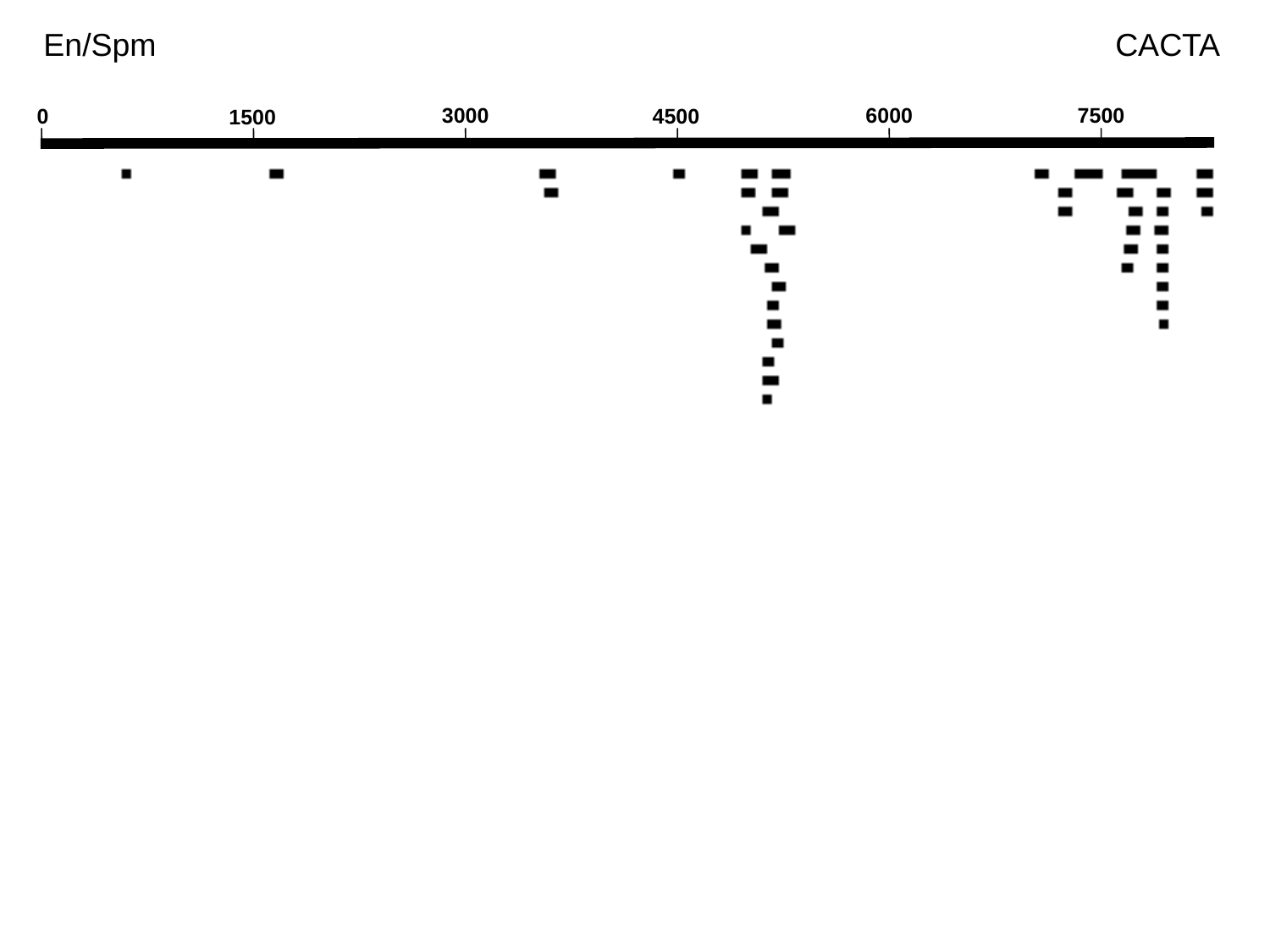

En/Spm
CACTA
7500
3000
6000
4500
0
1500

## Slide 2
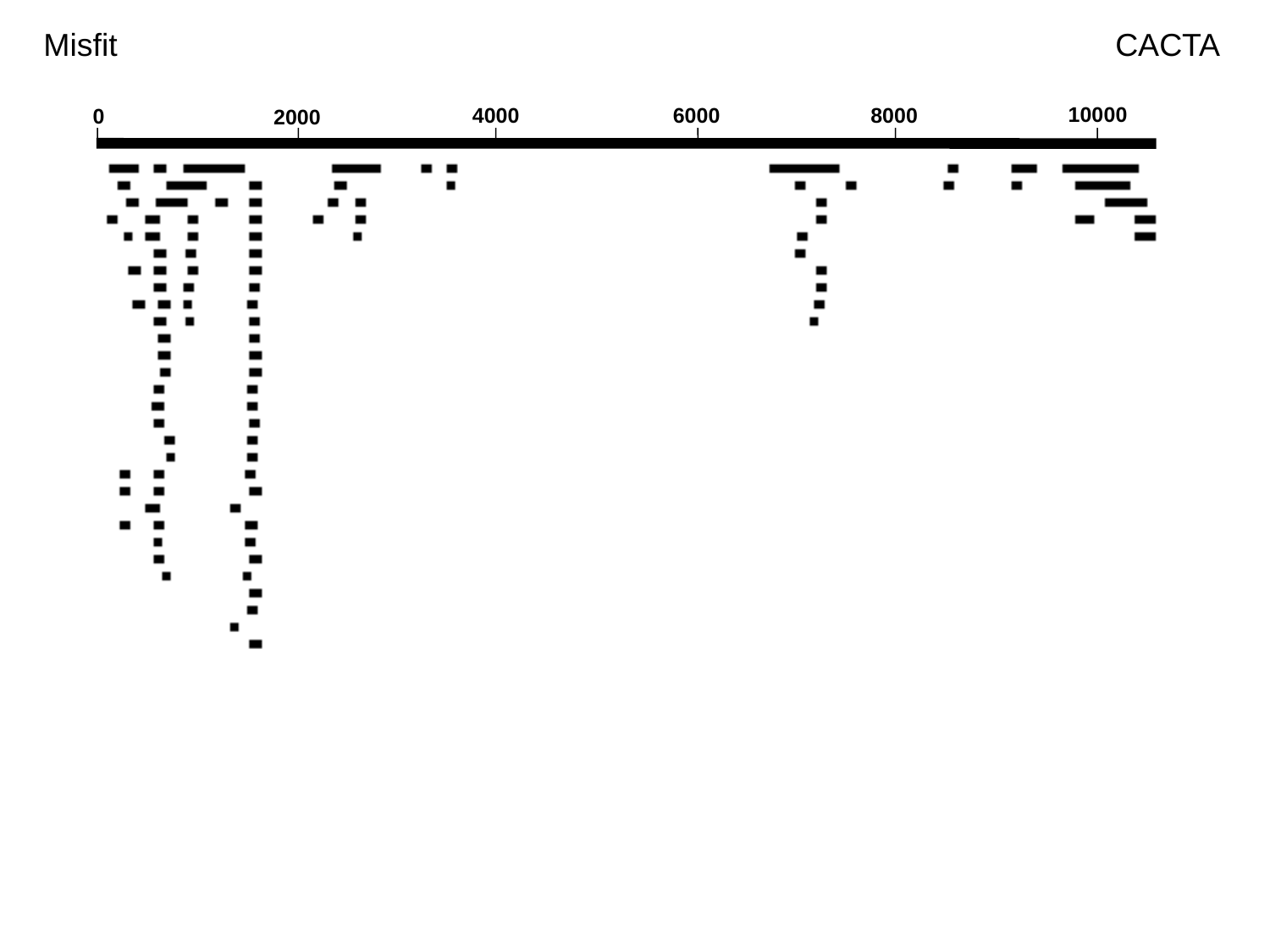

Misfit
CACTA
10000
4000
8000
6000
0
2000

## Slide 3
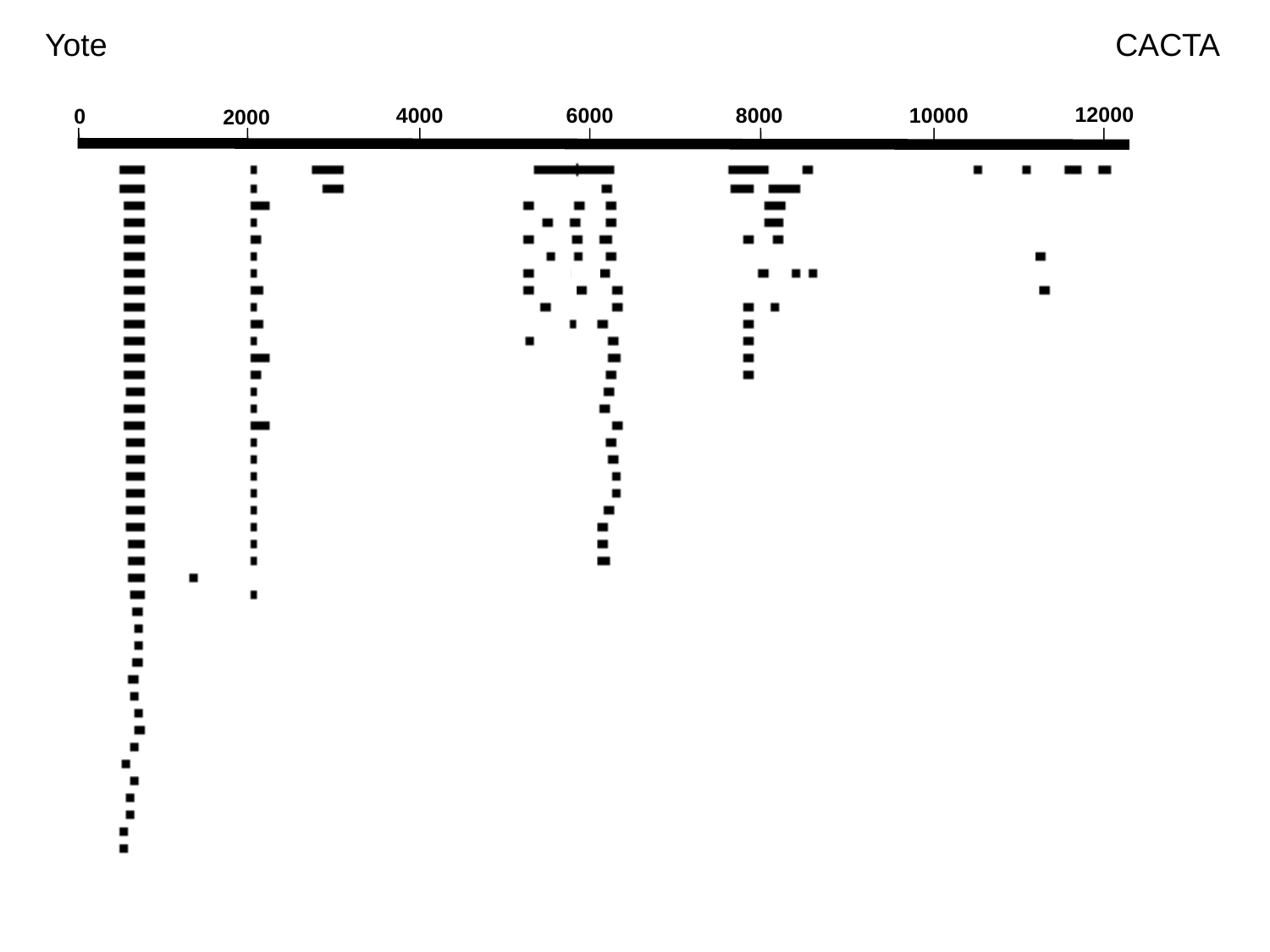

Yote
CACTA
12000
4000
10000
6000
8000
0
2000

## Slide 4
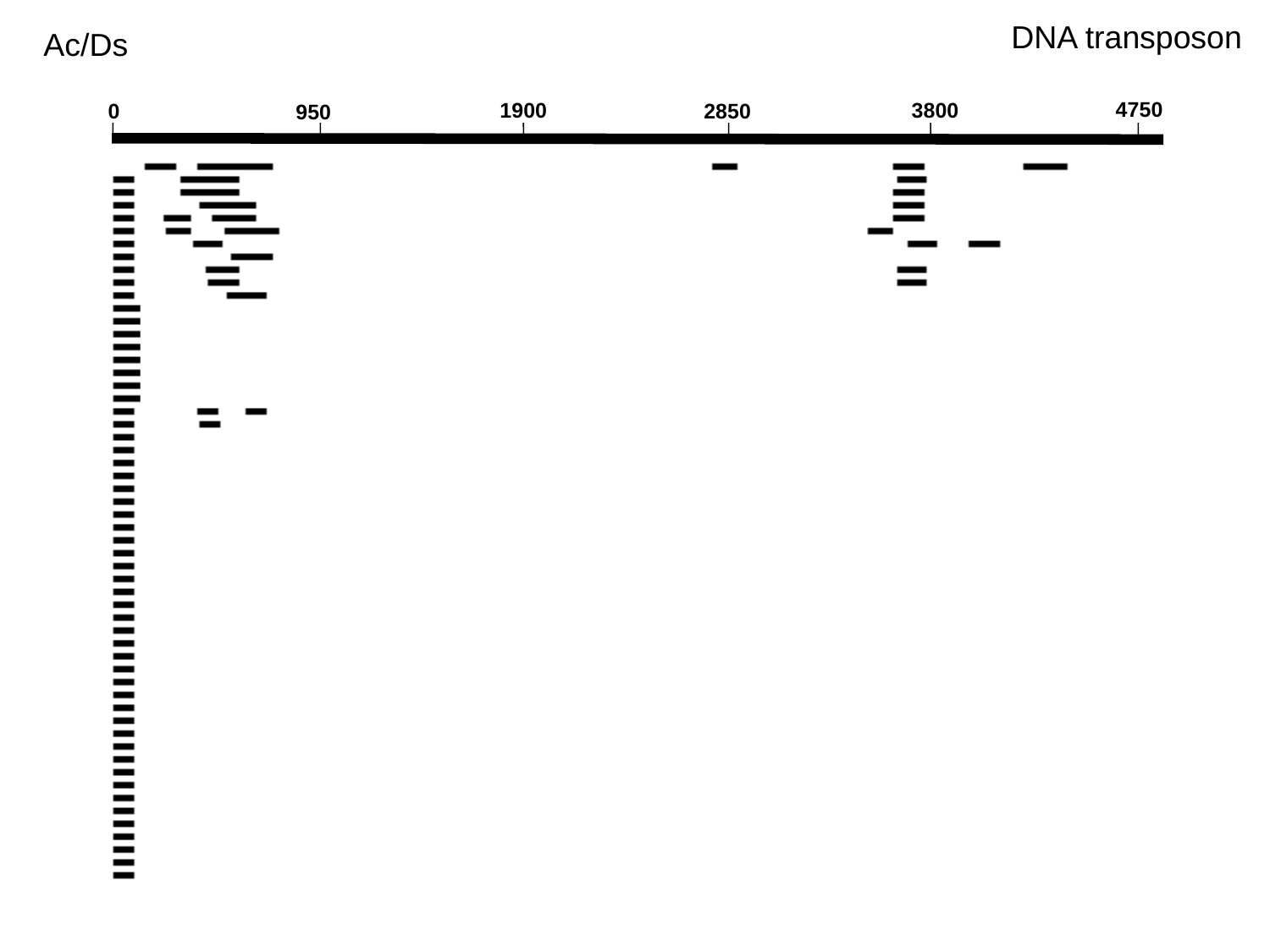

DNA transposon
Ac/Ds
4750
1900
3800
2850
0
950

## Slide 5
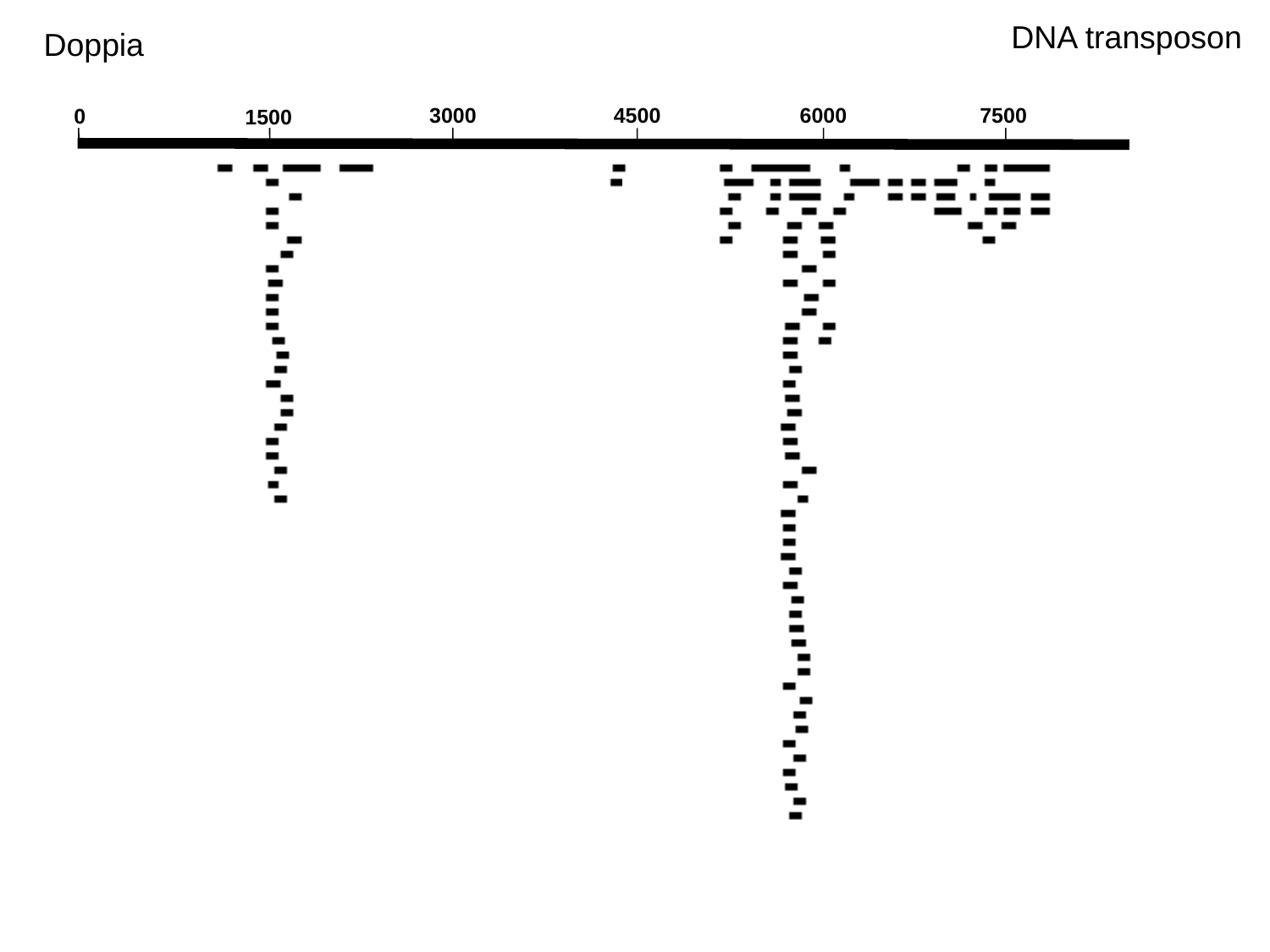

DNA transposon
Doppia
3000
7500
4500
6000
0
1500

## Slide 6
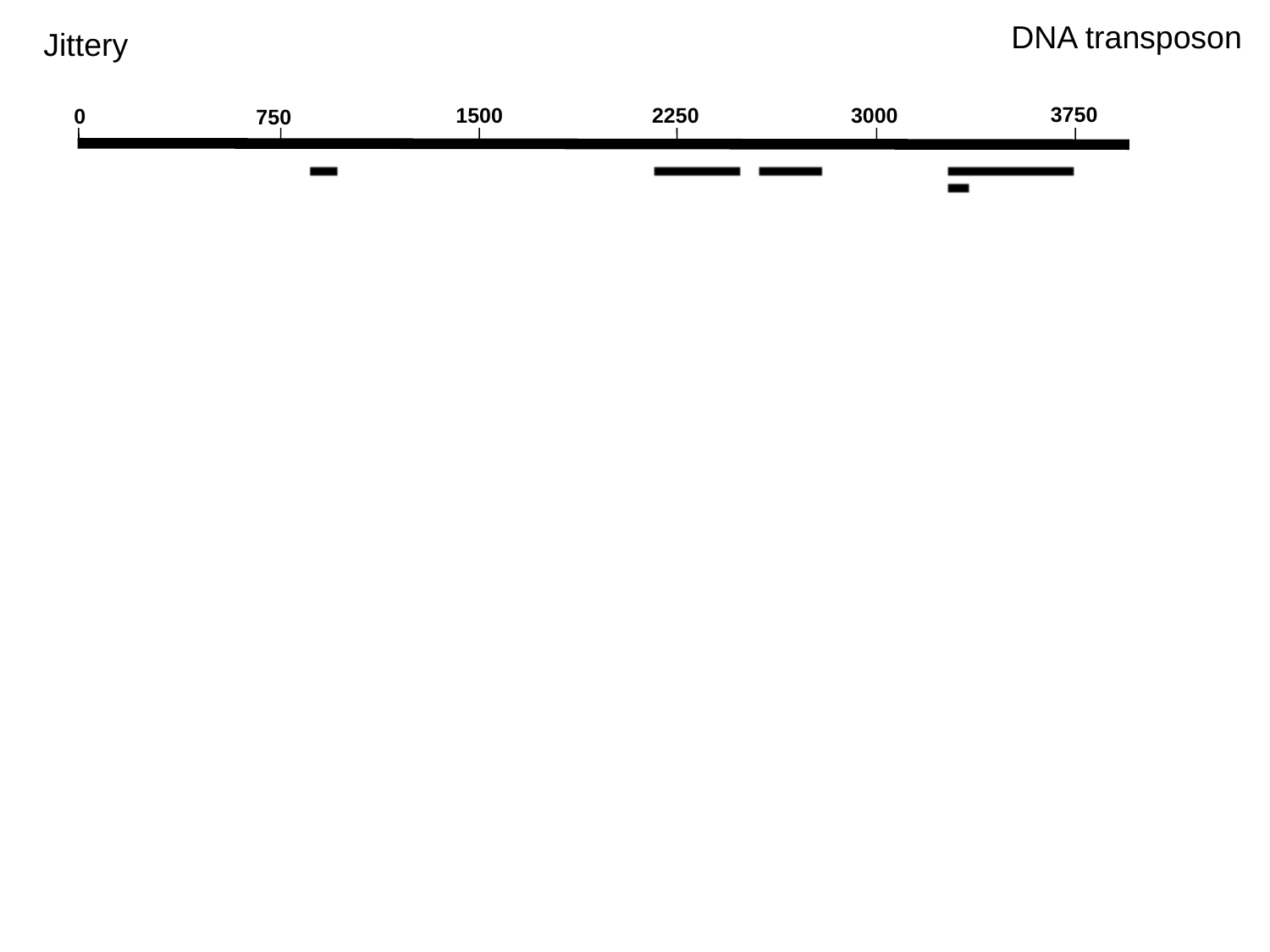

DNA transposon
Jittery
3750
1500
3000
2250
0
750

## Slide 7
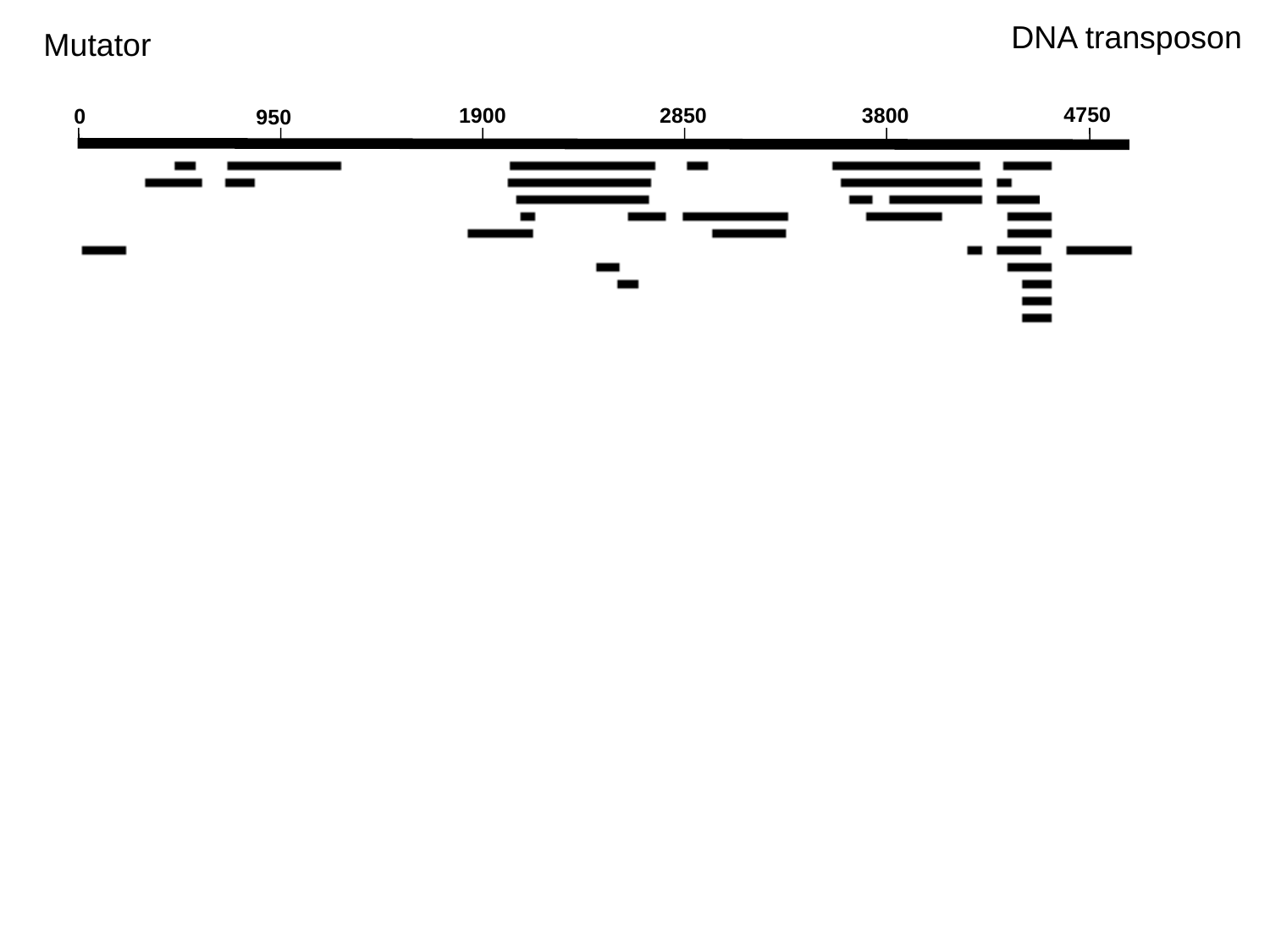

DNA transposon
Mutator
4750
1900
3800
2850
0
950

## Slide 8
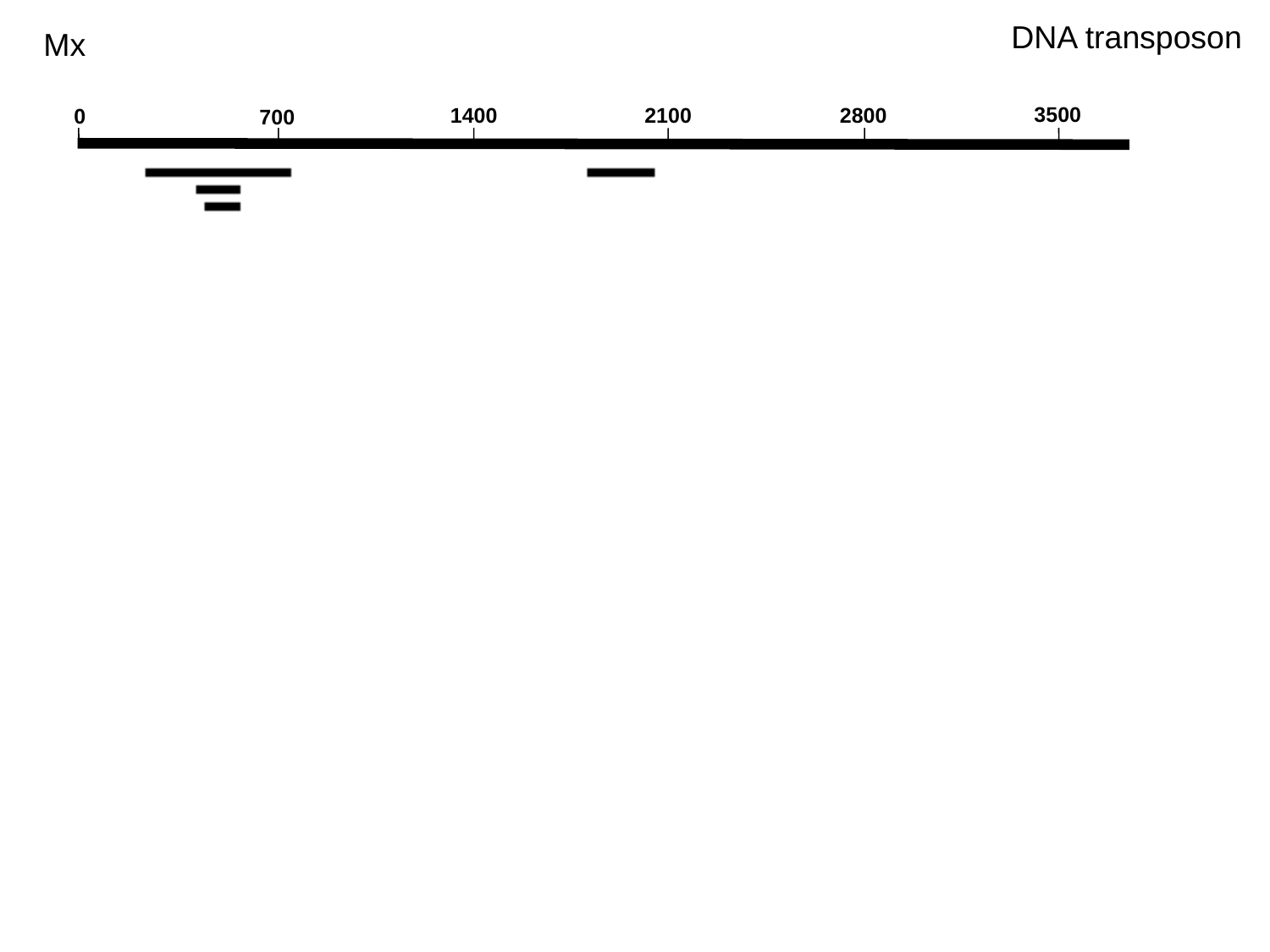

DNA transposon
Mx
3500
1400
2800
2100
0
700

## Slide 9
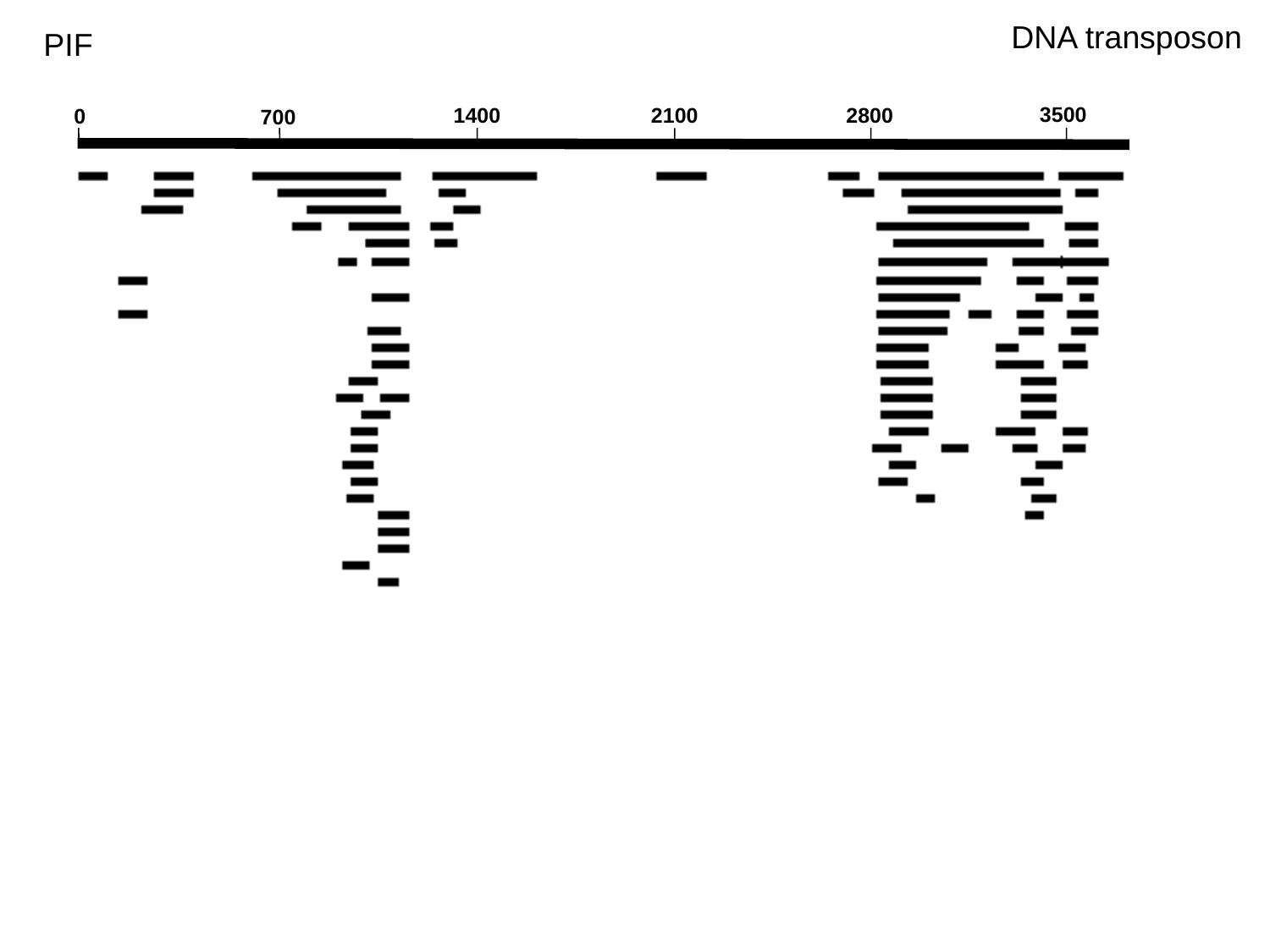

DNA transposon
PIF
3500
1400
2100
2800
0
700

## Slide 10
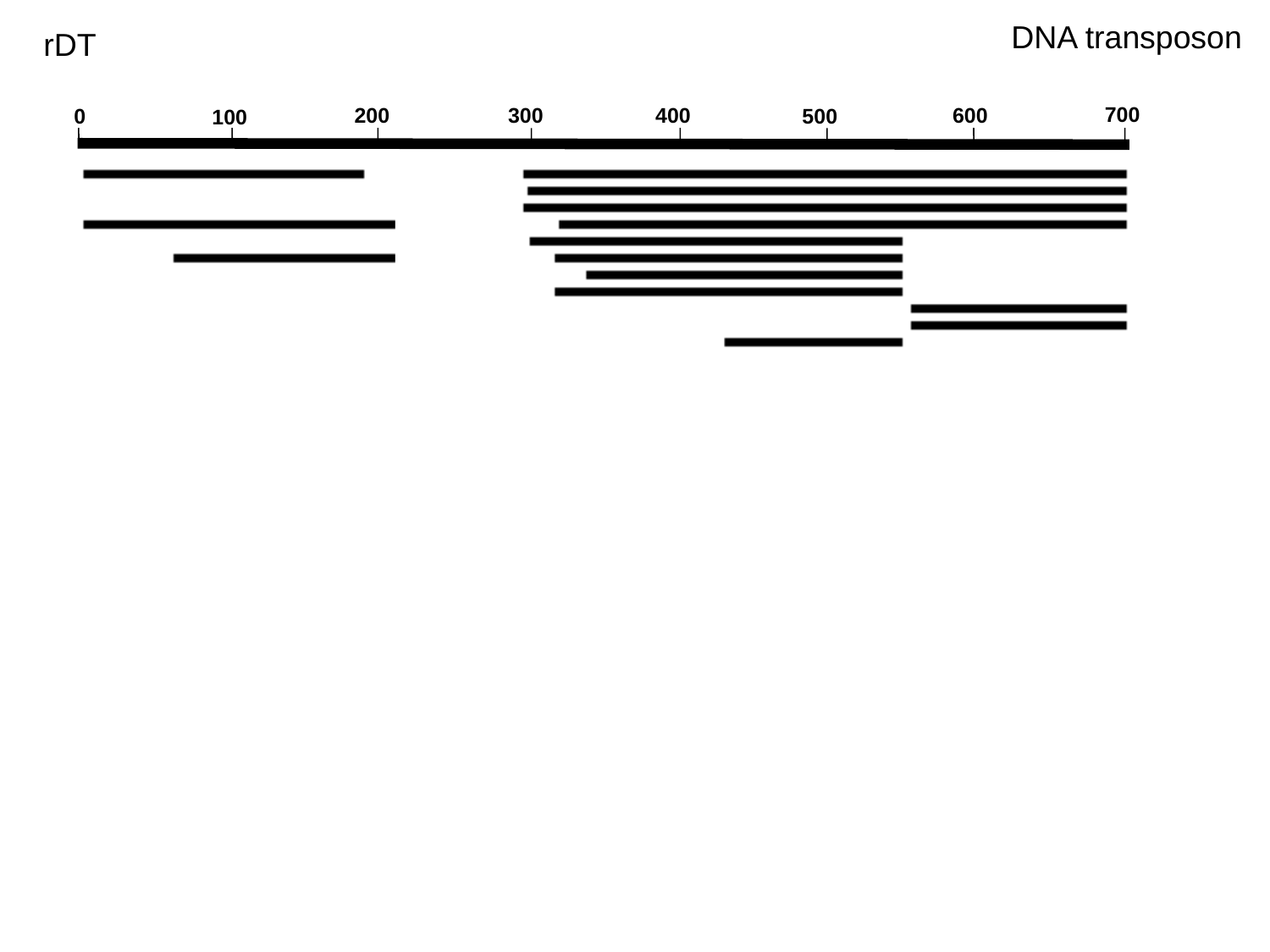

DNA transposon
rDT
700
200
600
300
400
500
0
100

## Slide 11
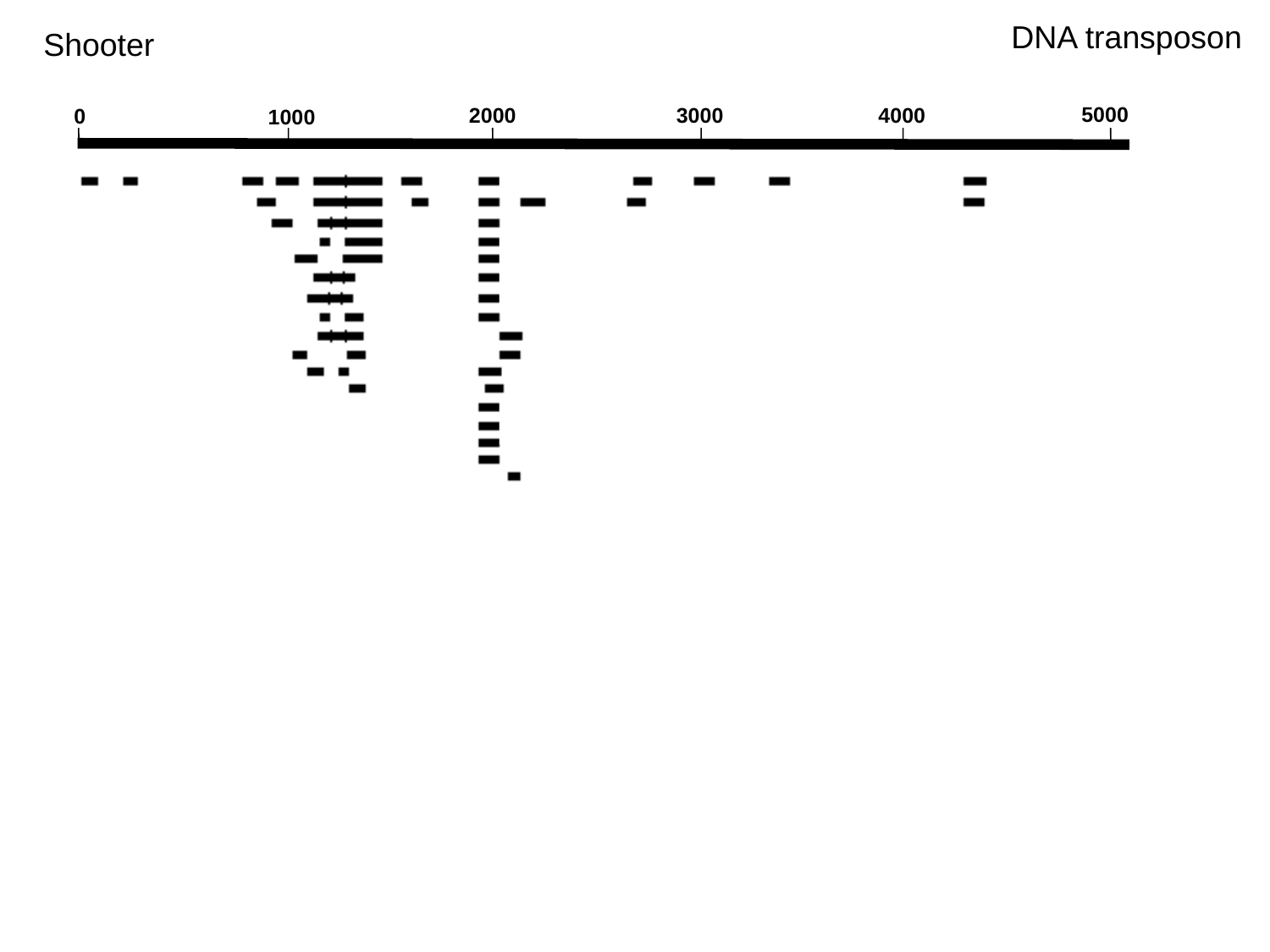

DNA transposon
Shooter
5000
2000
4000
3000
0
1000

## Slide 12
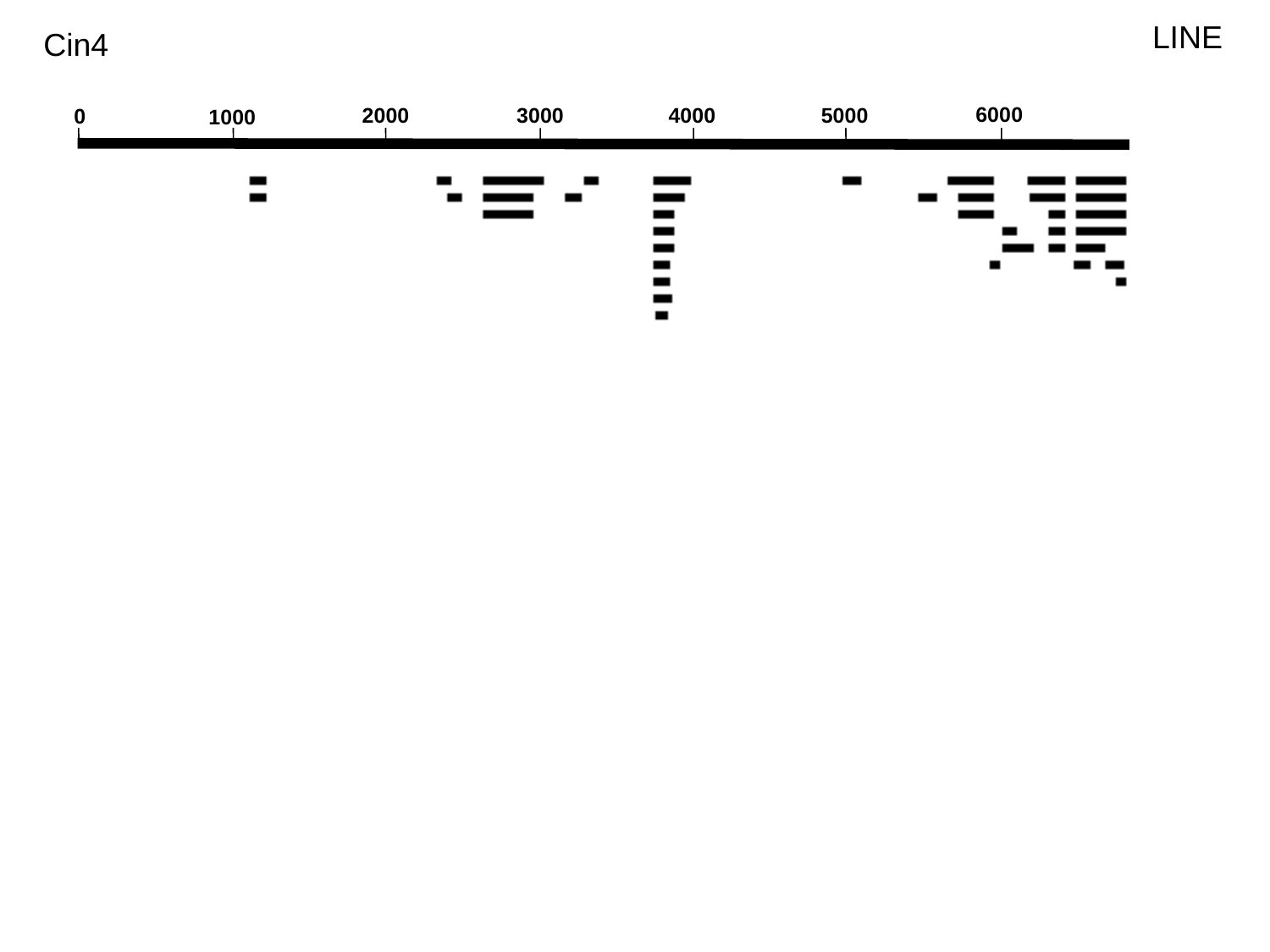

LINE
Cin4
6000
2000
5000
3000
4000
0
1000

## Slide 13
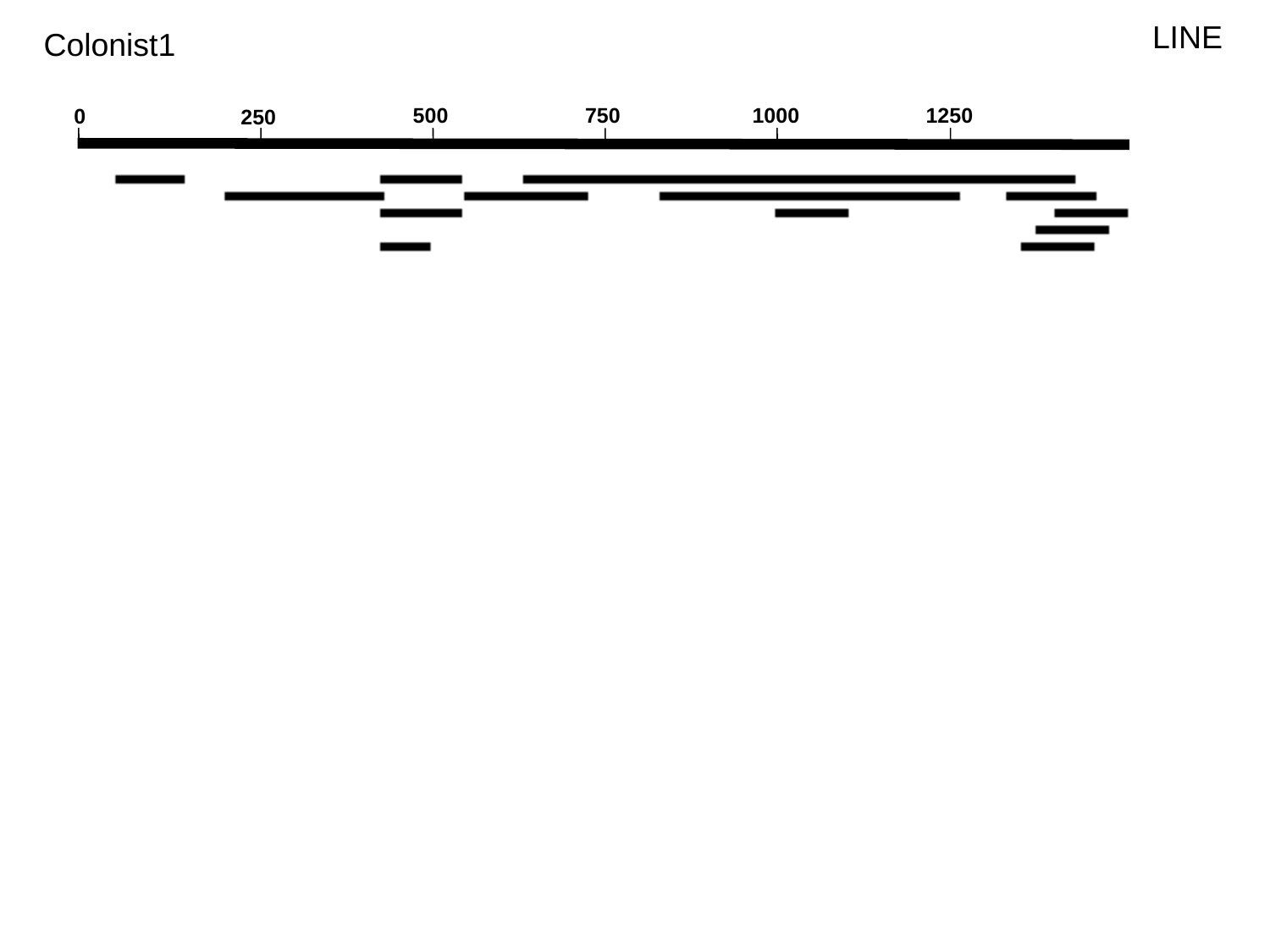

LINE
Colonist1
500
1250
750
1000
0
250

## Slide 14
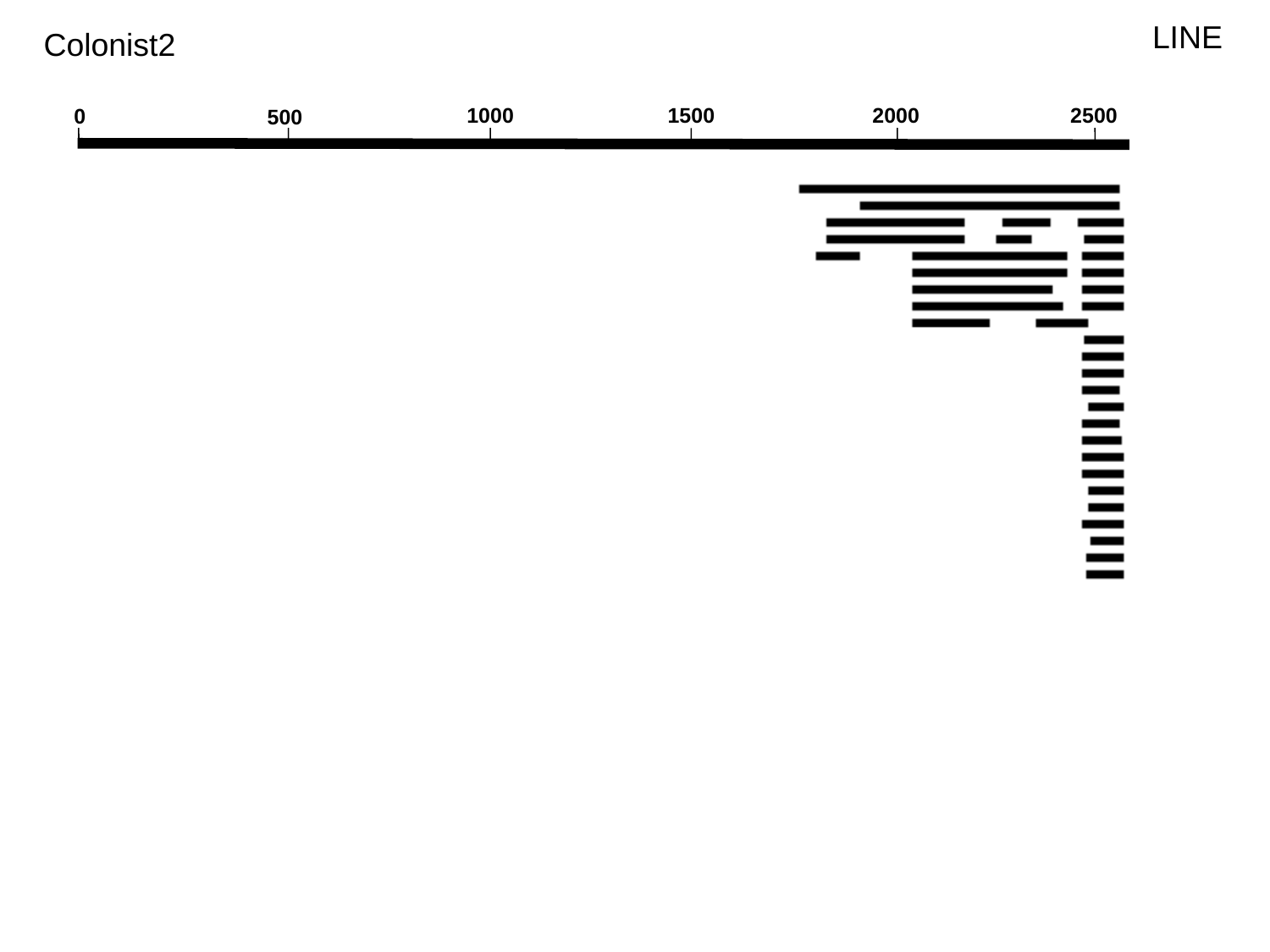

LINE
Colonist2
1000
2500
1500
2000
0
500

## Slide 15
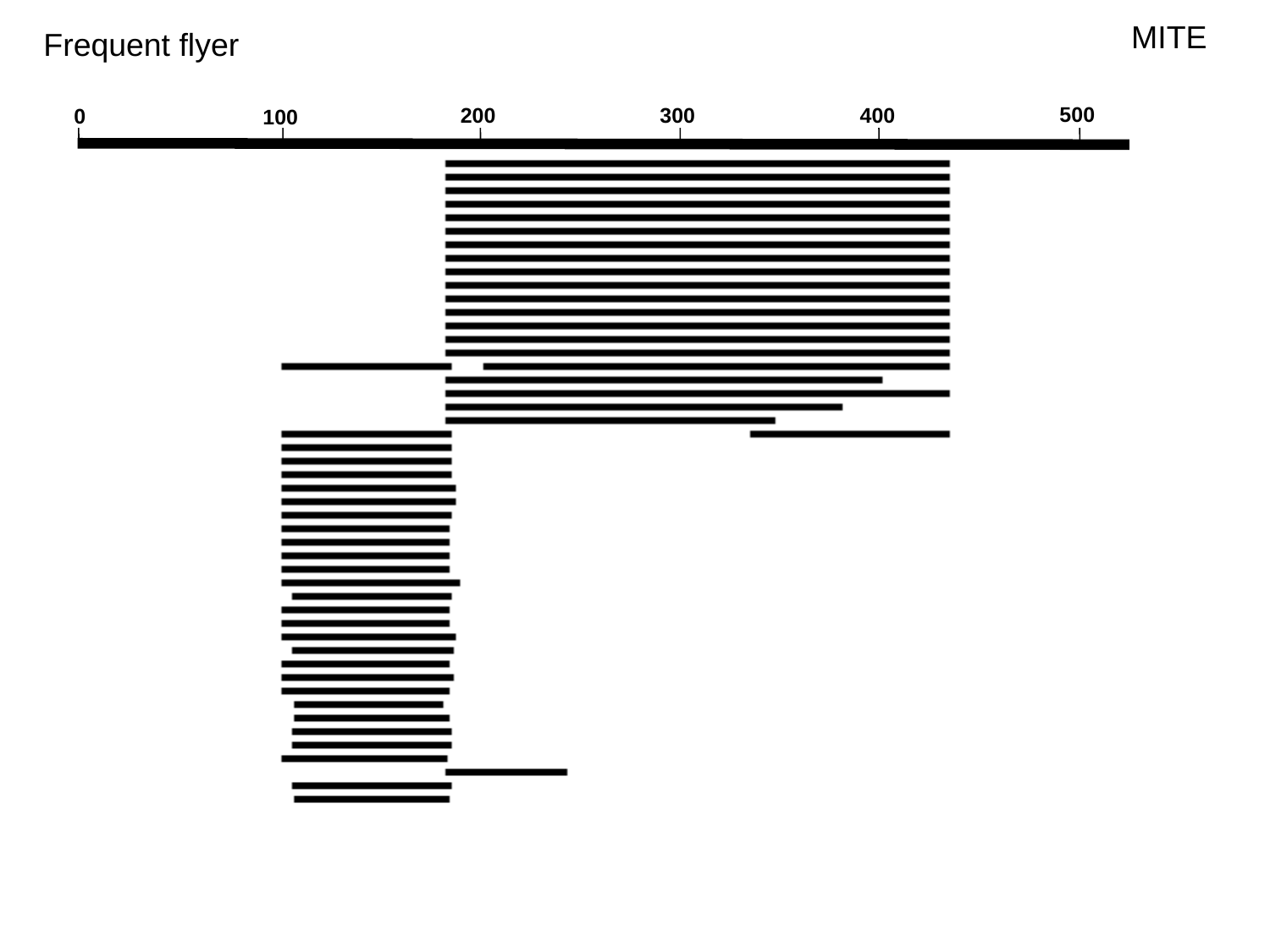

MITE
Frequent flyer
500
200
400
300
0
100

## Slide 16
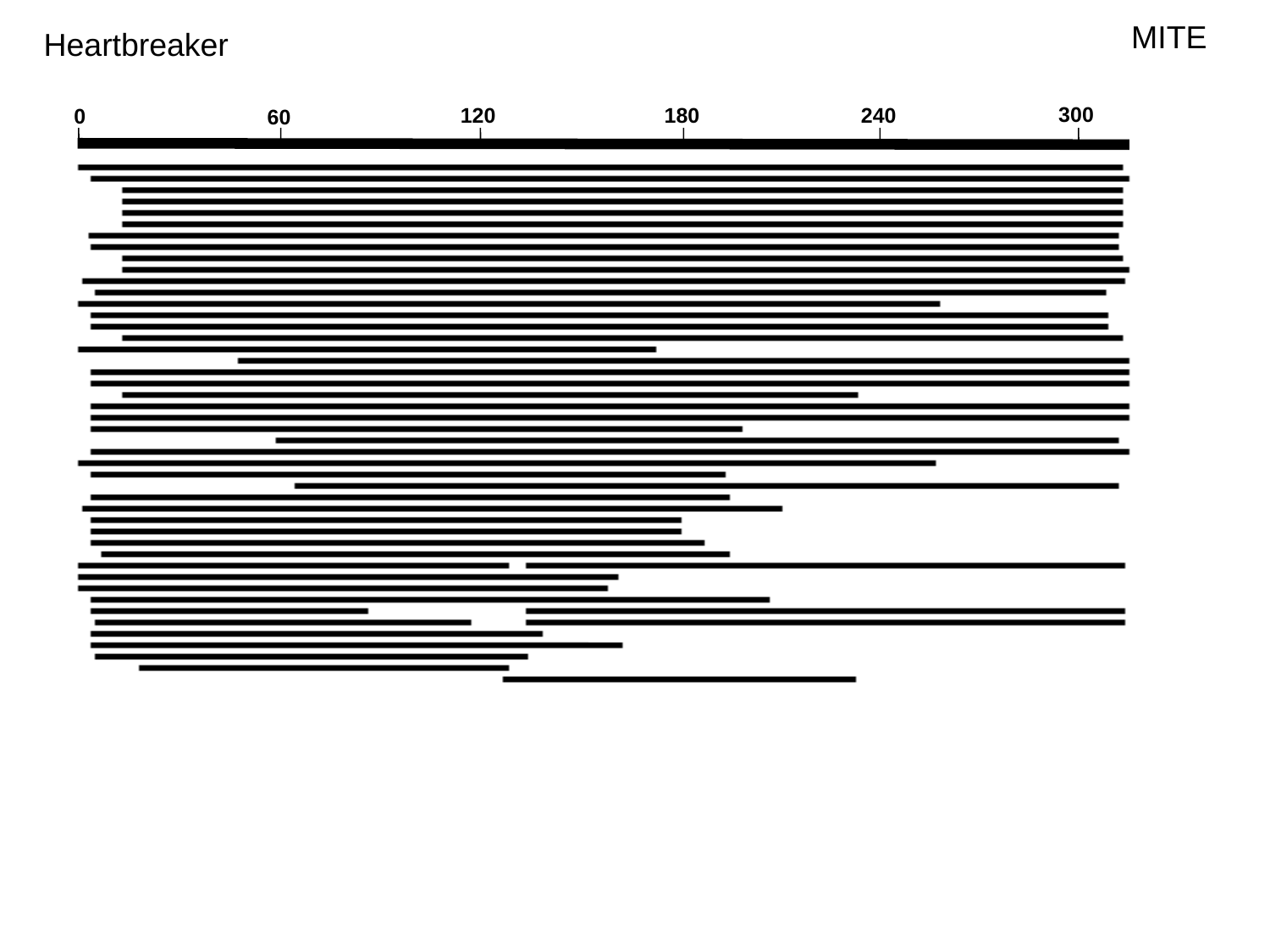

MITE
Heartbreaker
300
120
240
180
0
60

## Slide 17
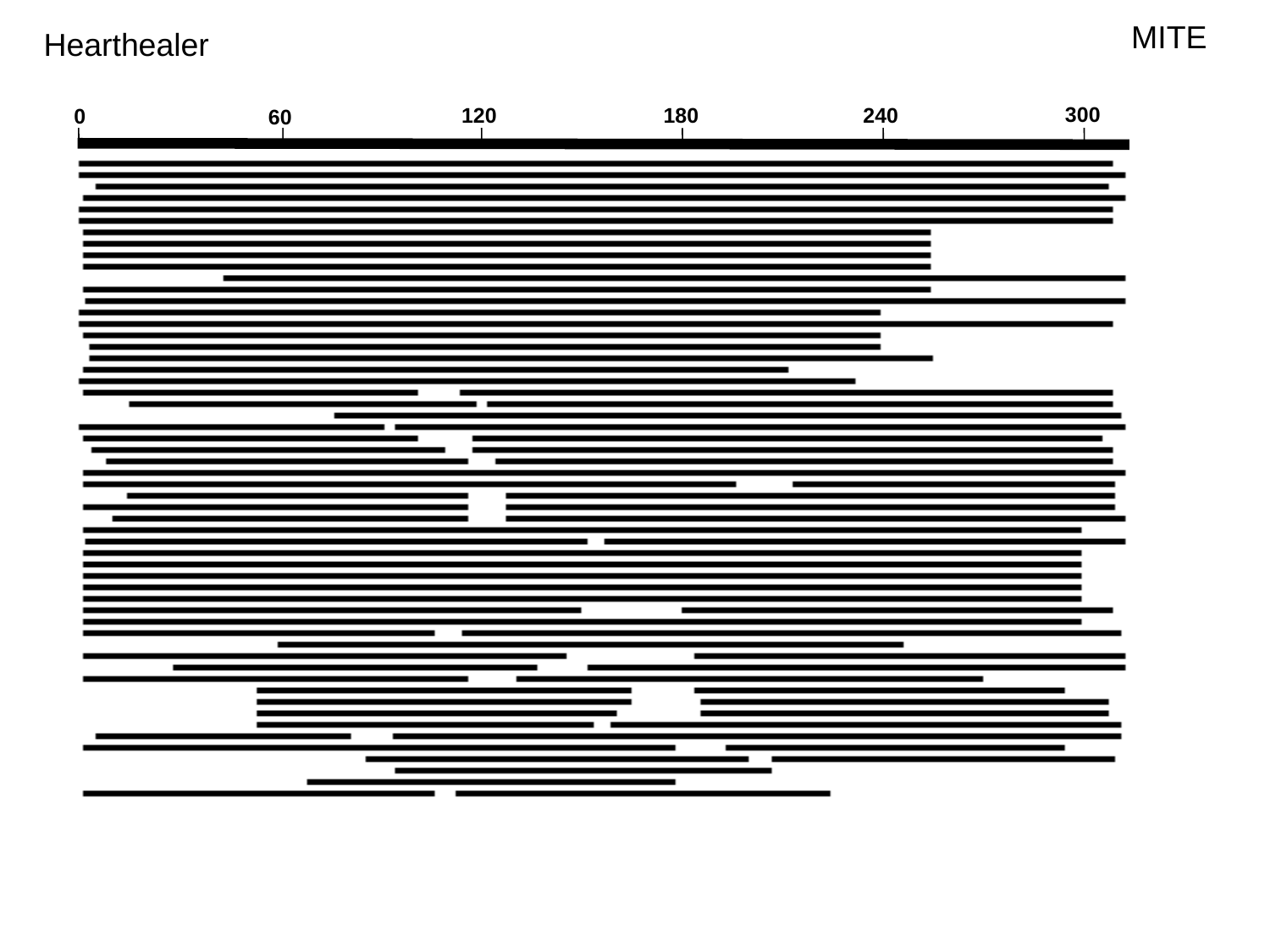

MITE
Hearthealer
300
120
240
180
0
60

## Slide 18
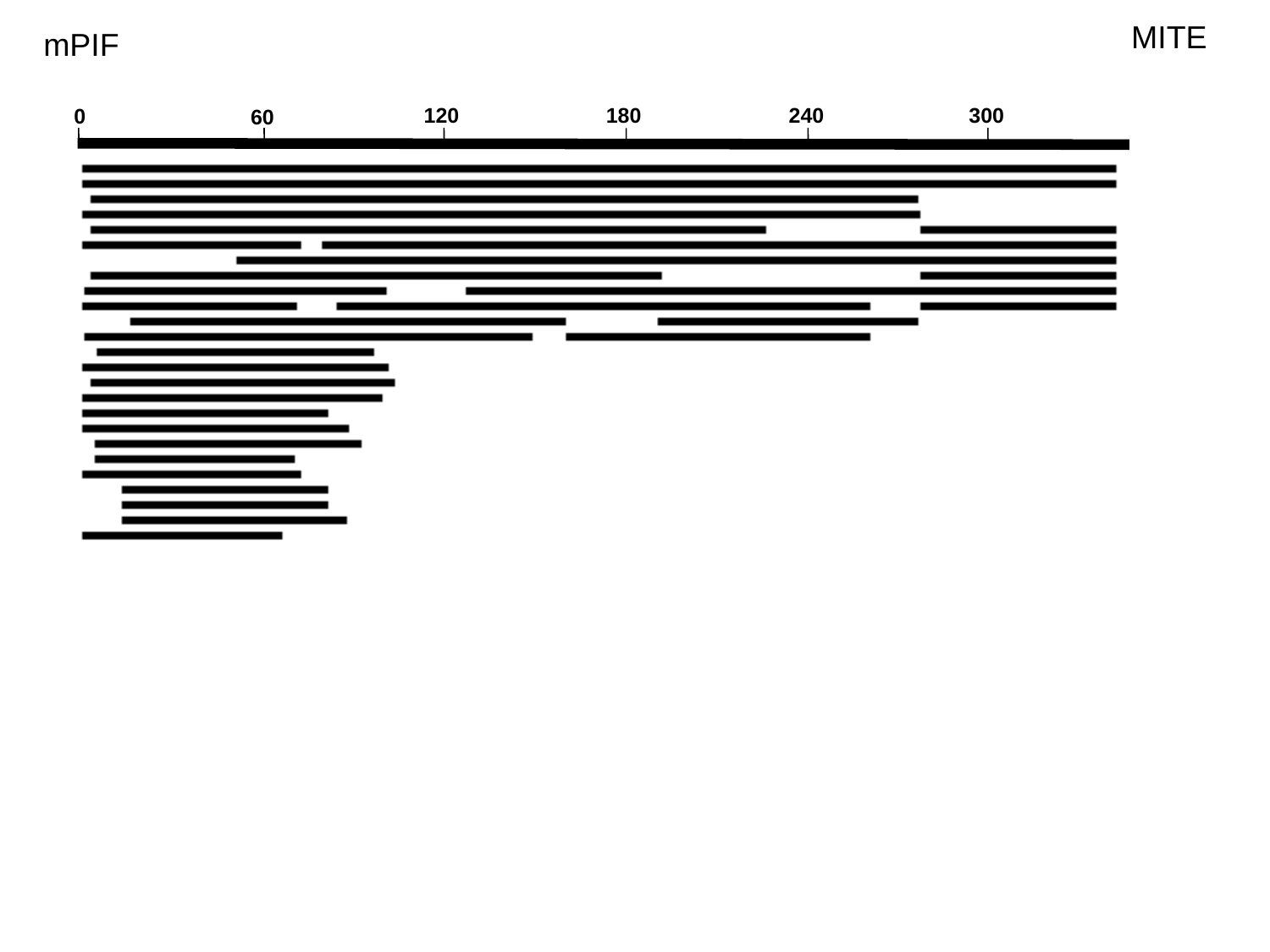

MITE
mPIF
120
300
180
240
0
60

## Slide 19
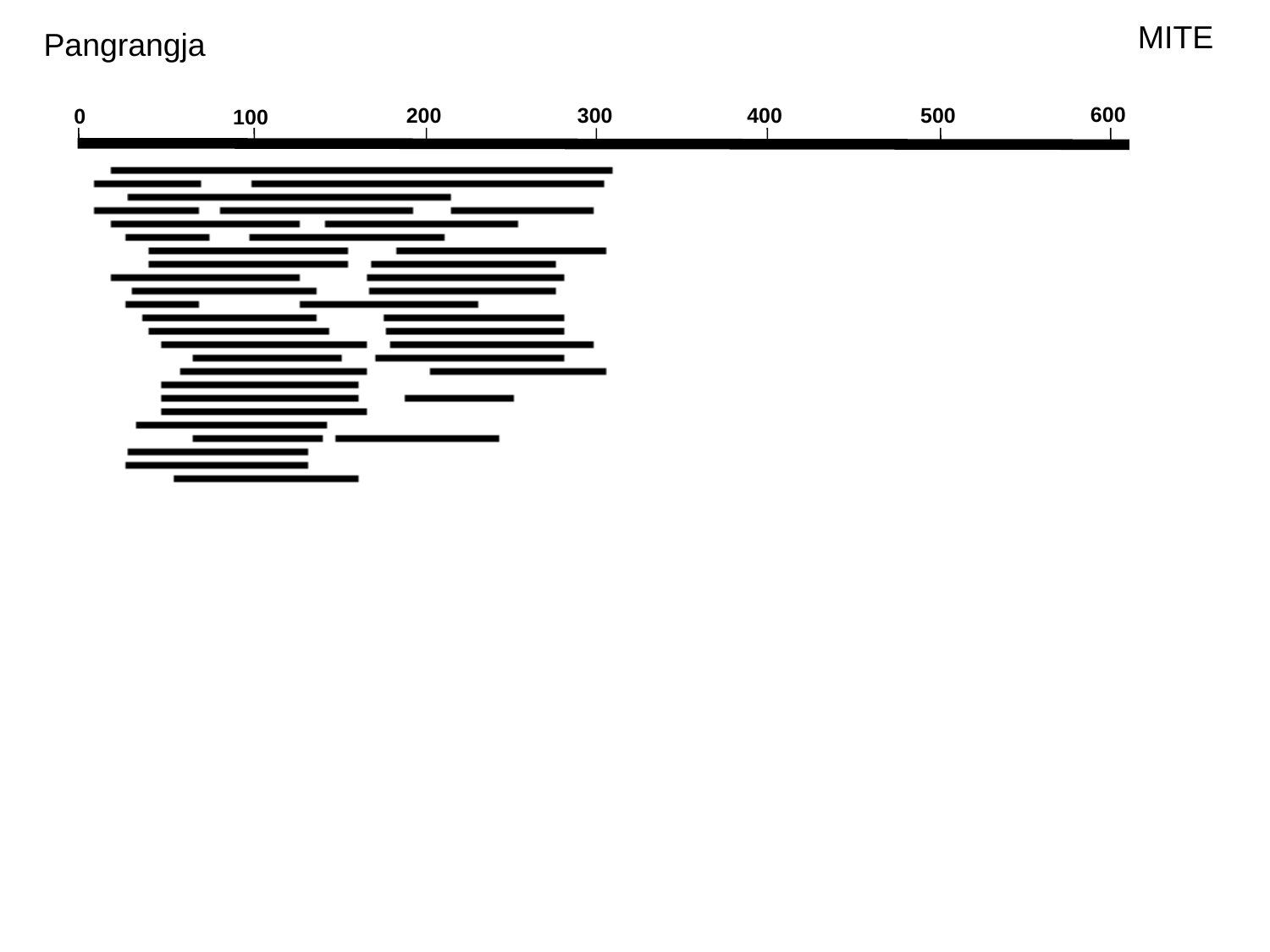

MITE
Pangrangja
600
200
500
300
400
0
100

## Slide 20
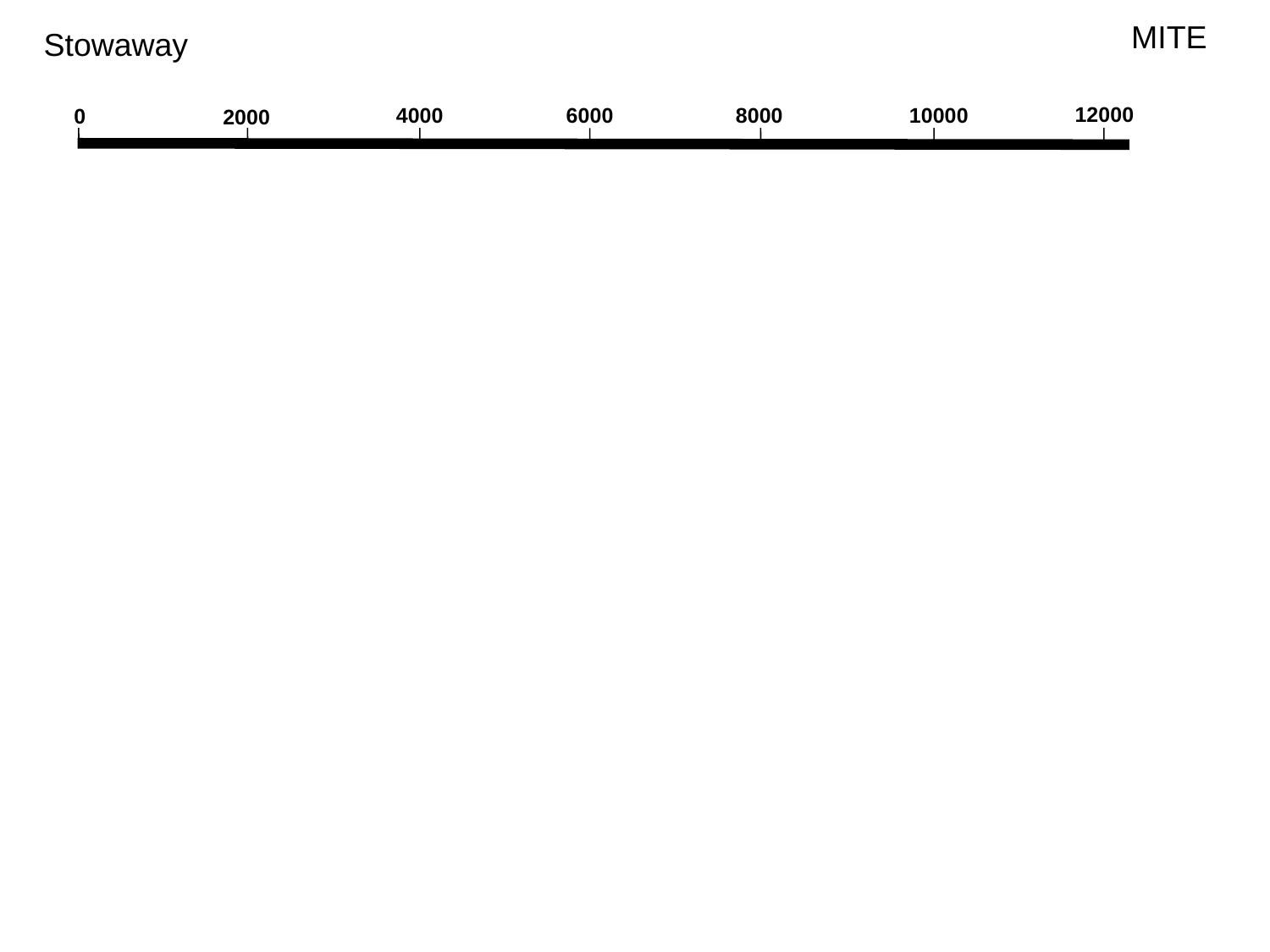

MITE
Stowaway
12000
4000
10000
6000
8000
0
2000

## Slide 21
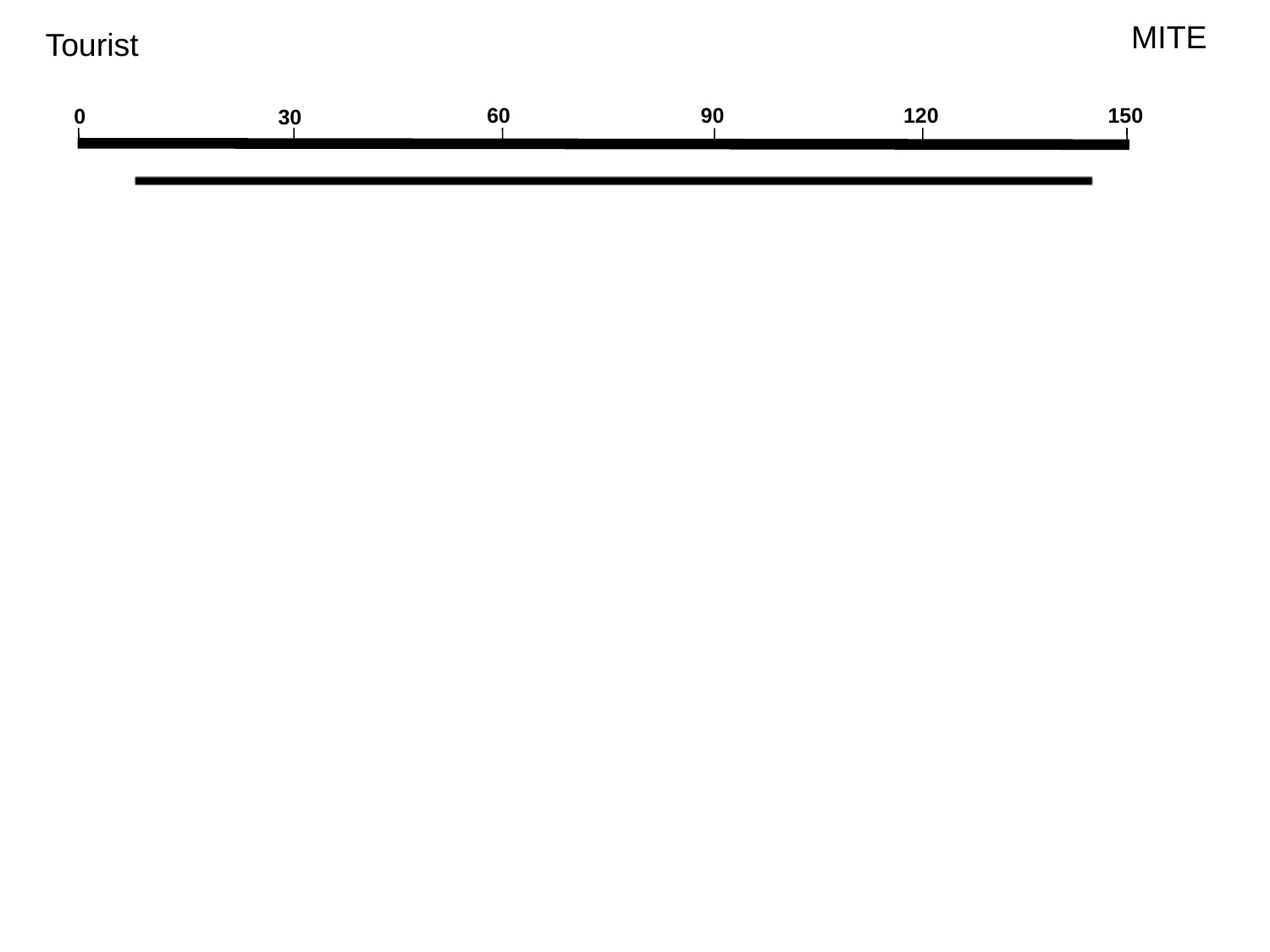

MITE
Tourist
60
150
90
120
0
30

## Slide 22
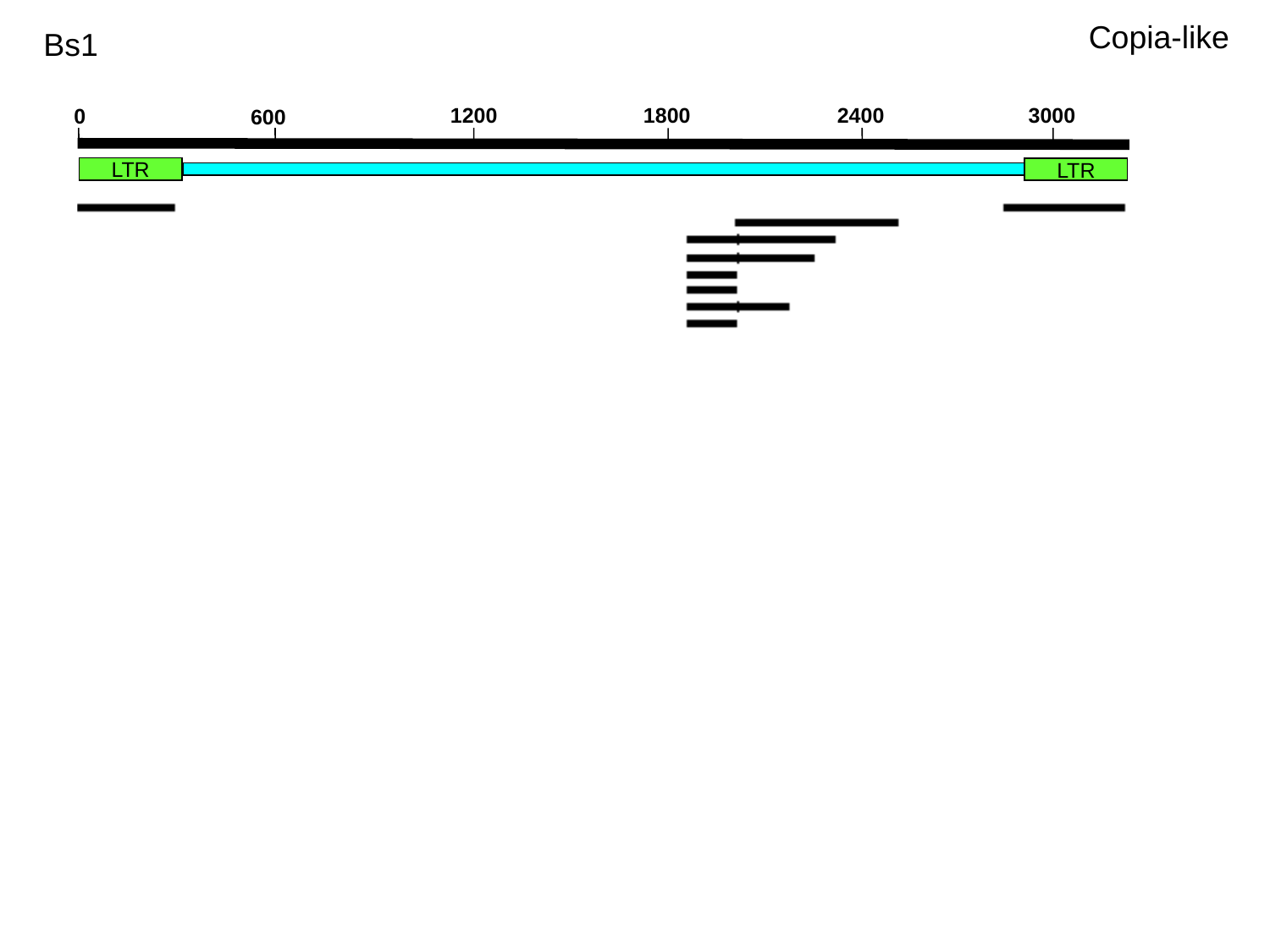

Copia-like
Bs1
1200
3000
1800
2400
0
600
LTR
LTR

## Slide 23
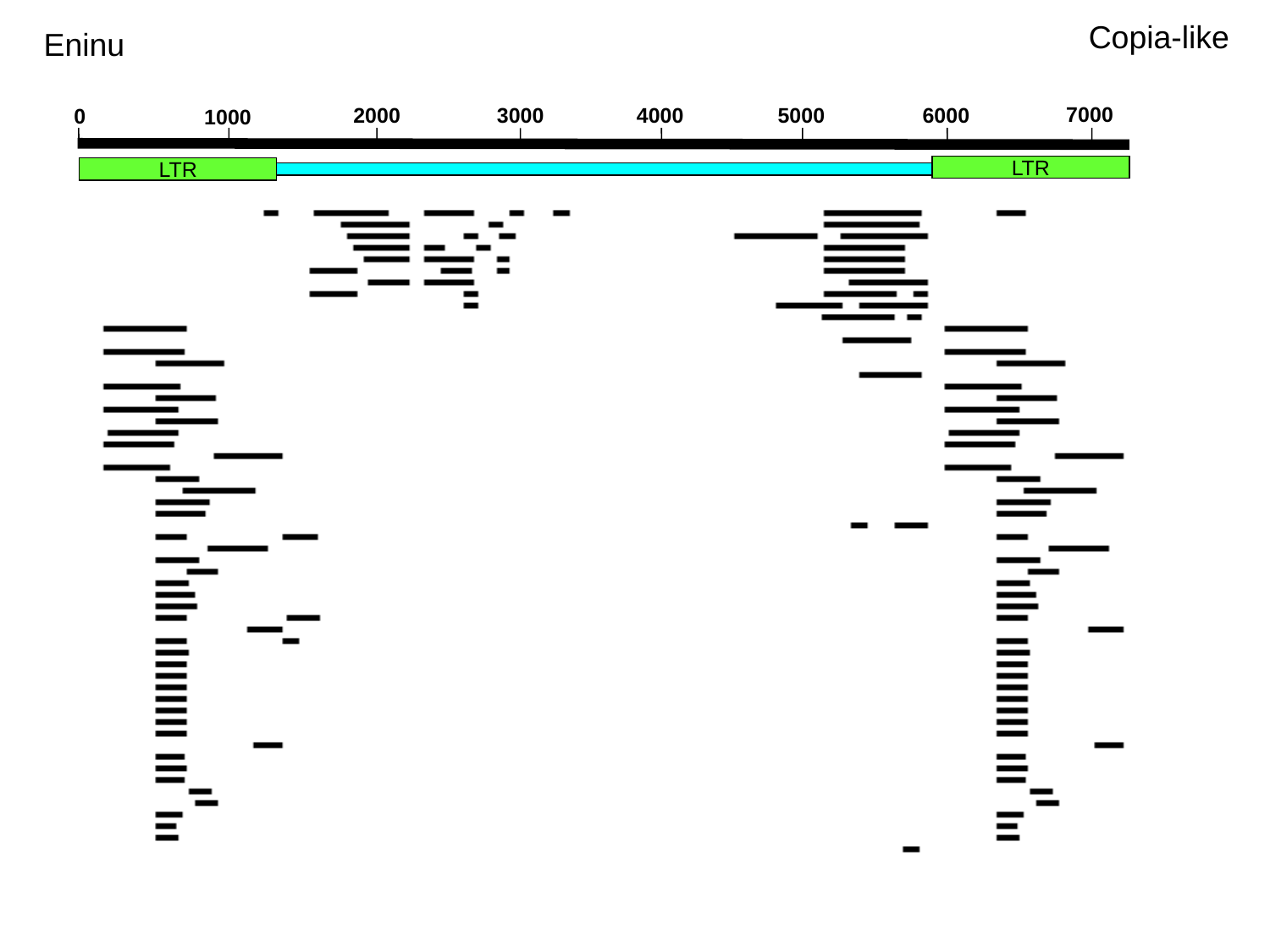

Copia-like
Eninu
7000
2000
6000
3000
4000
5000
0
1000
LTR
LTR

## Slide 24
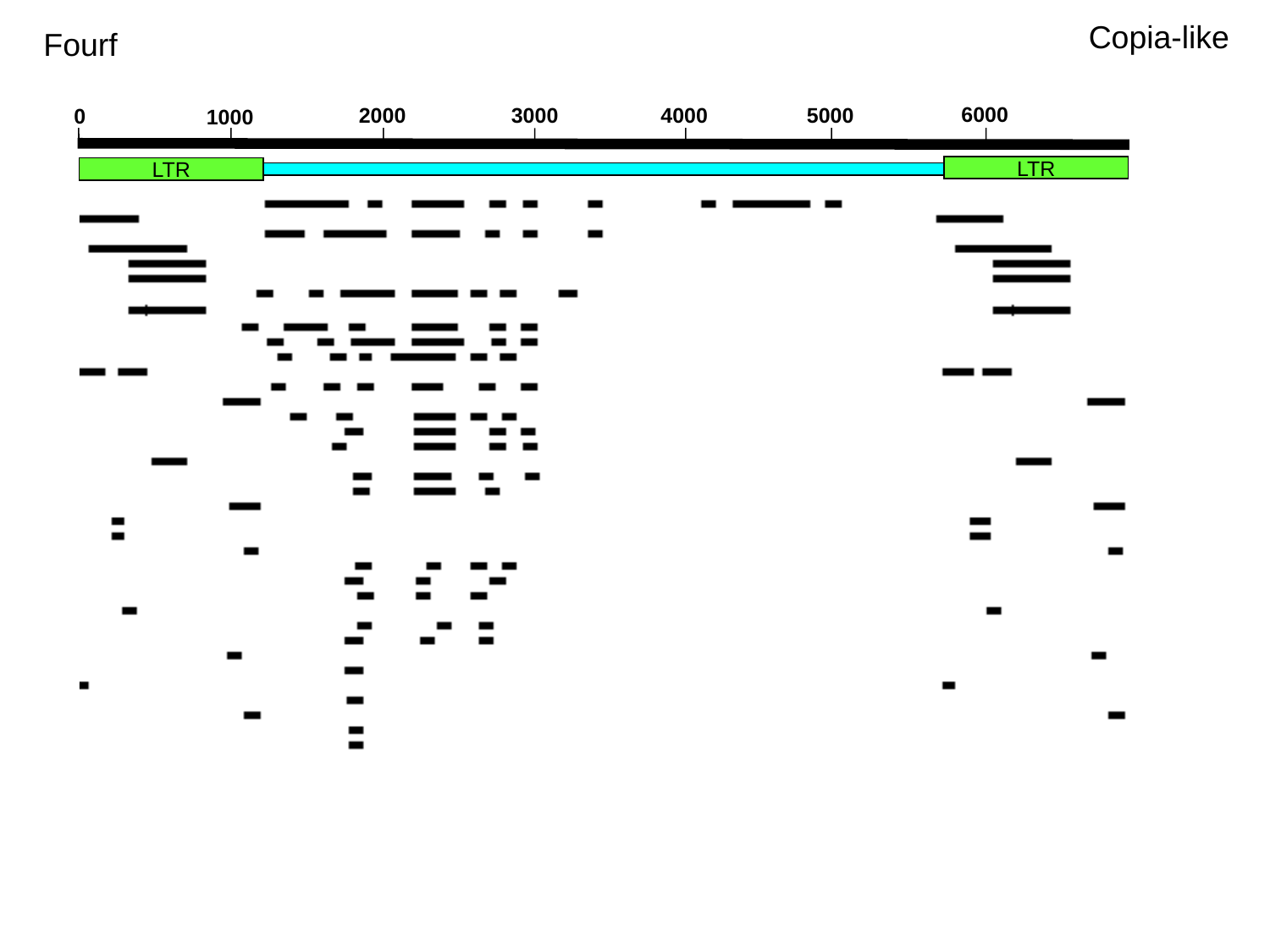

Copia-like
Fourf
6000
2000
5000
3000
4000
0
1000
LTR
LTR

## Slide 25
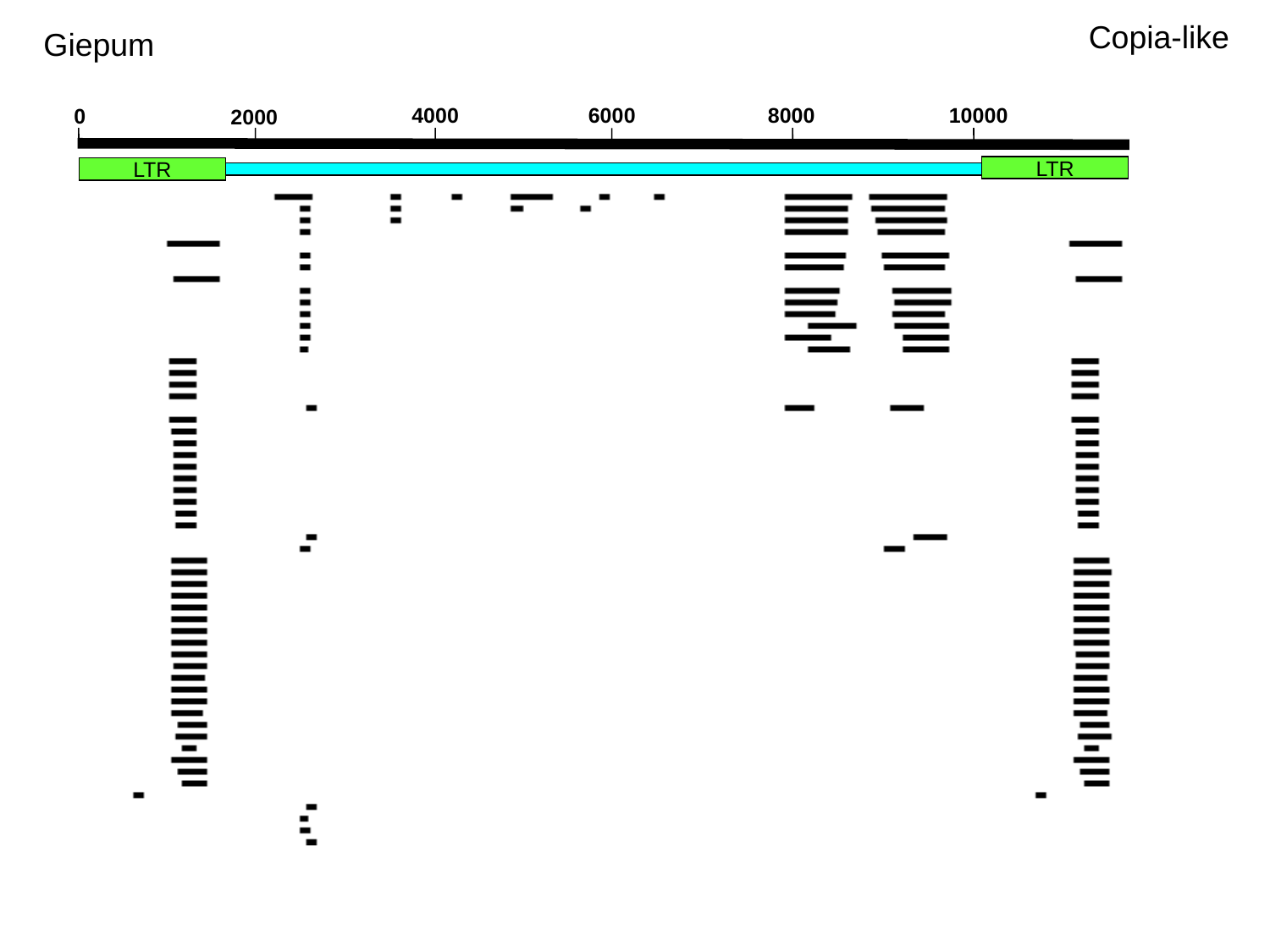

Copia-like
Giepum
4000
10000
6000
8000
0
2000
LTR
LTR

## Slide 26
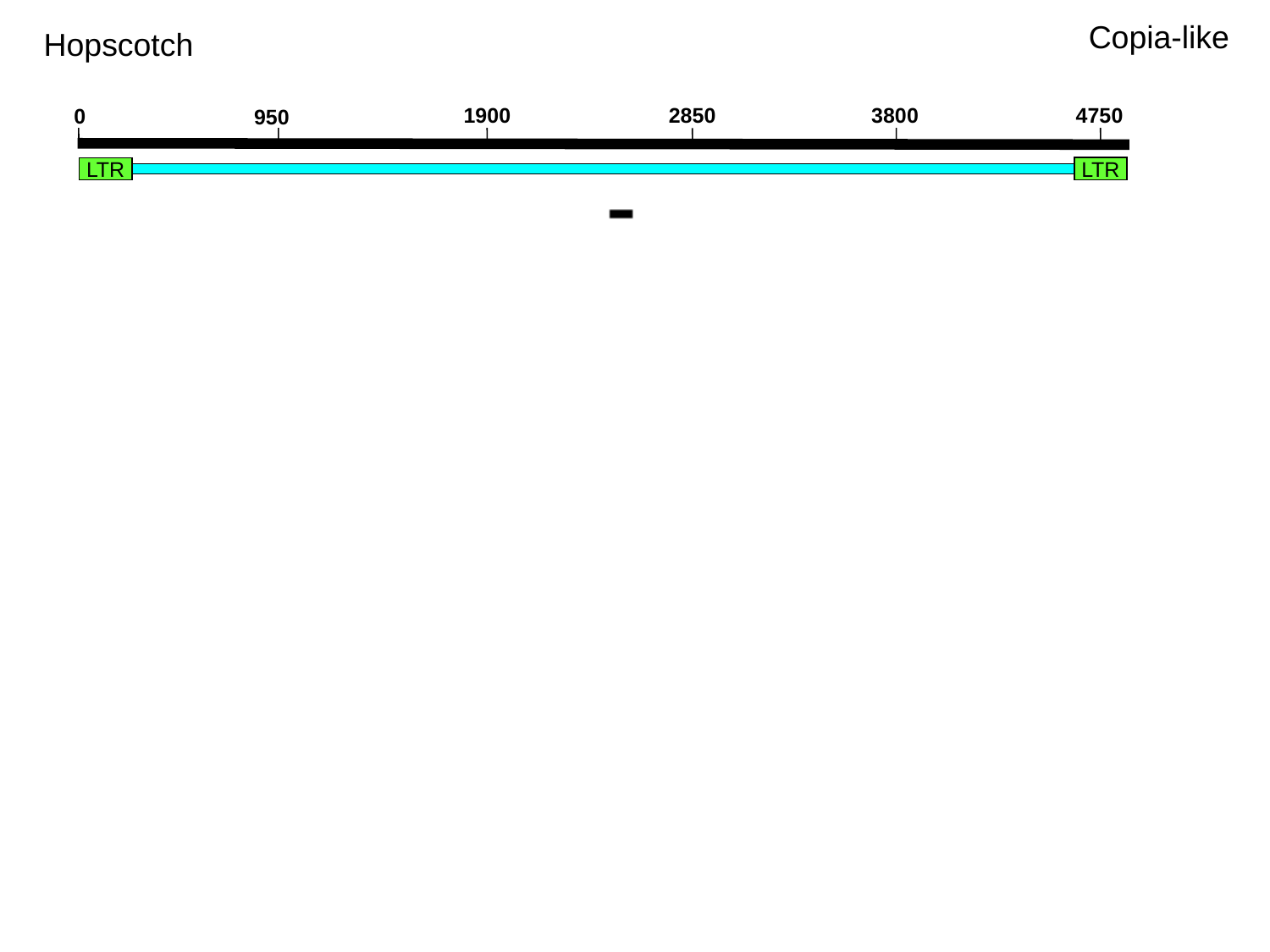

Copia-like
Hopscotch
1900
4750
2850
3800
0
950
LTR
LTR

## Slide 27
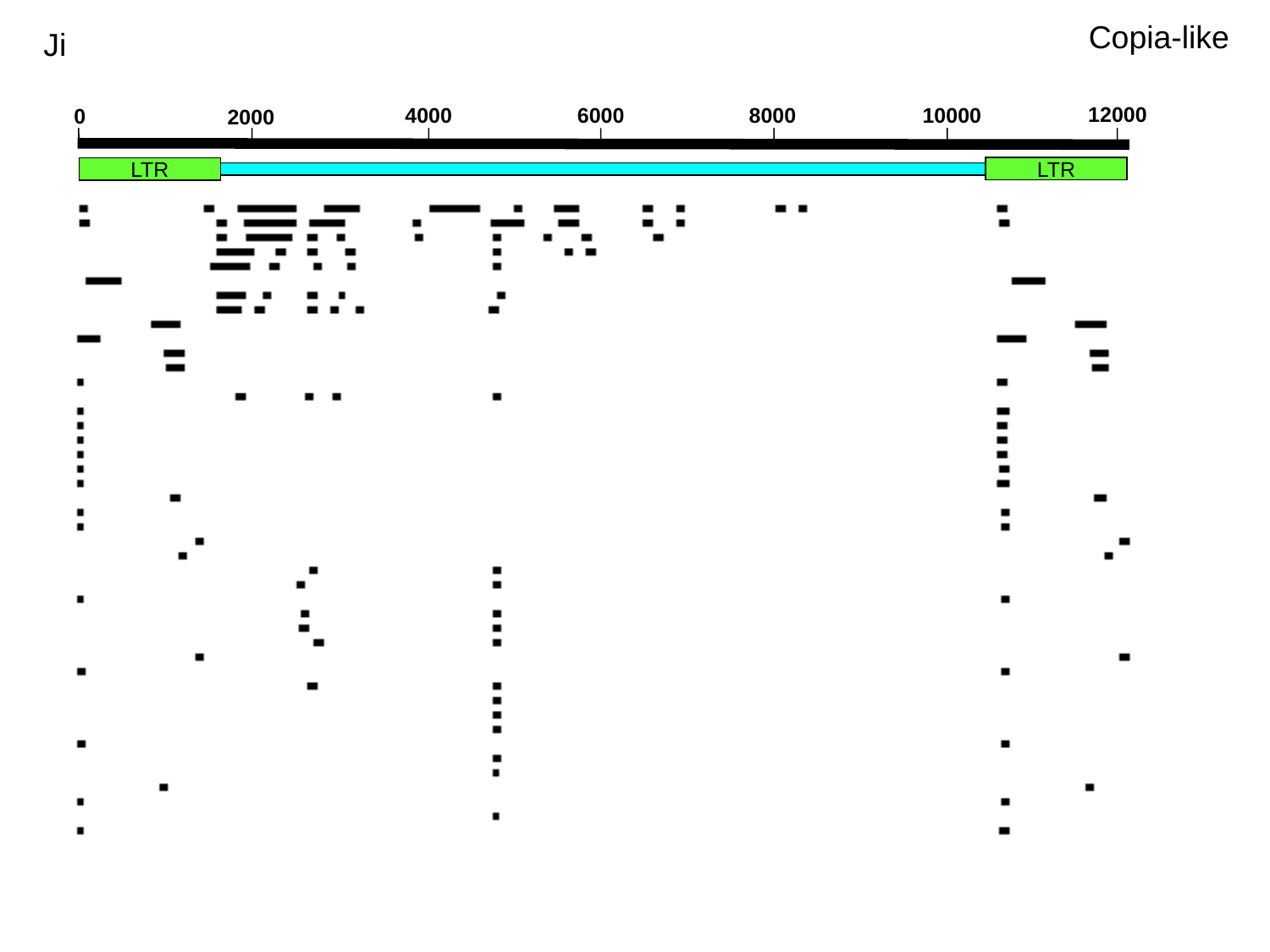

Copia-like
Ji
12000
4000
10000
6000
8000
0
2000
LTR
LTR

## Slide 28
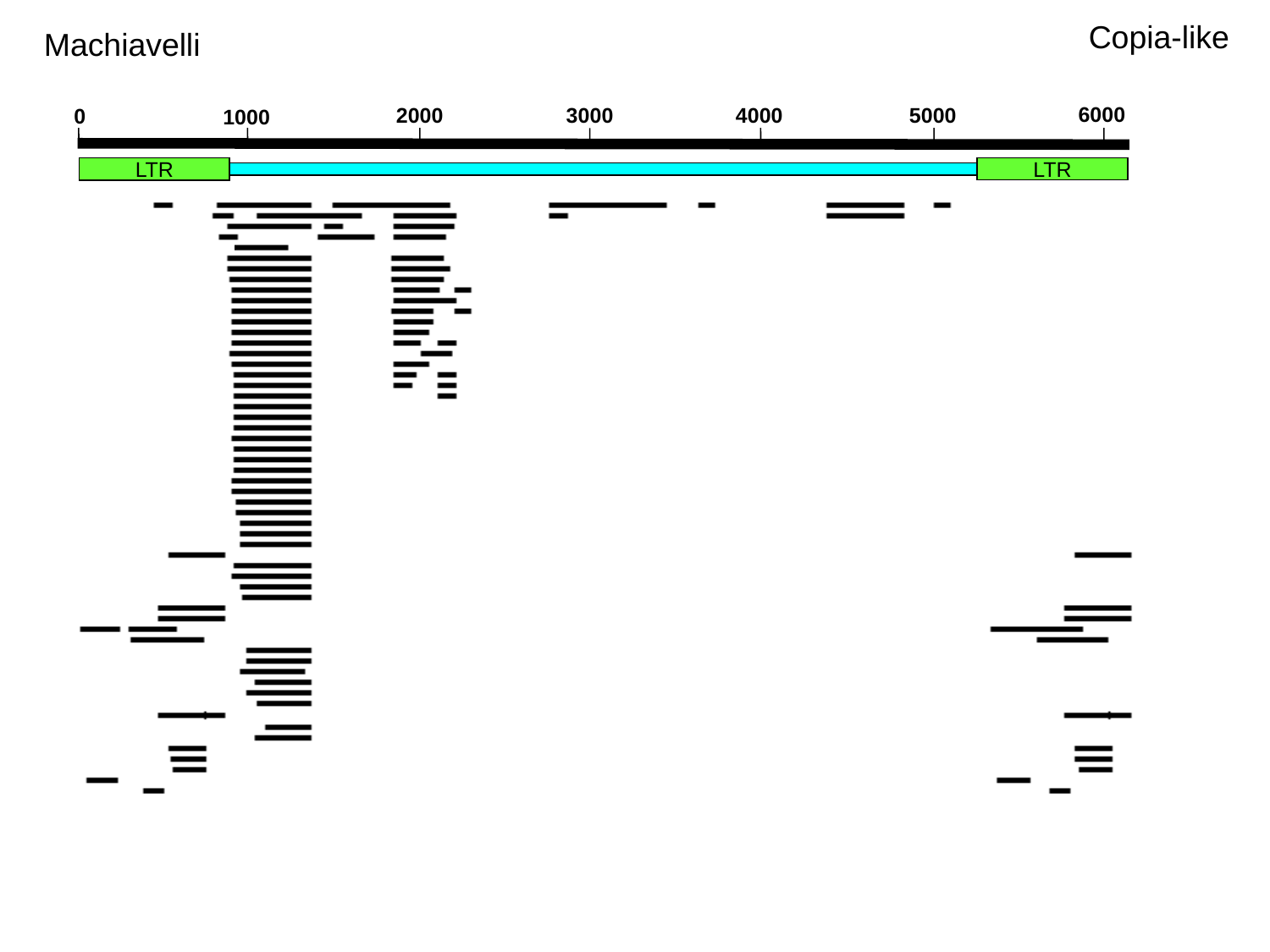

Copia-like
Machiavelli
6000
2000
5000
3000
4000
0
1000
LTR
LTR

## Slide 29
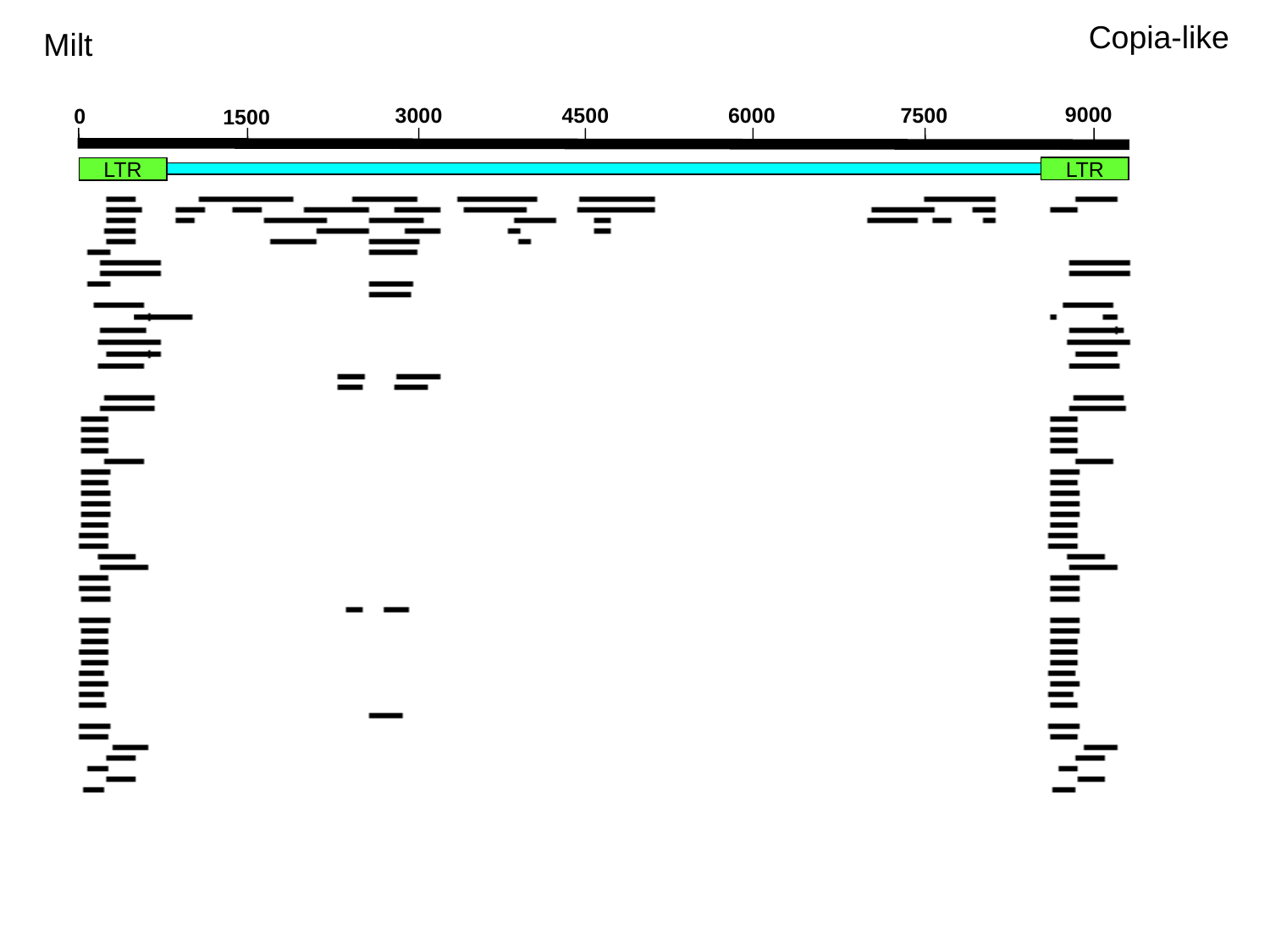

Copia-like
Milt
9000
3000
7500
4500
6000
0
1500
LTR
LTR

## Slide 30
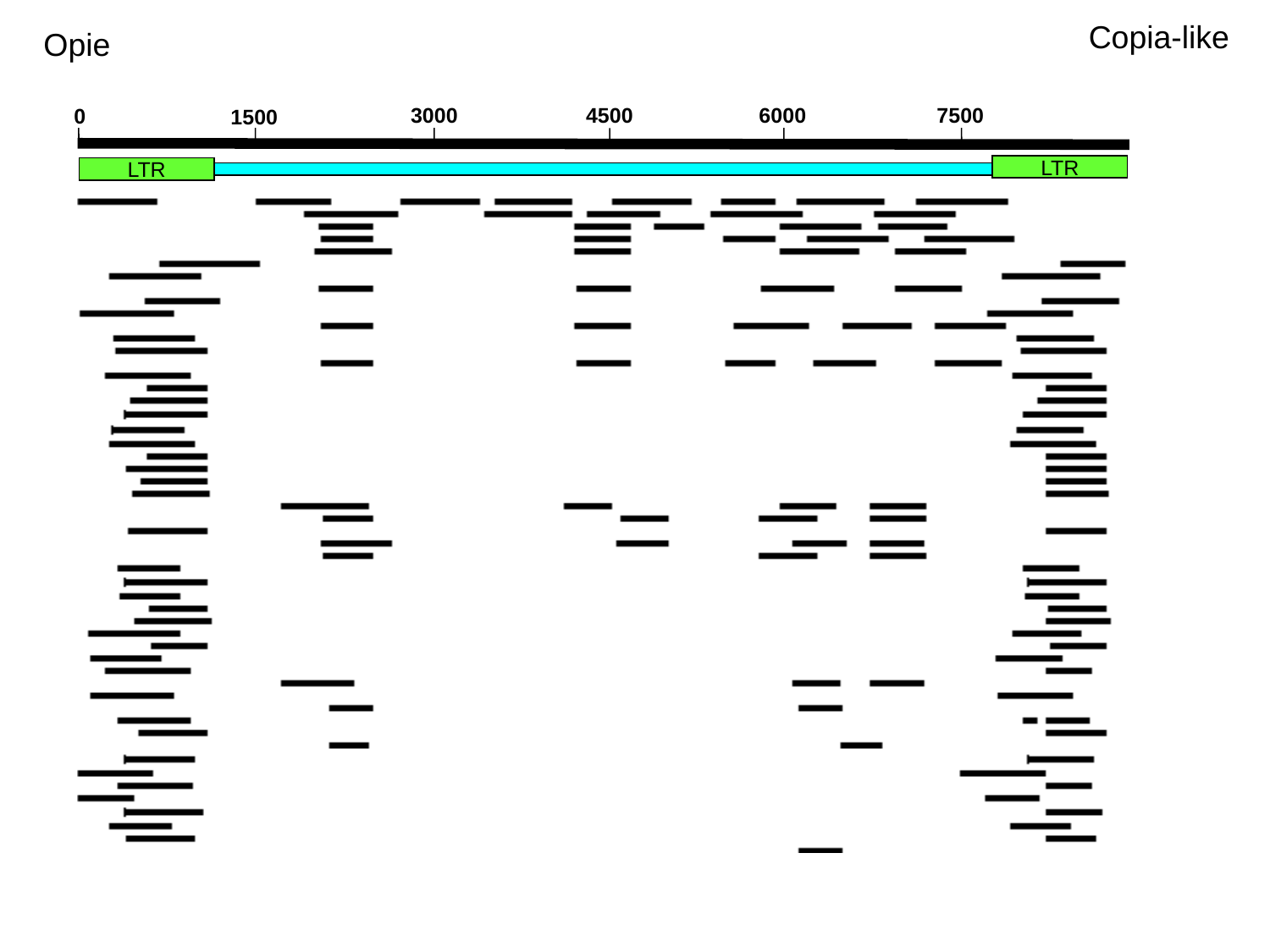

Copia-like
Opie
3000
7500
4500
6000
0
1500
LTR
LTR

## Slide 31
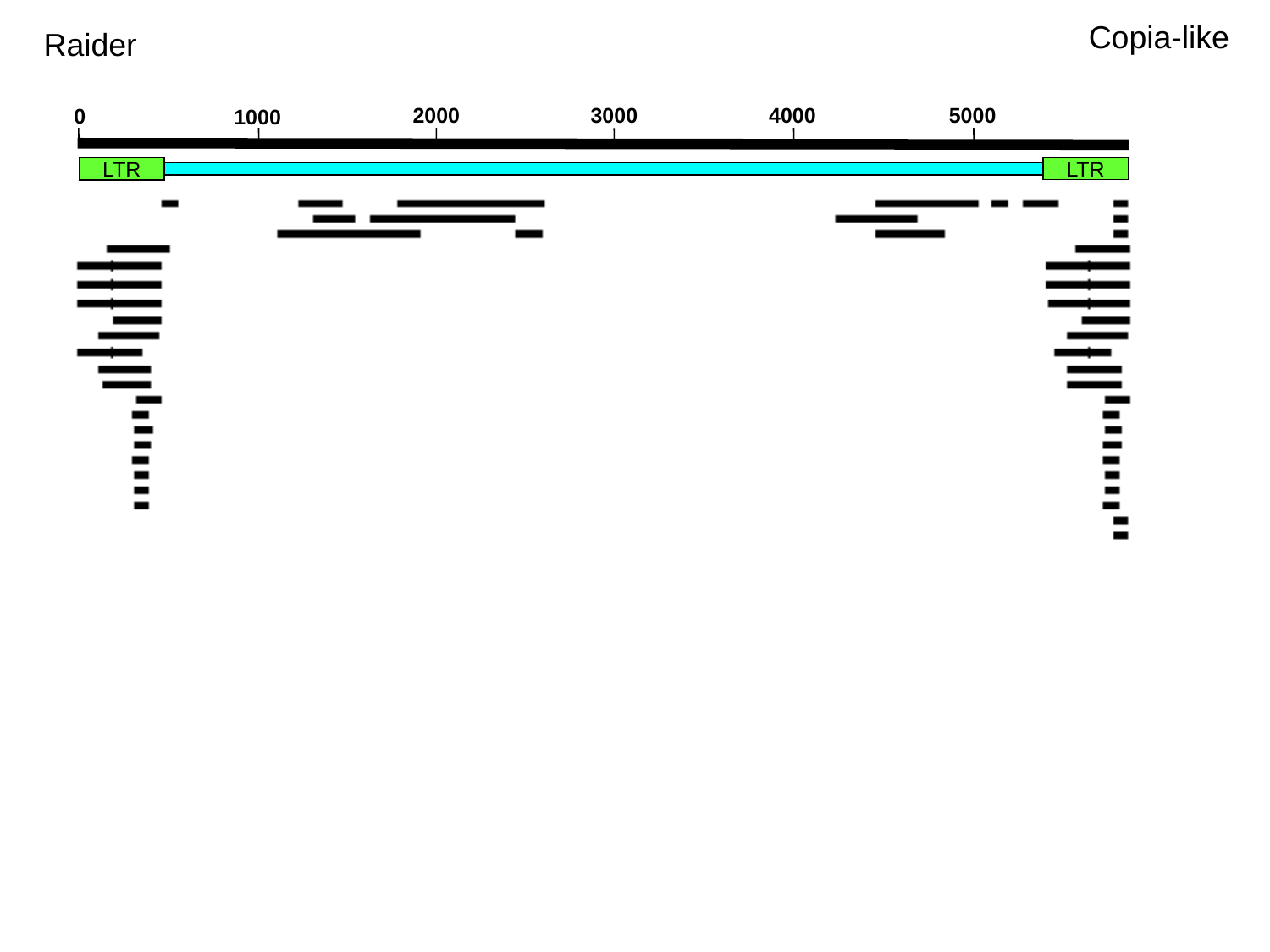

Copia-like
Raider
2000
5000
3000
4000
0
1000
LTR
LTR

## Slide 32
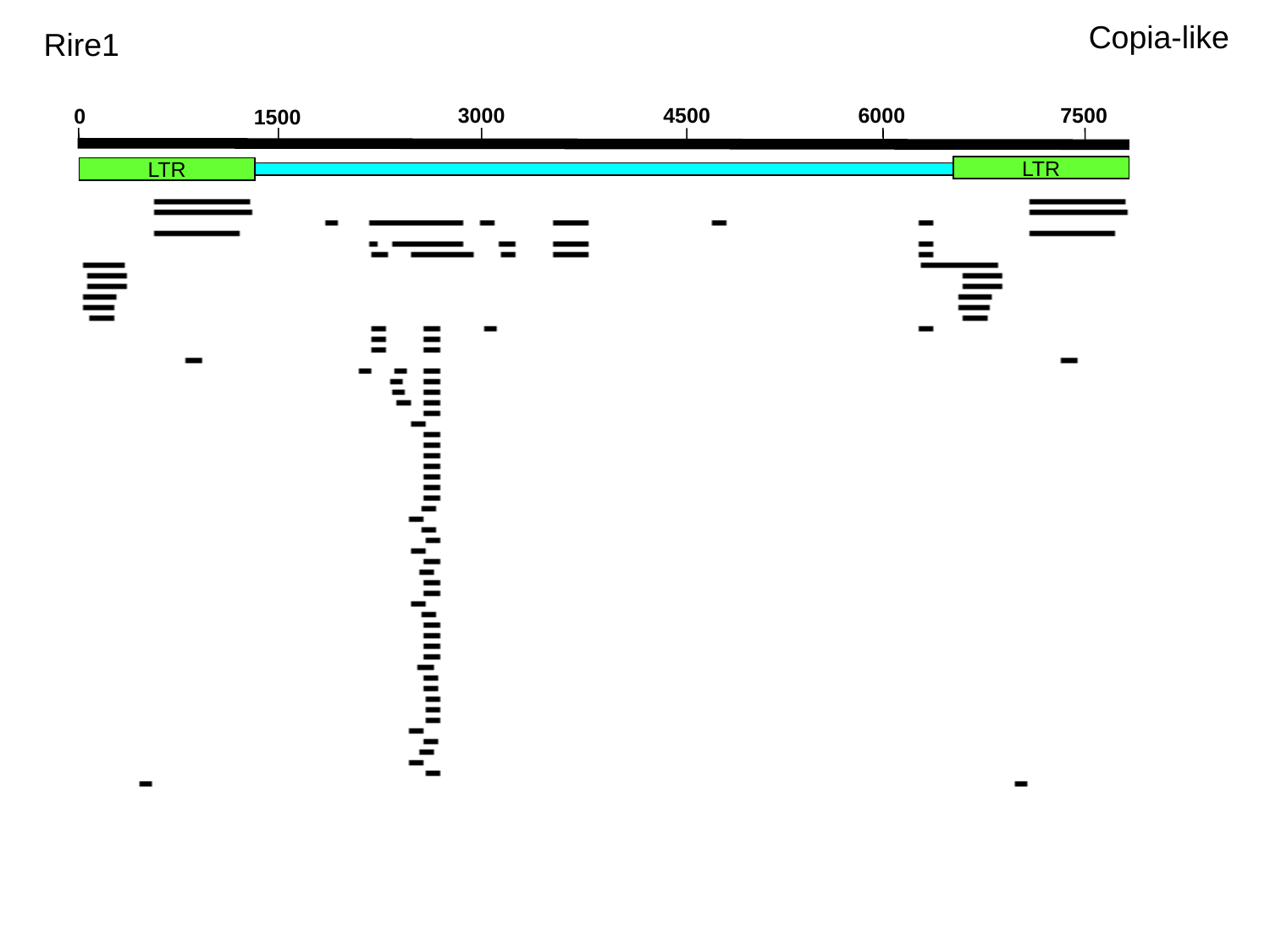

Copia-like
Rire1
3000
7500
4500
6000
0
1500
LTR
LTR

## Slide 33
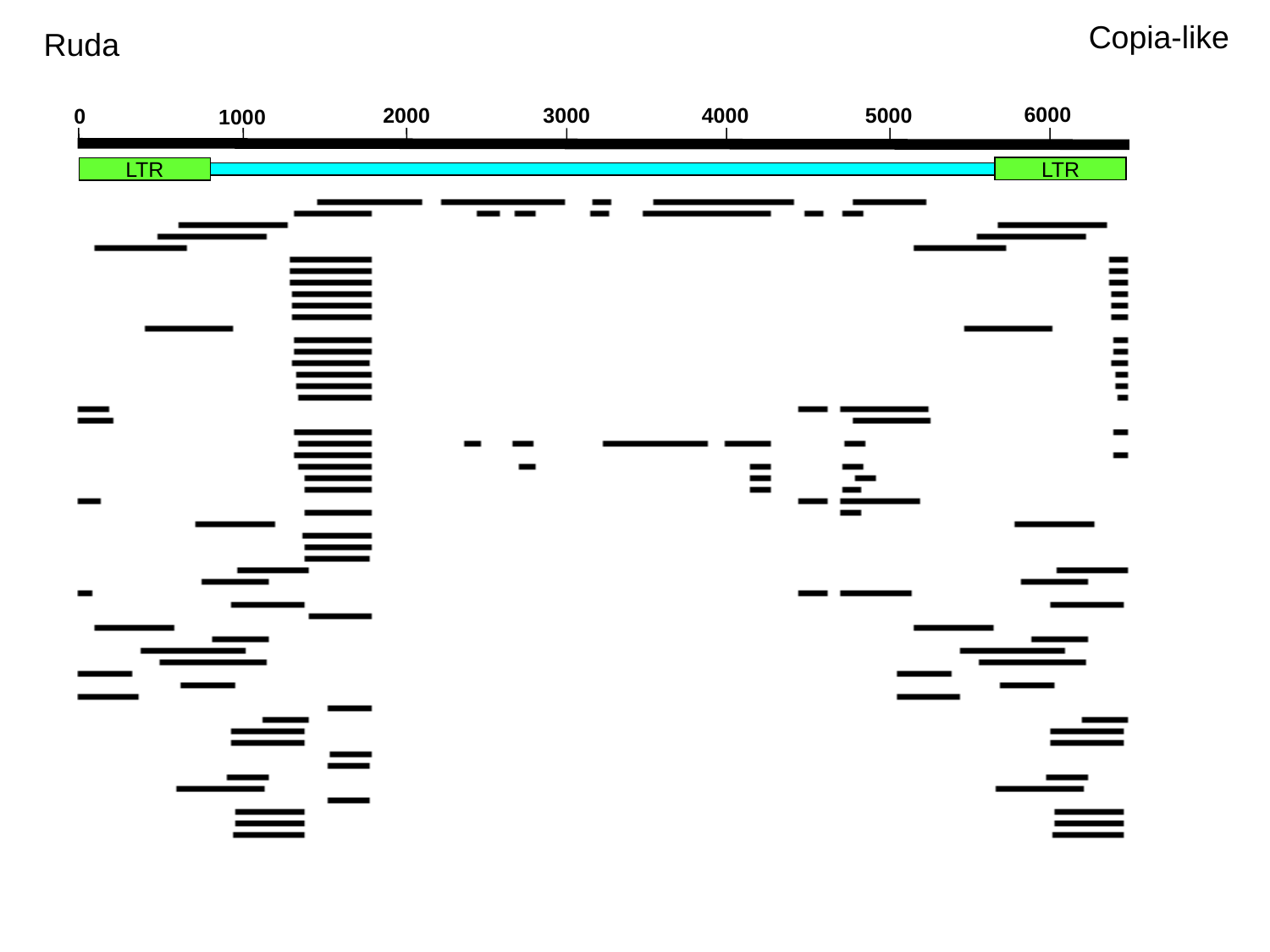

Copia-like
Ruda
6000
2000
5000
3000
4000
0
1000
LTR
LTR

## Slide 34
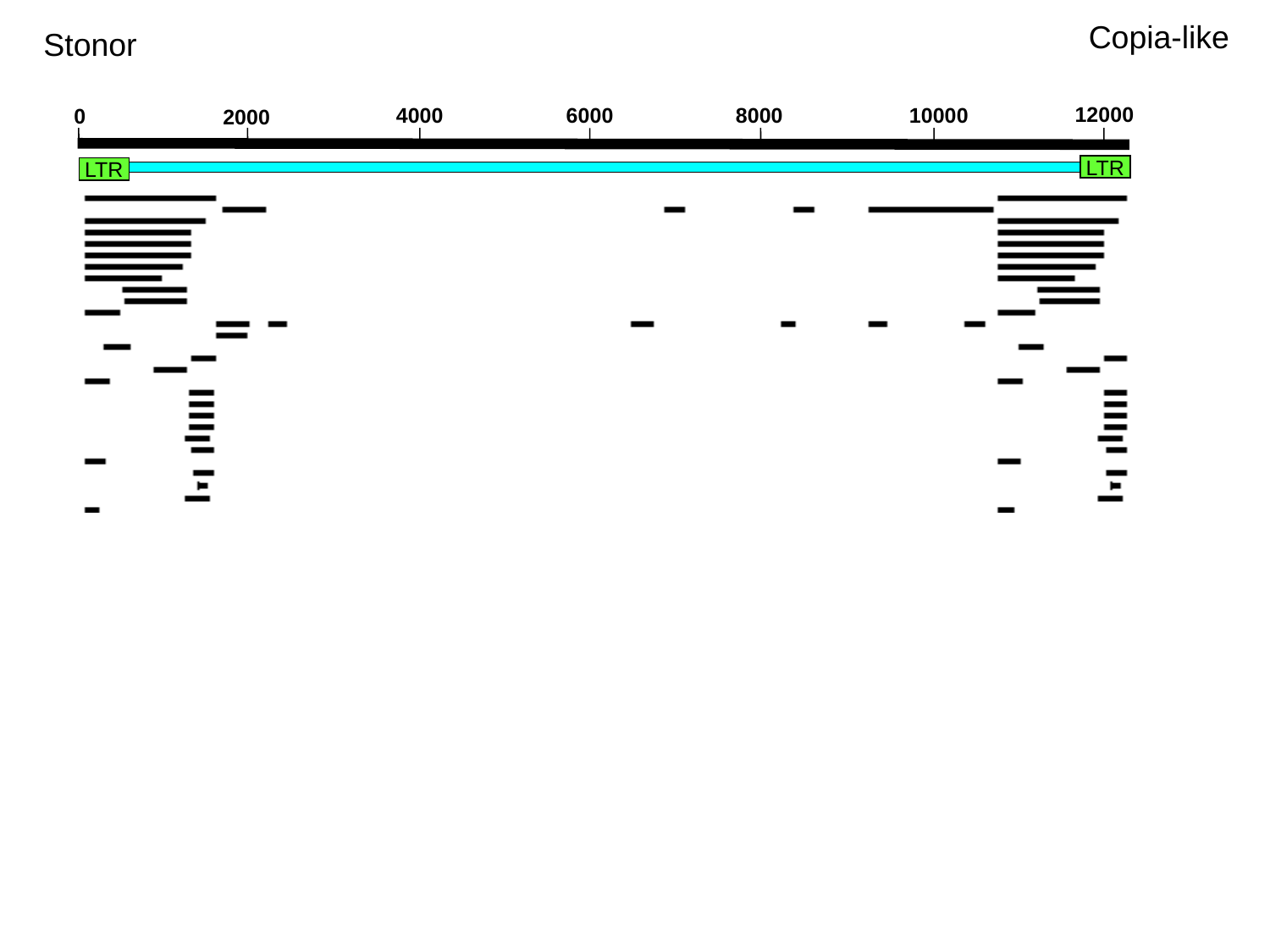

Copia-like
Stonor
12000
4000
10000
6000
8000
0
2000
LTR
LTR

## Slide 35
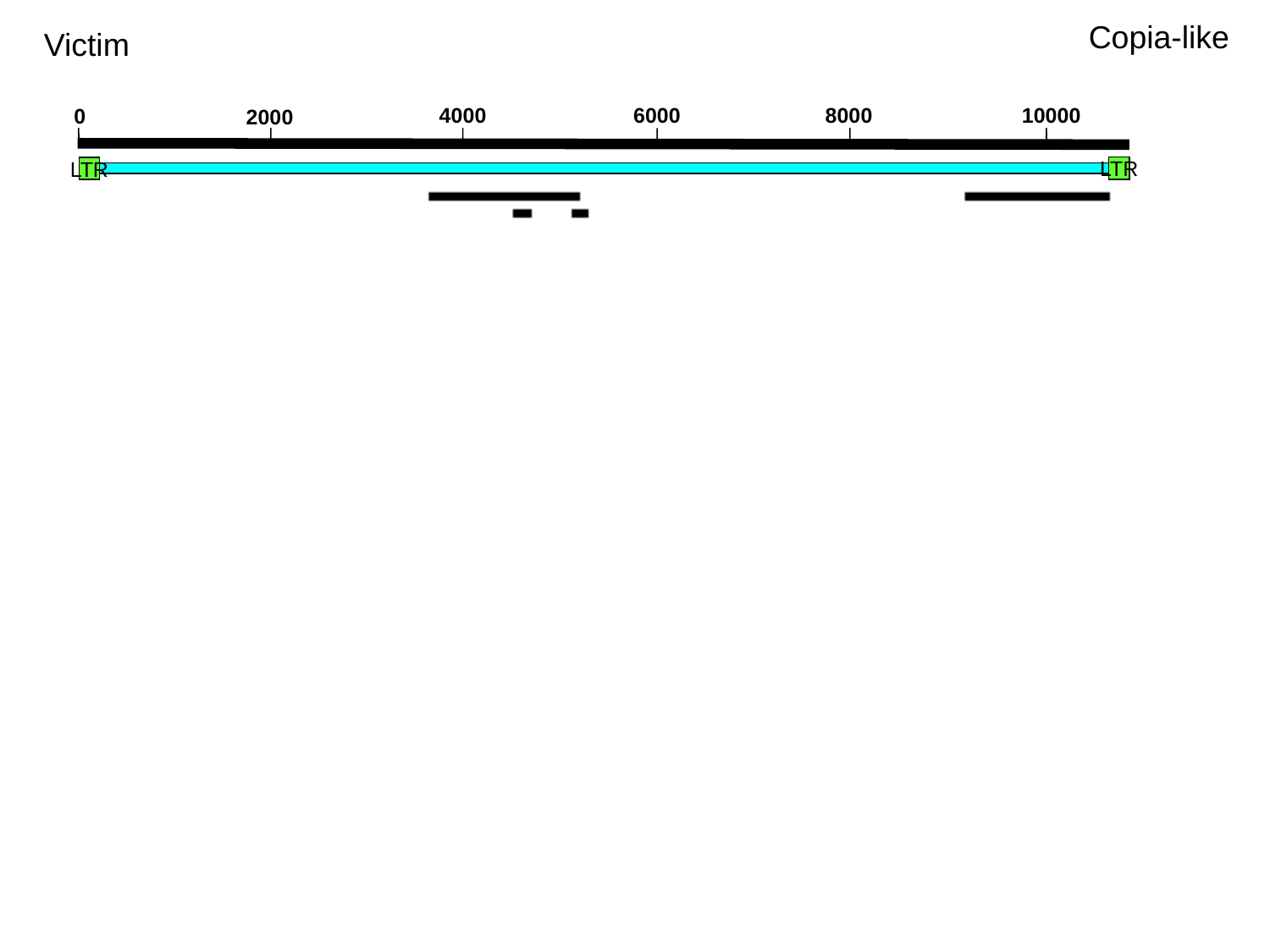

Copia-like
Victim
4000
10000
6000
8000
0
2000
LTR
LTR

## Slide 36
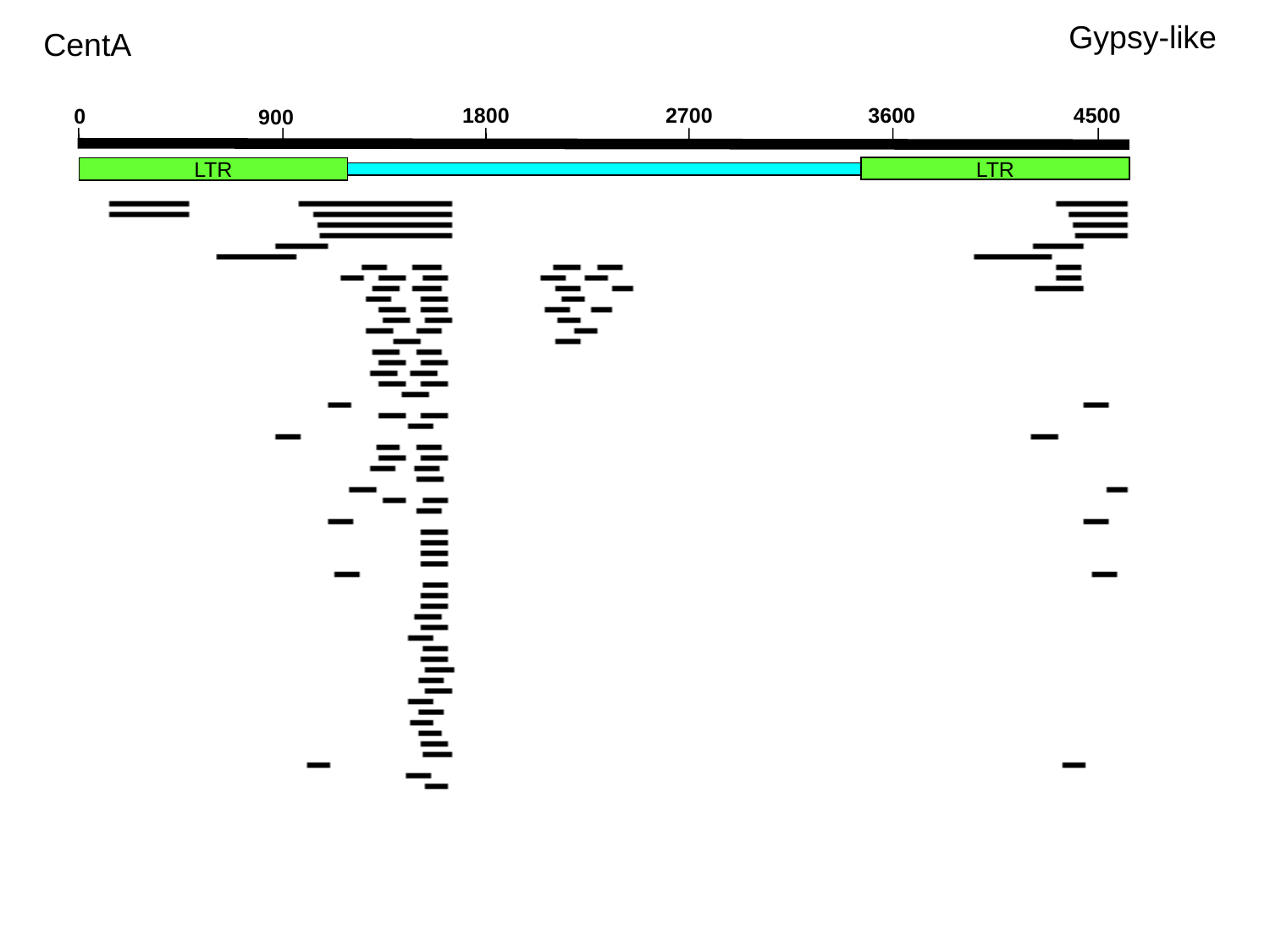

Gypsy-like
CentA
1800
4500
2700
3600
0
900
LTR
LTR

## Slide 37
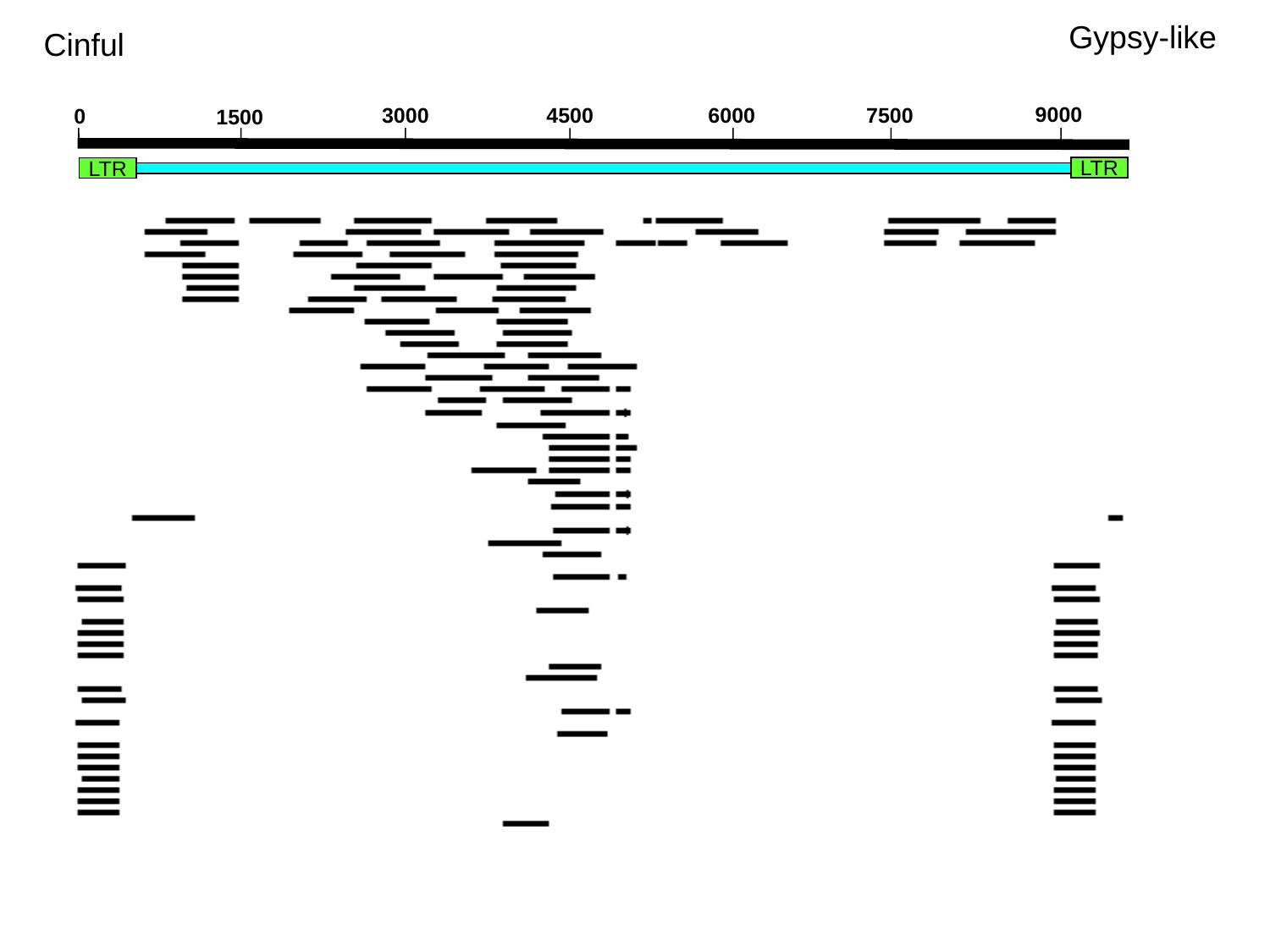

Gypsy-like
Cinful
9000
3000
7500
4500
6000
0
1500
LTR
LTR

## Slide 38
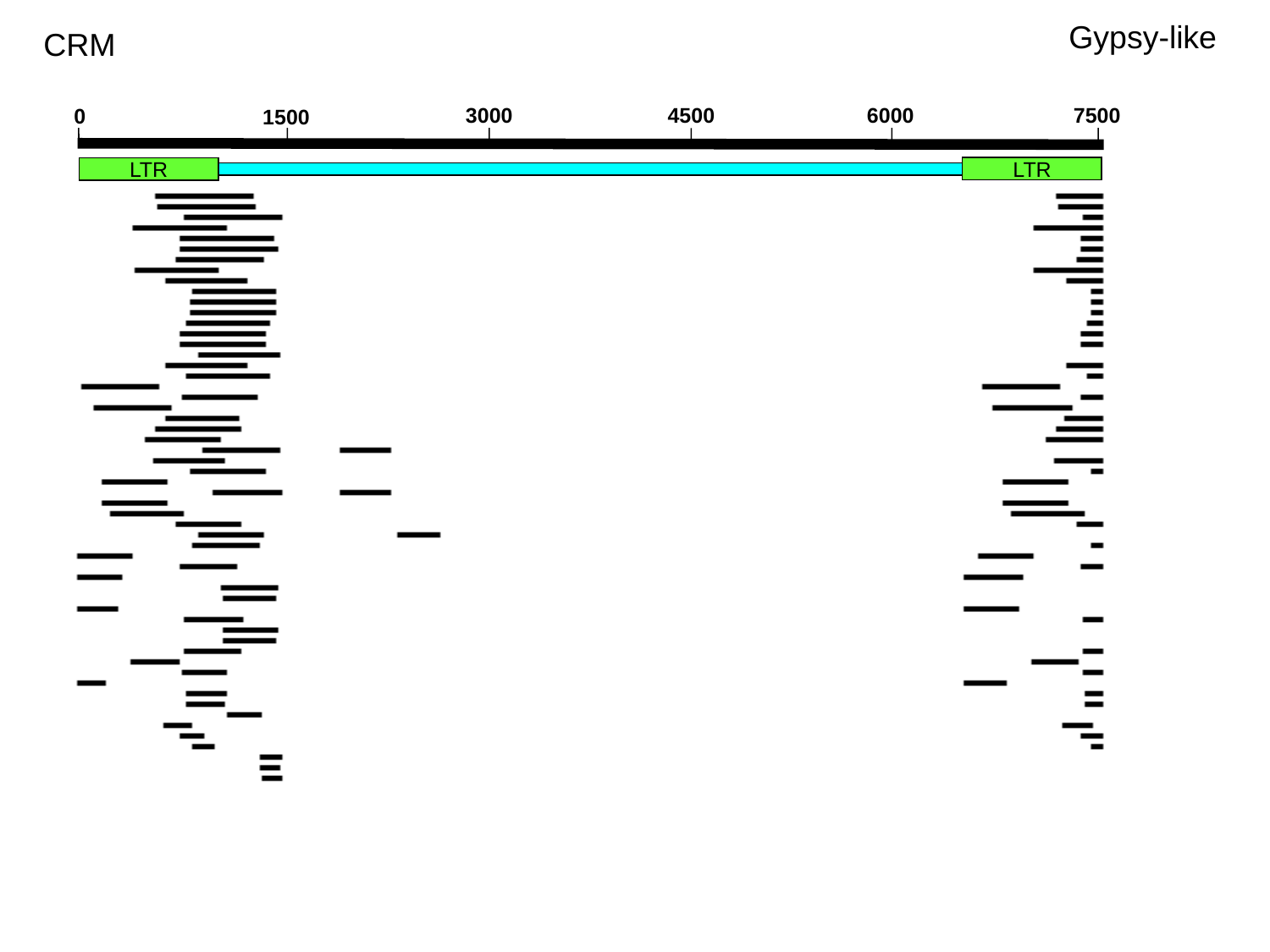

Gypsy-like
CRM
3000
7500
4500
6000
0
1500
LTR
LTR

## Slide 39
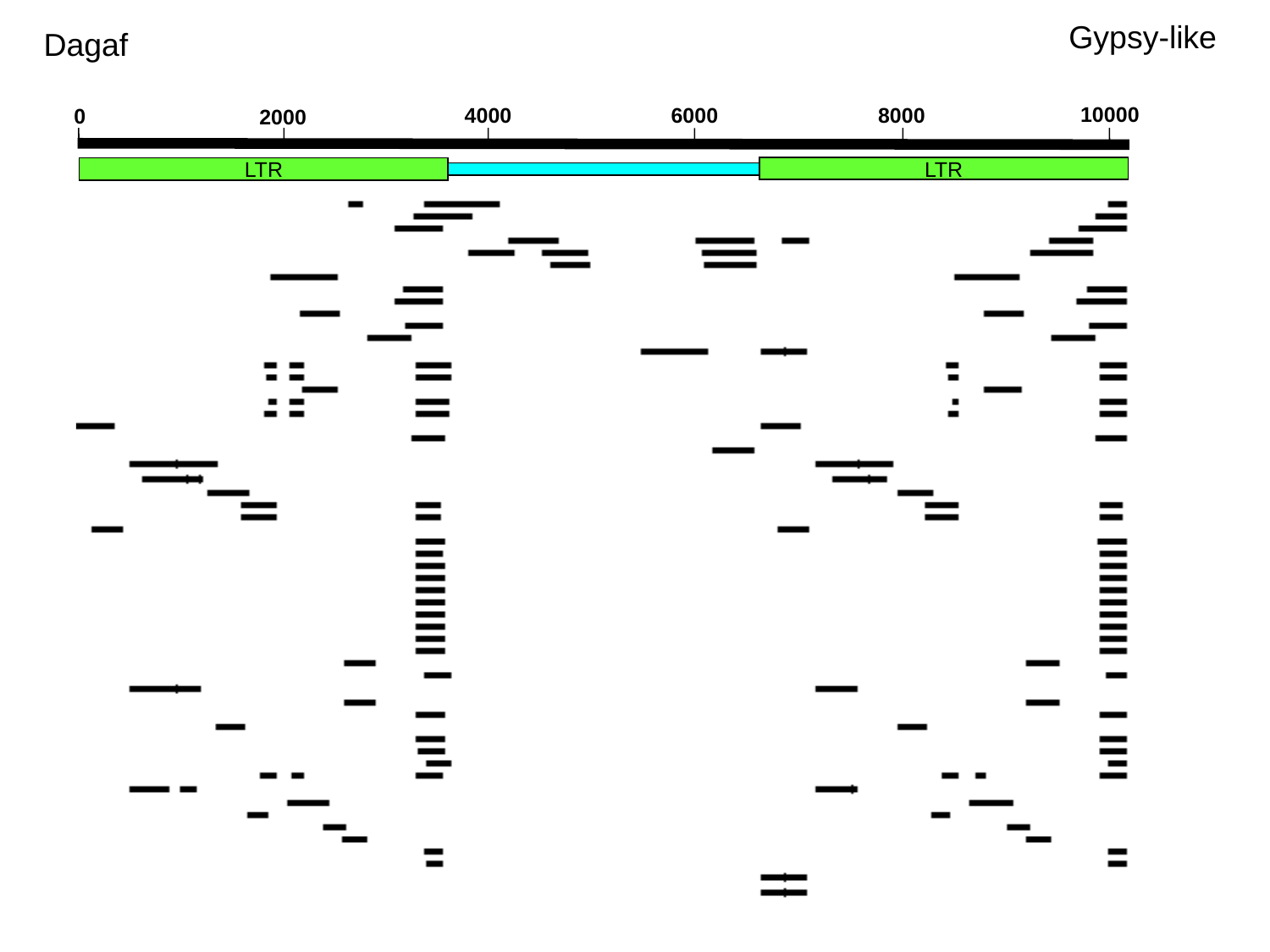

Gypsy-like
Dagaf
10000
4000
6000
8000
0
2000
LTR
LTR

## Slide 40
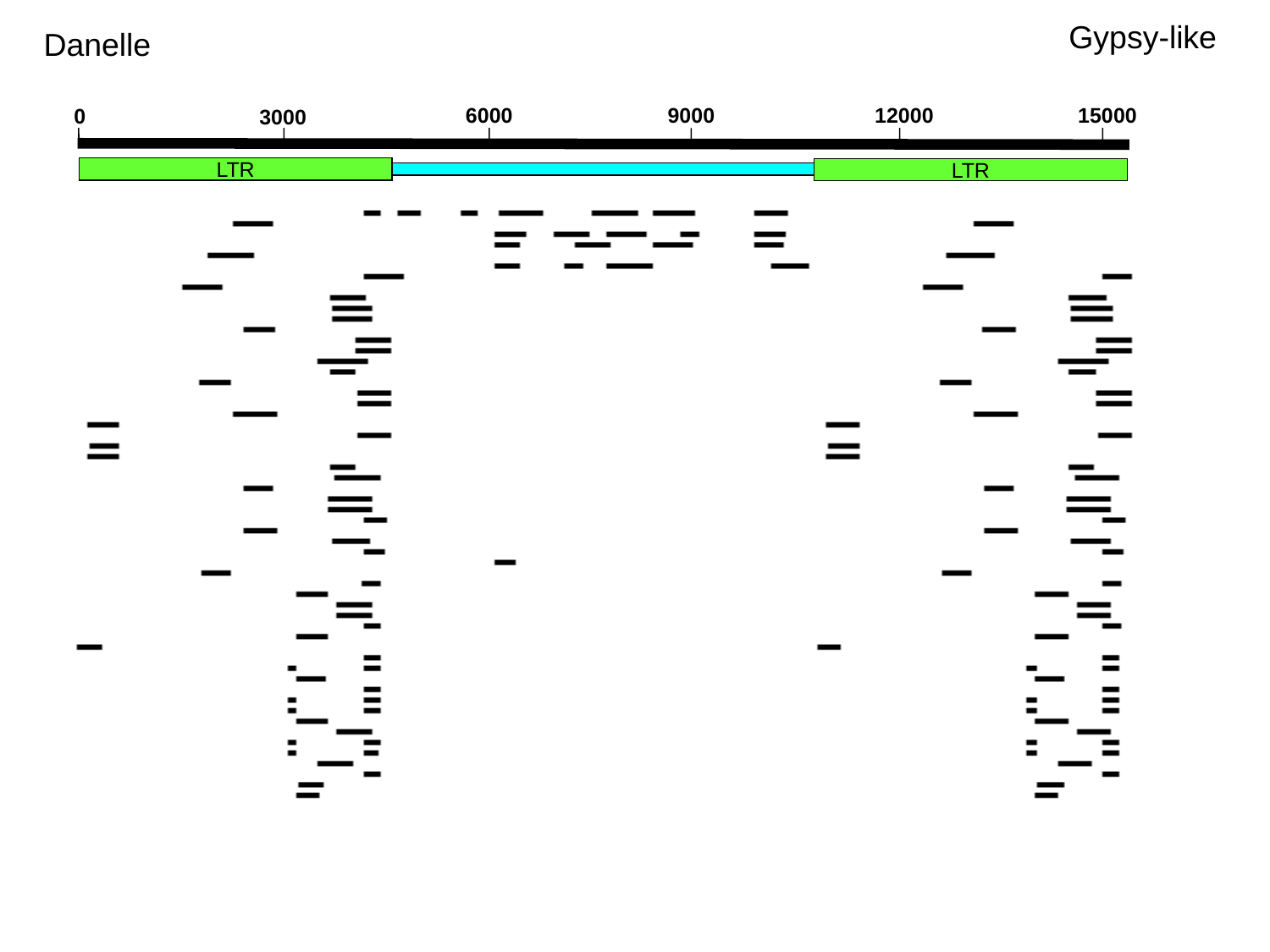

Gypsy-like
Danelle
6000
15000
9000
12000
0
3000
LTR
LTR

## Slide 41
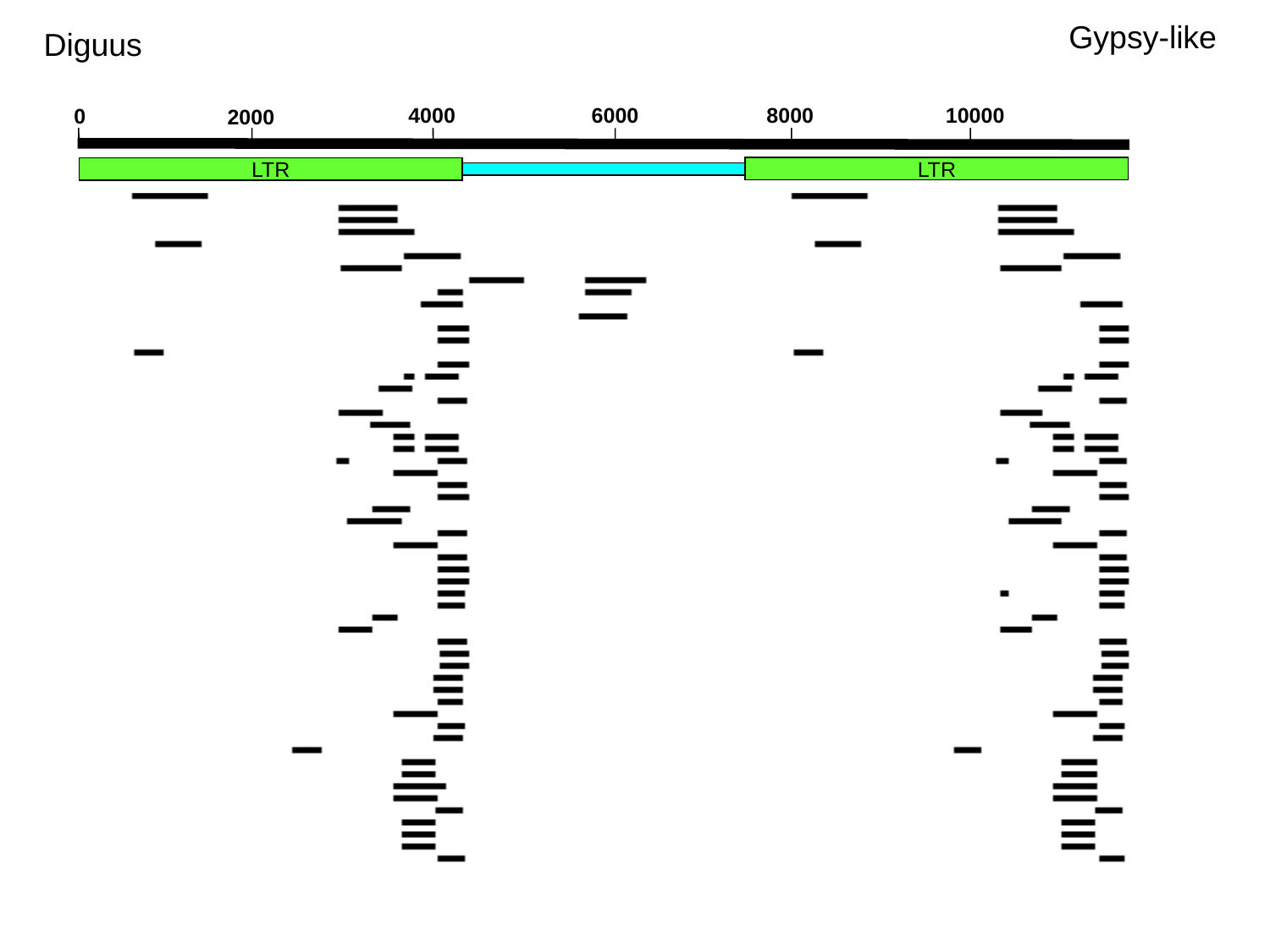

Gypsy-like
Diguus
4000
10000
6000
8000
0
2000
LTR
LTR

## Slide 42
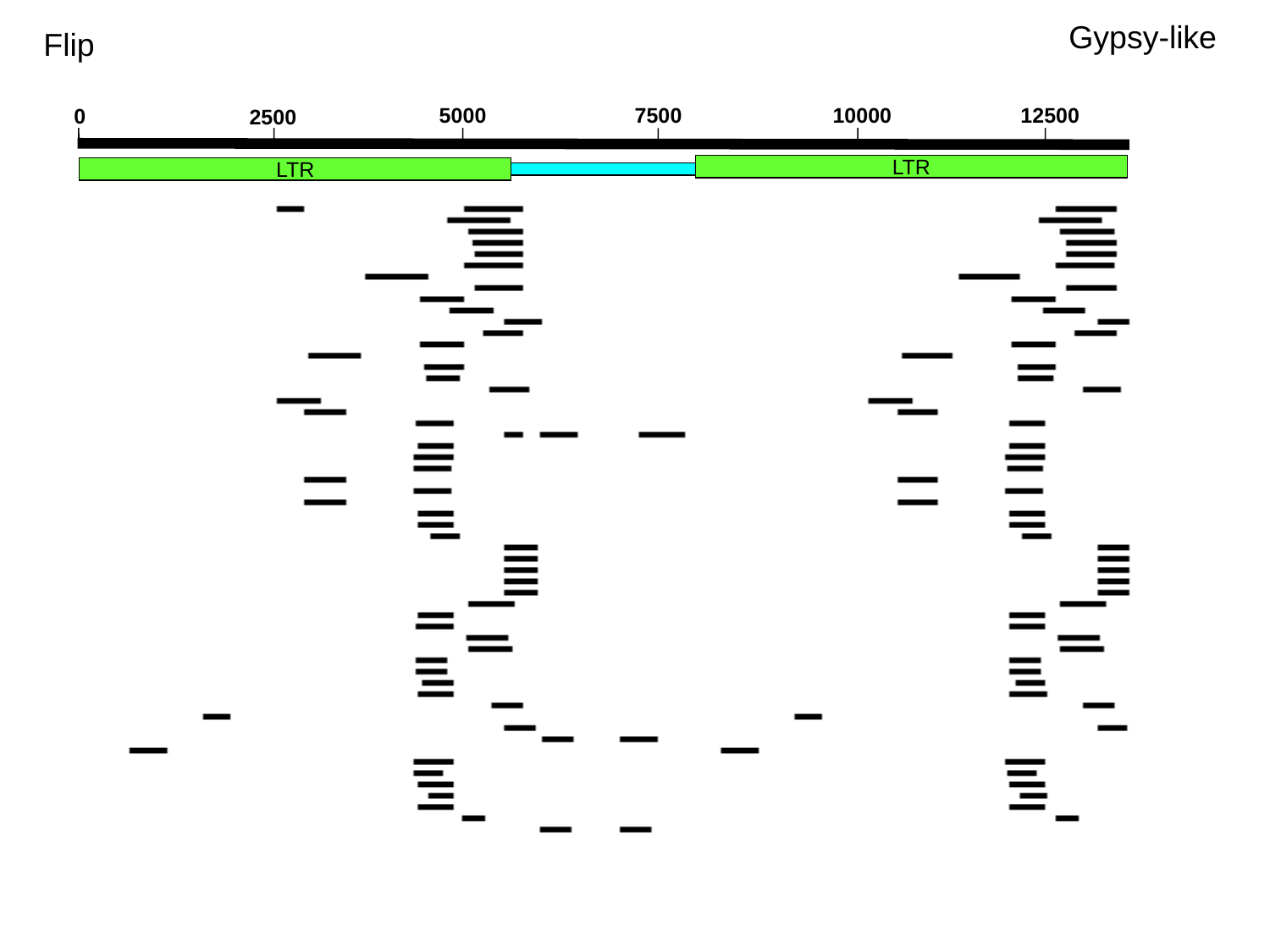

Gypsy-like
Flip
5000
12500
7500
10000
0
2500
LTR
LTR

## Slide 43
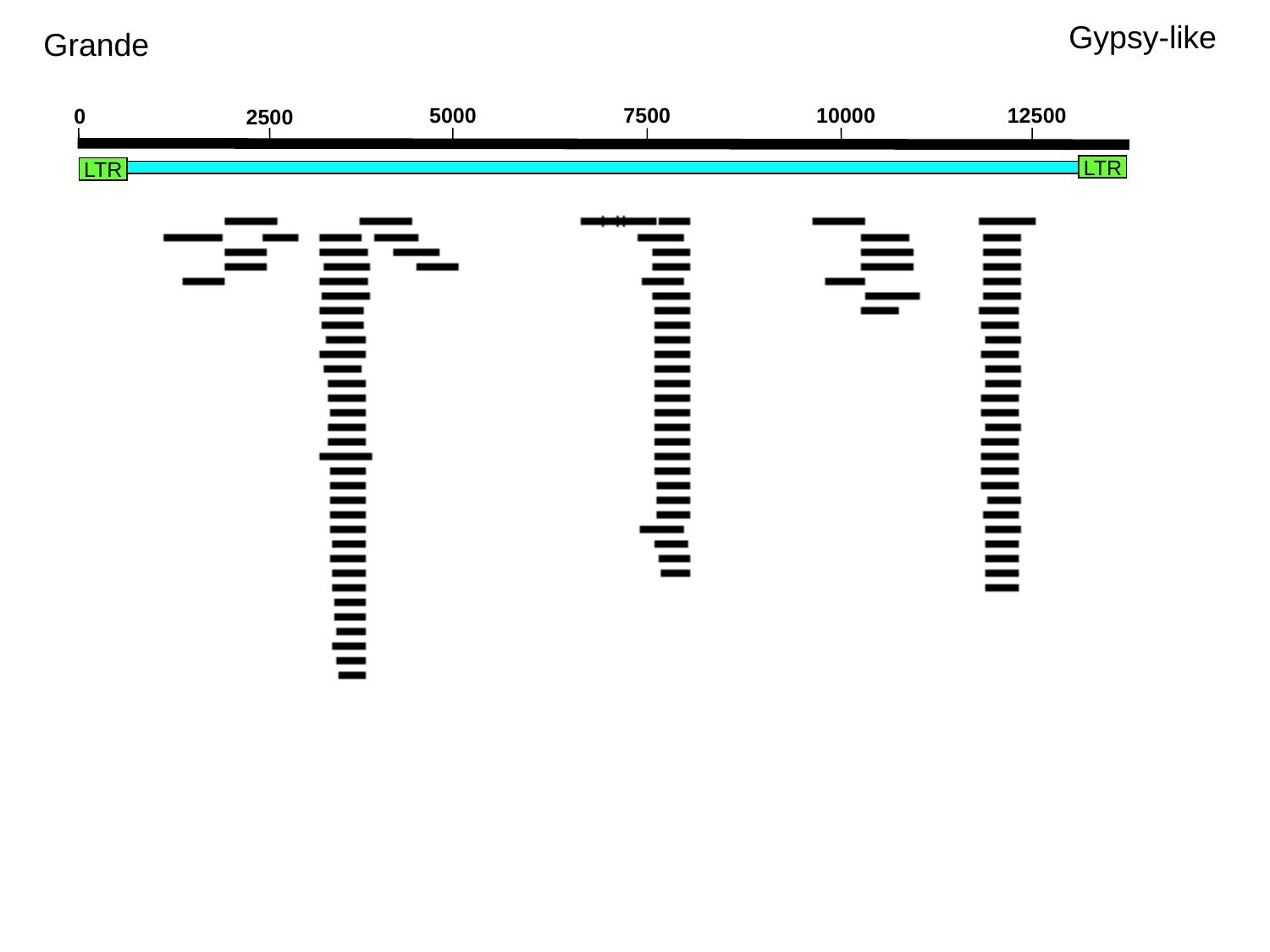

Gypsy-like
Grande
5000
12500
7500
10000
0
2500
LTR
LTR

## Slide 44
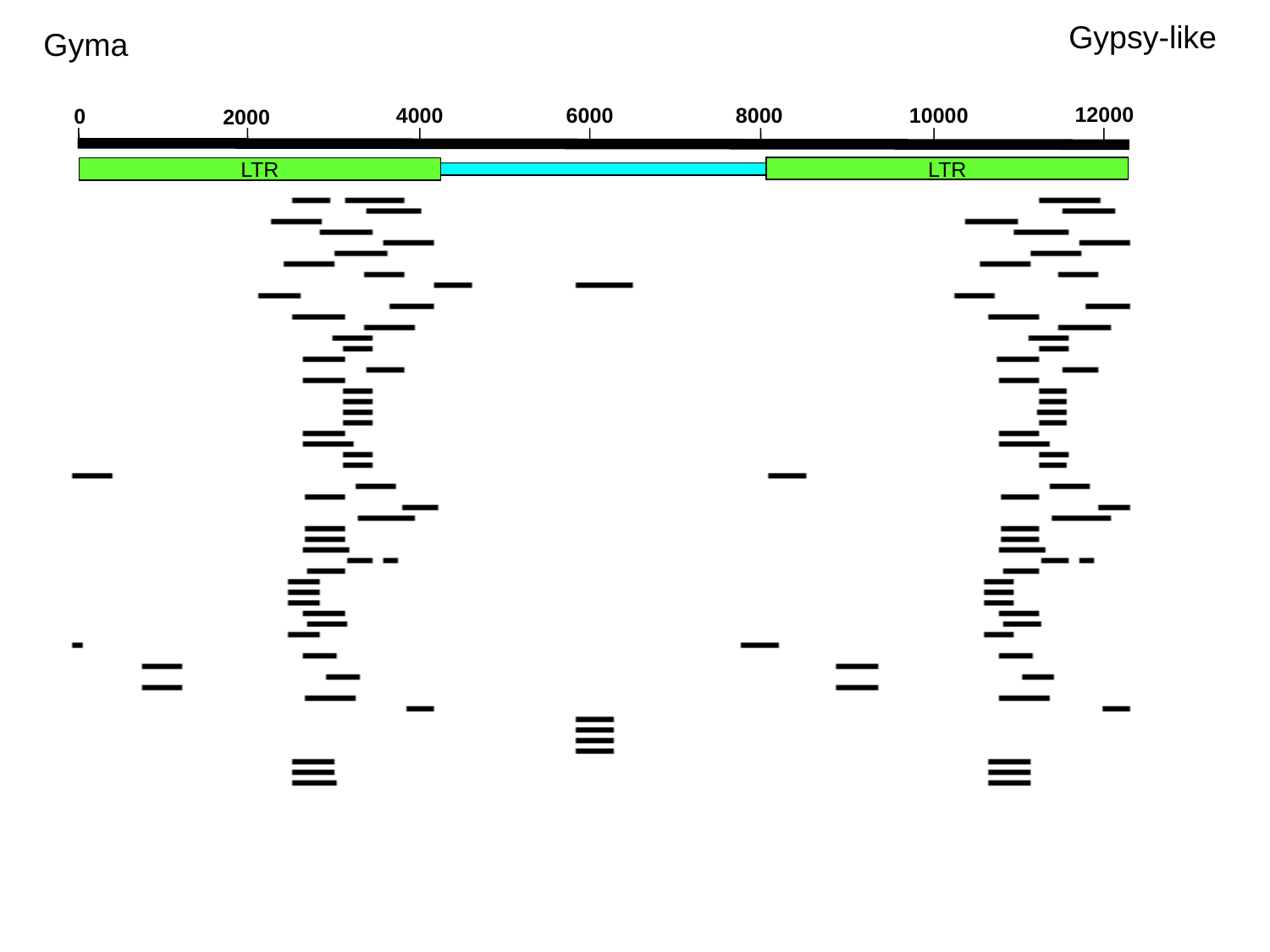

Gypsy-like
Gyma
12000
4000
10000
6000
8000
0
2000
LTR
LTR

## Slide 45
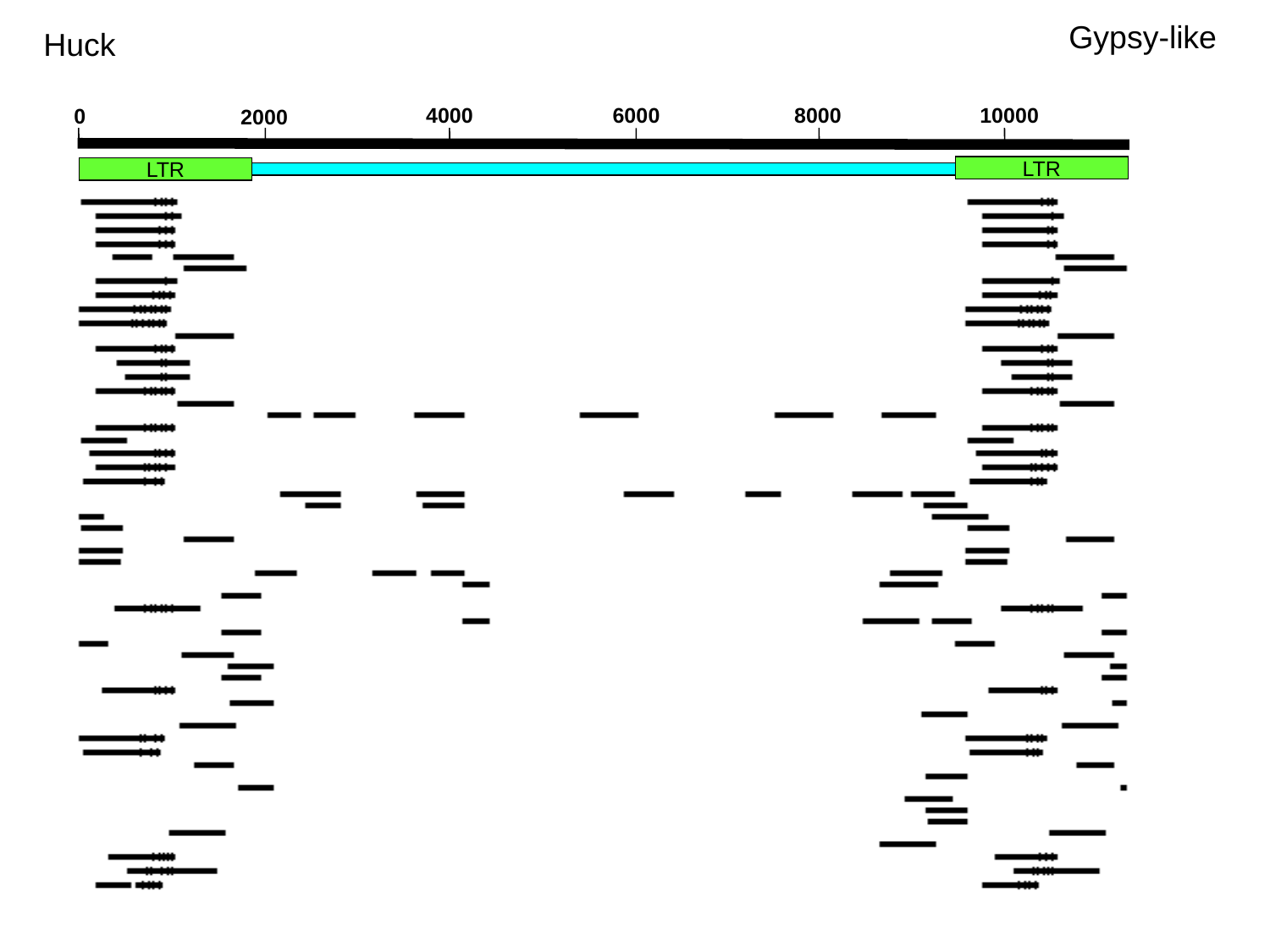

Gypsy-like
Huck
4000
10000
6000
8000
0
2000
LTR
LTR

## Slide 46
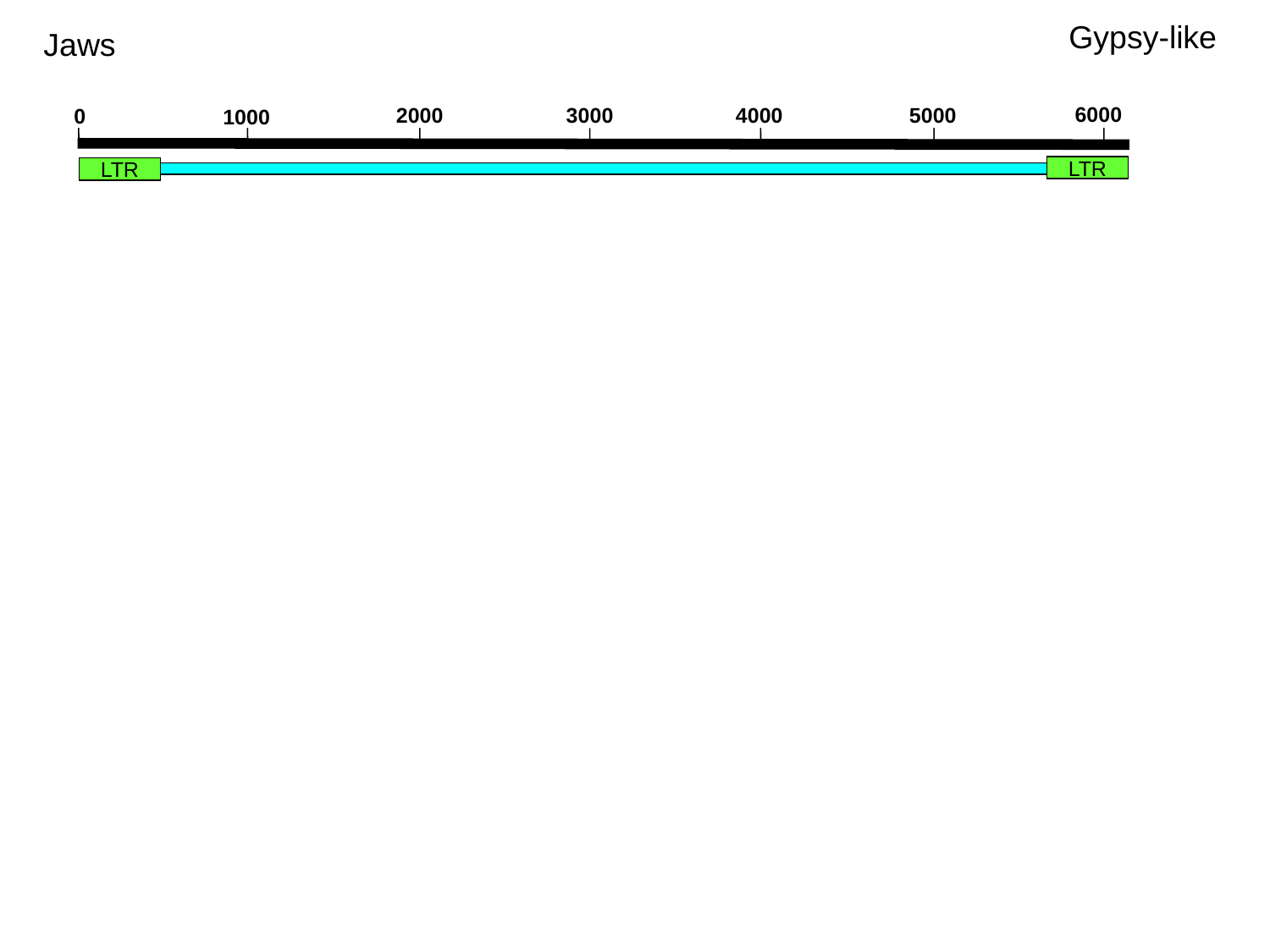

Gypsy-like
Jaws
6000
2000
5000
3000
4000
0
1000
LTR
LTR

## Slide 47
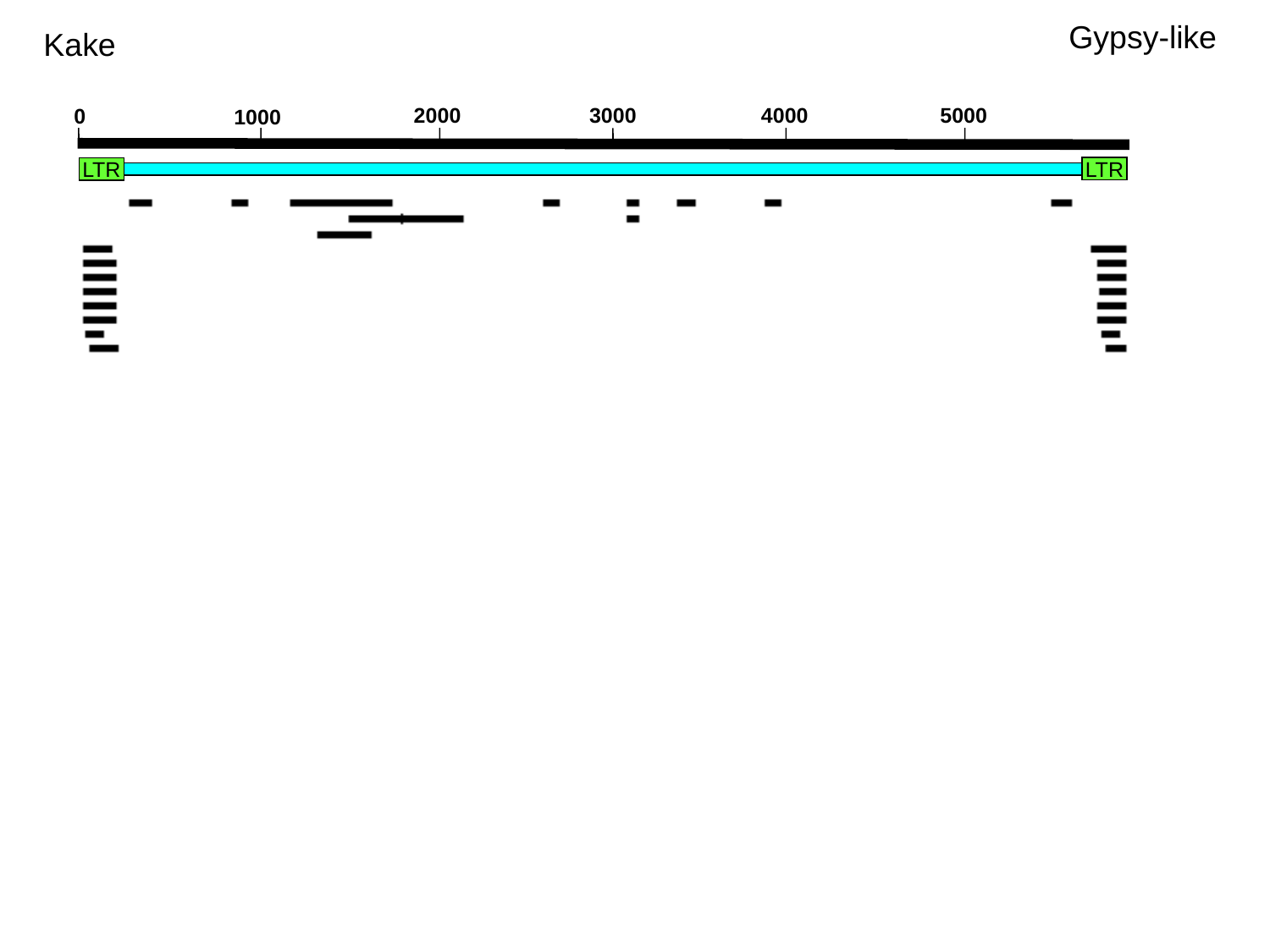

Gypsy-like
Kake
2000
5000
3000
4000
0
1000
LTR
LTR

## Slide 48
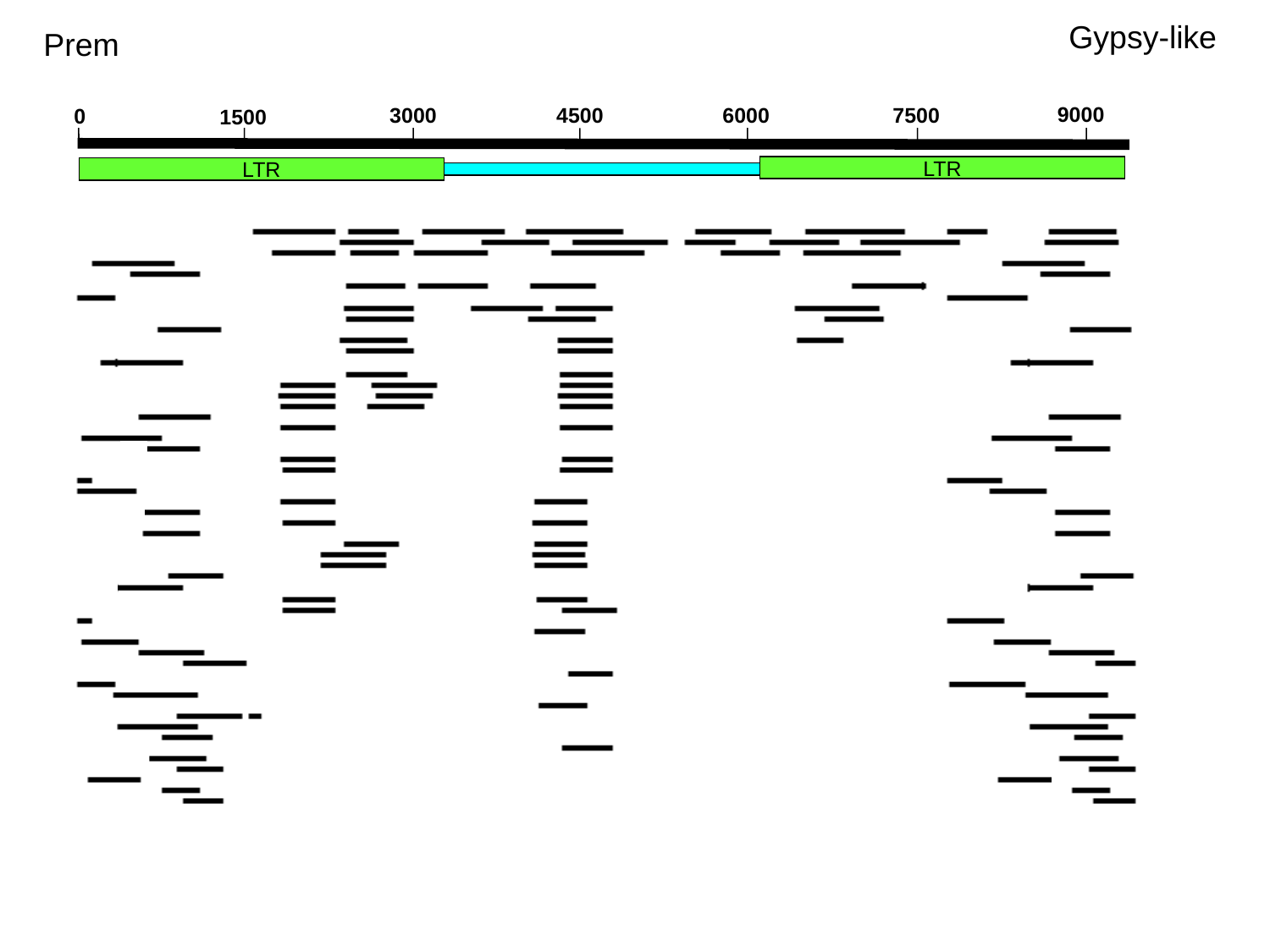

Gypsy-like
Prem
9000
3000
7500
4500
6000
0
1500
LTR
LTR

## Slide 49
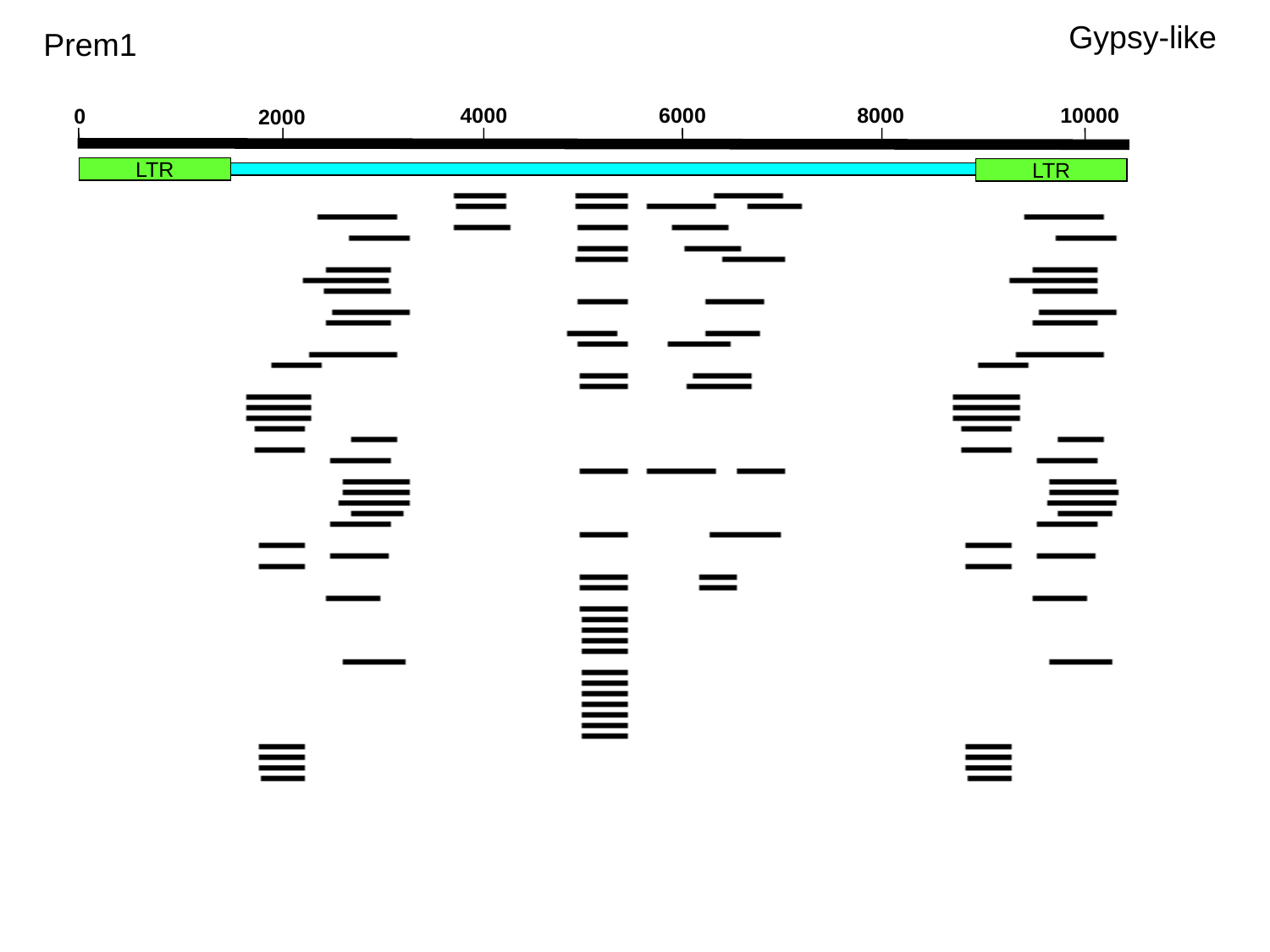

Gypsy-like
Prem1
4000
10000
6000
8000
0
2000
LTR
LTR

## Slide 50
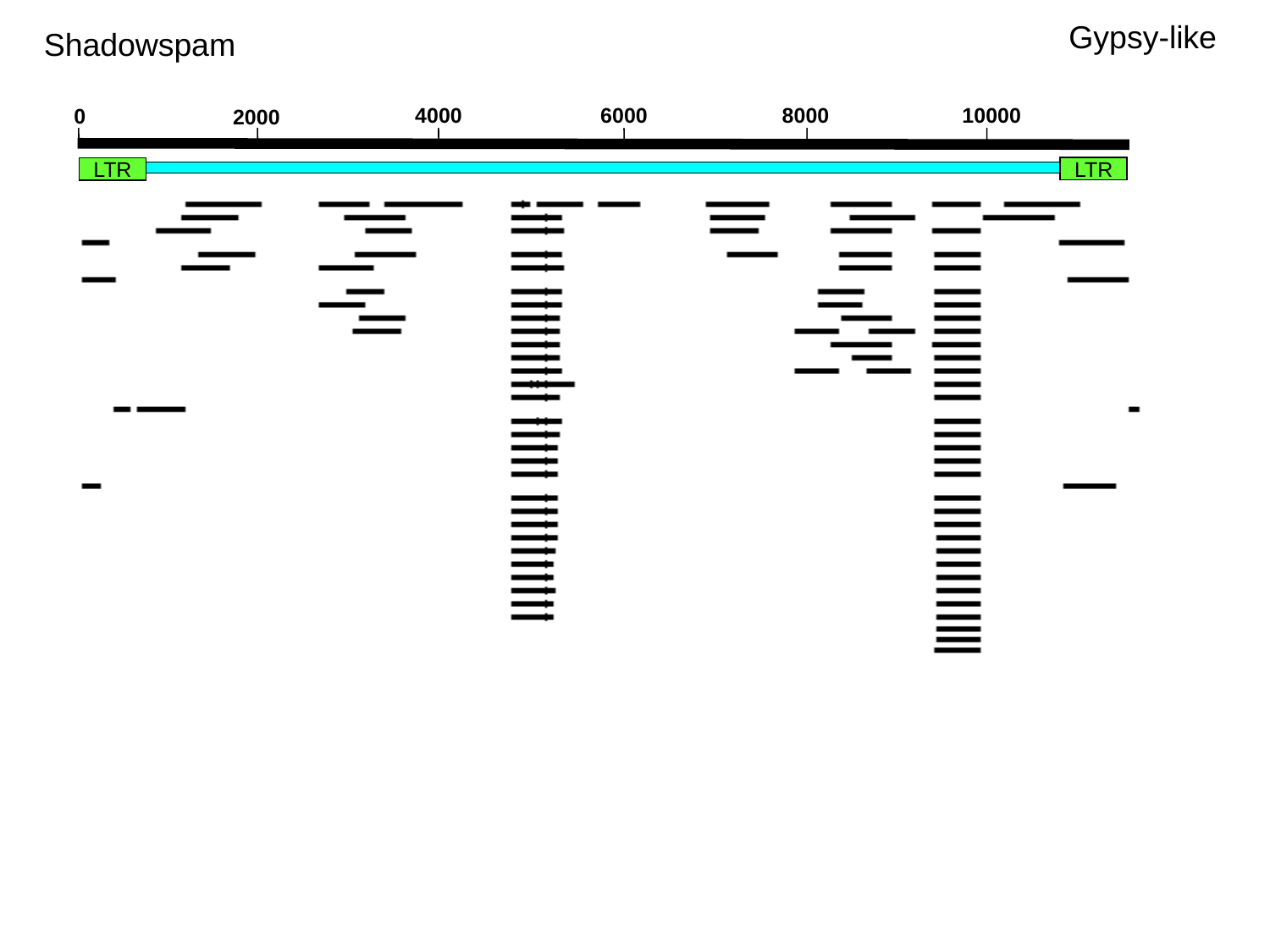

Gypsy-like
Shadowspam
4000
10000
6000
8000
0
2000
LTR
LTR

## Slide 51
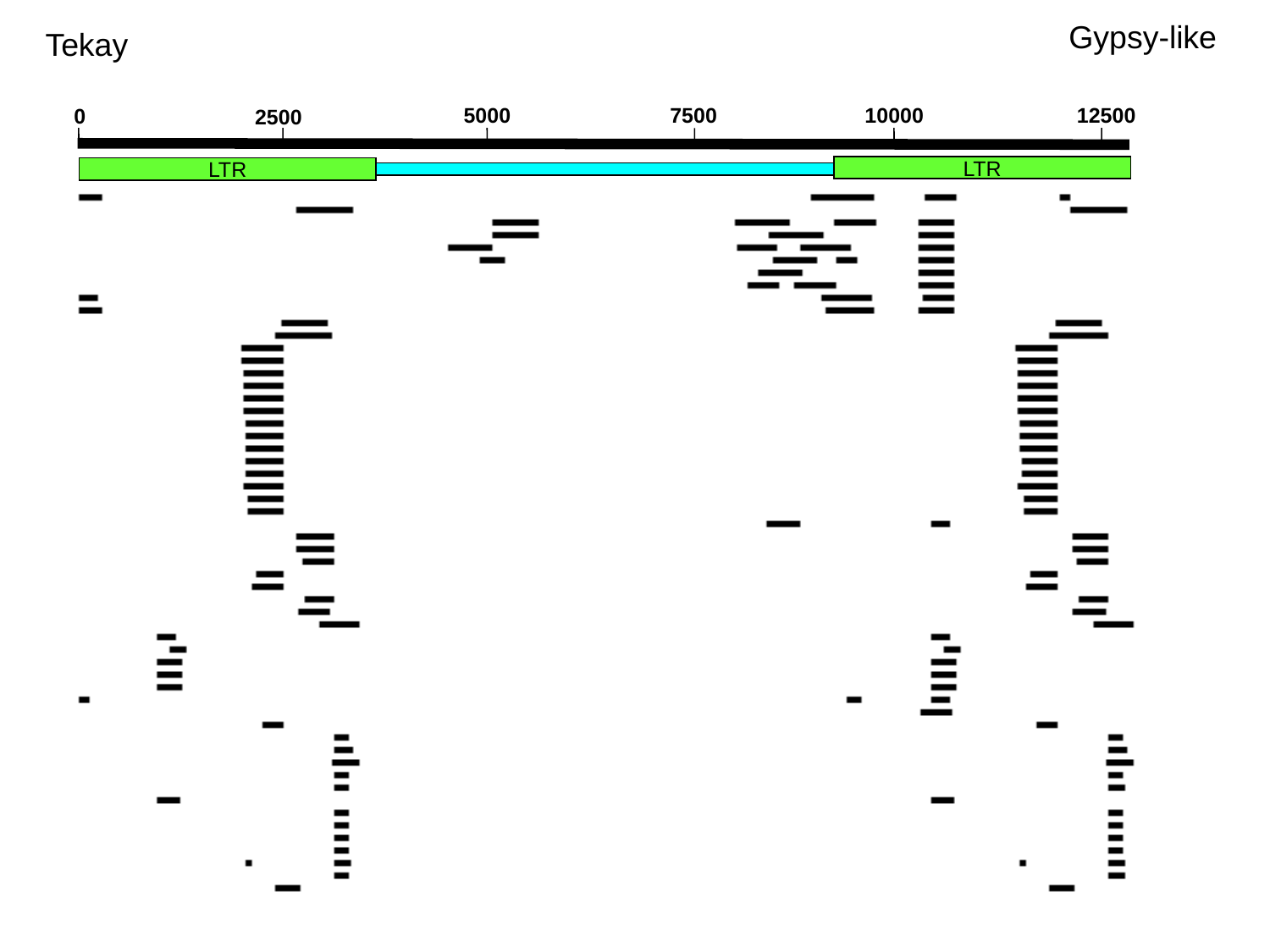

Gypsy-like
Tekay
5000
12500
7500
10000
0
2500
LTR
LTR

## Slide 52
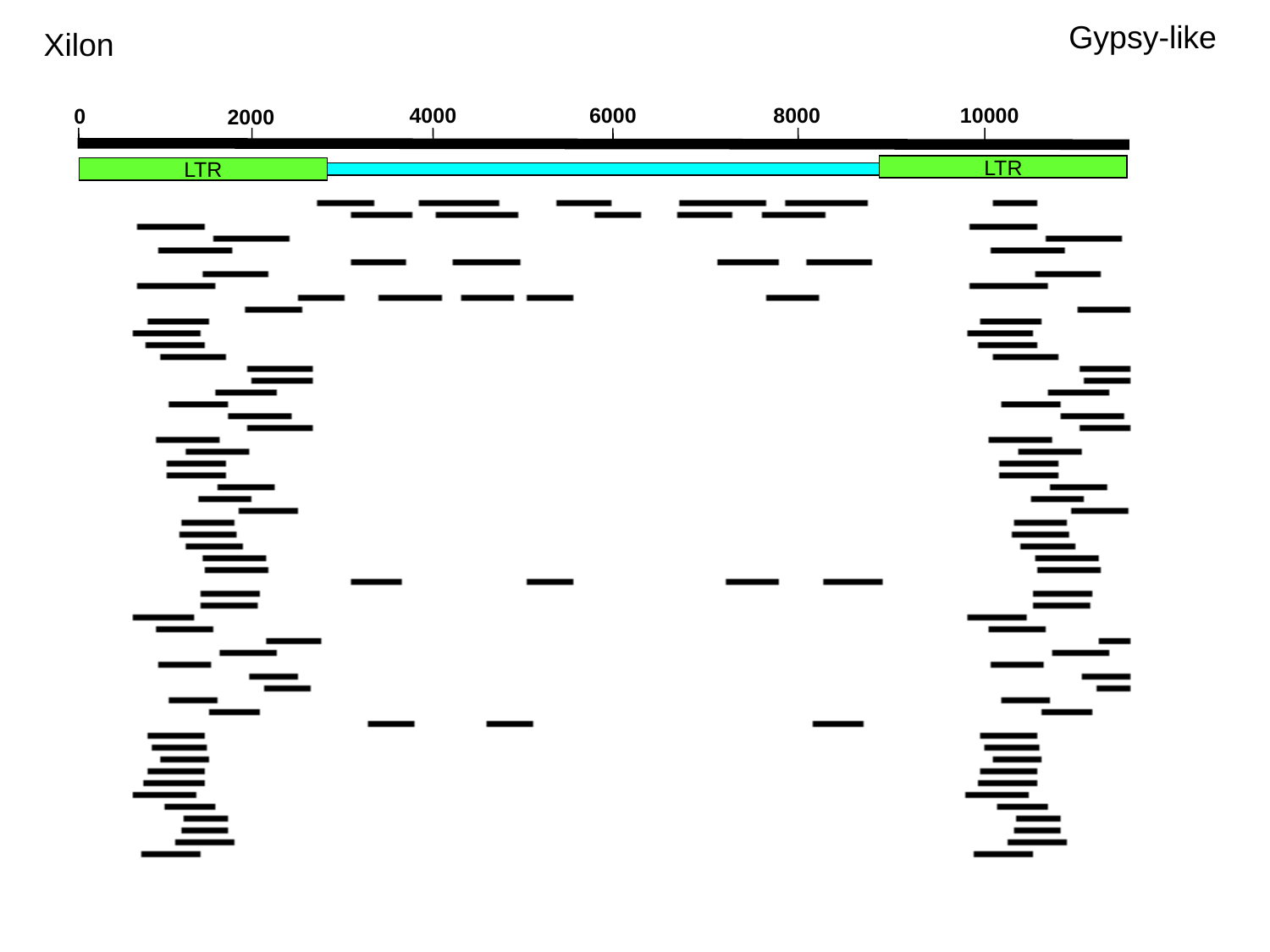

Gypsy-like
Xilon
4000
10000
6000
8000
0
2000
LTR
LTR

## Slide 53
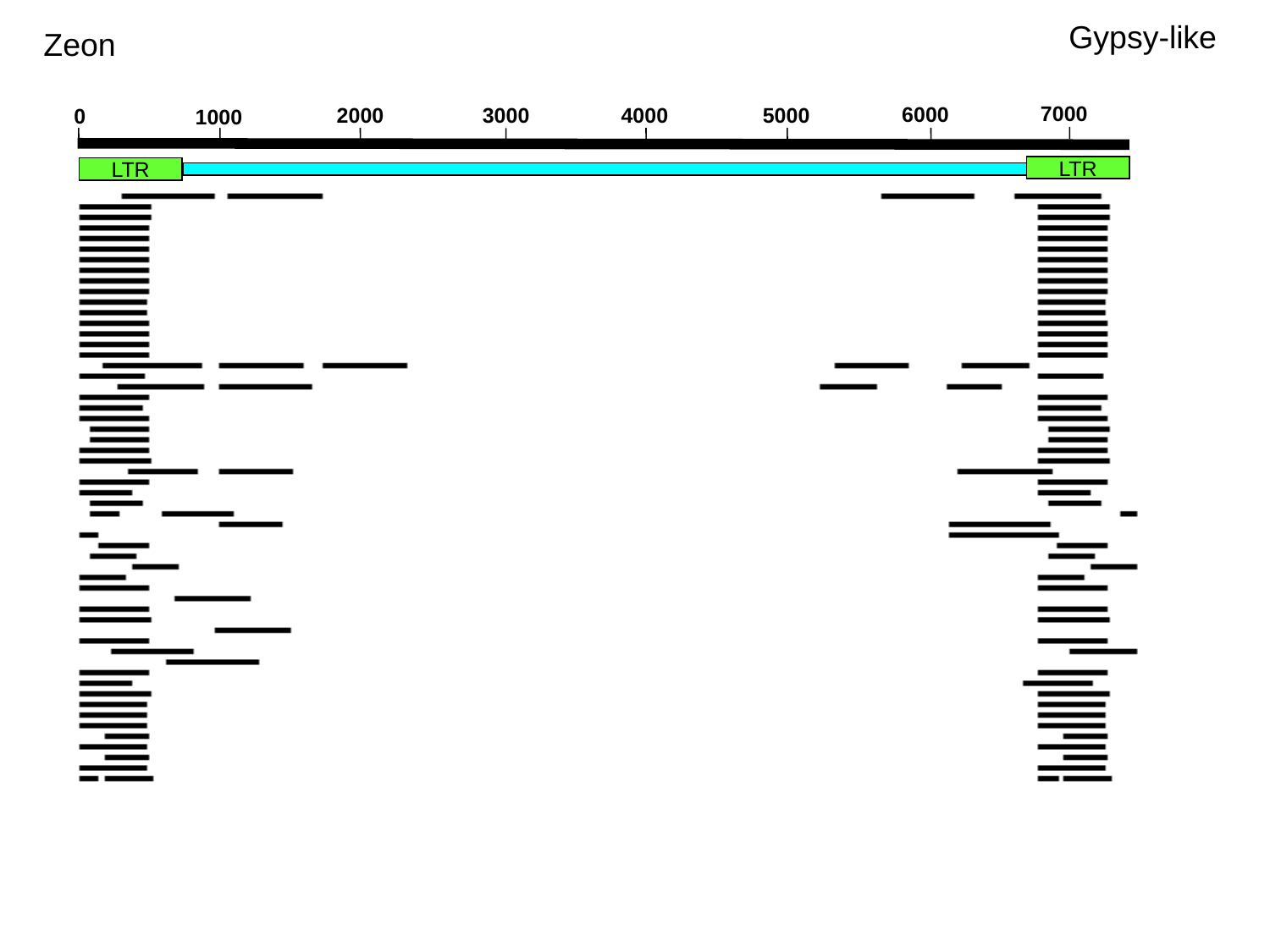

Gypsy-like
Zeon
7000
6000
2000
5000
3000
4000
0
1000
LTR
LTR

## Slide 54
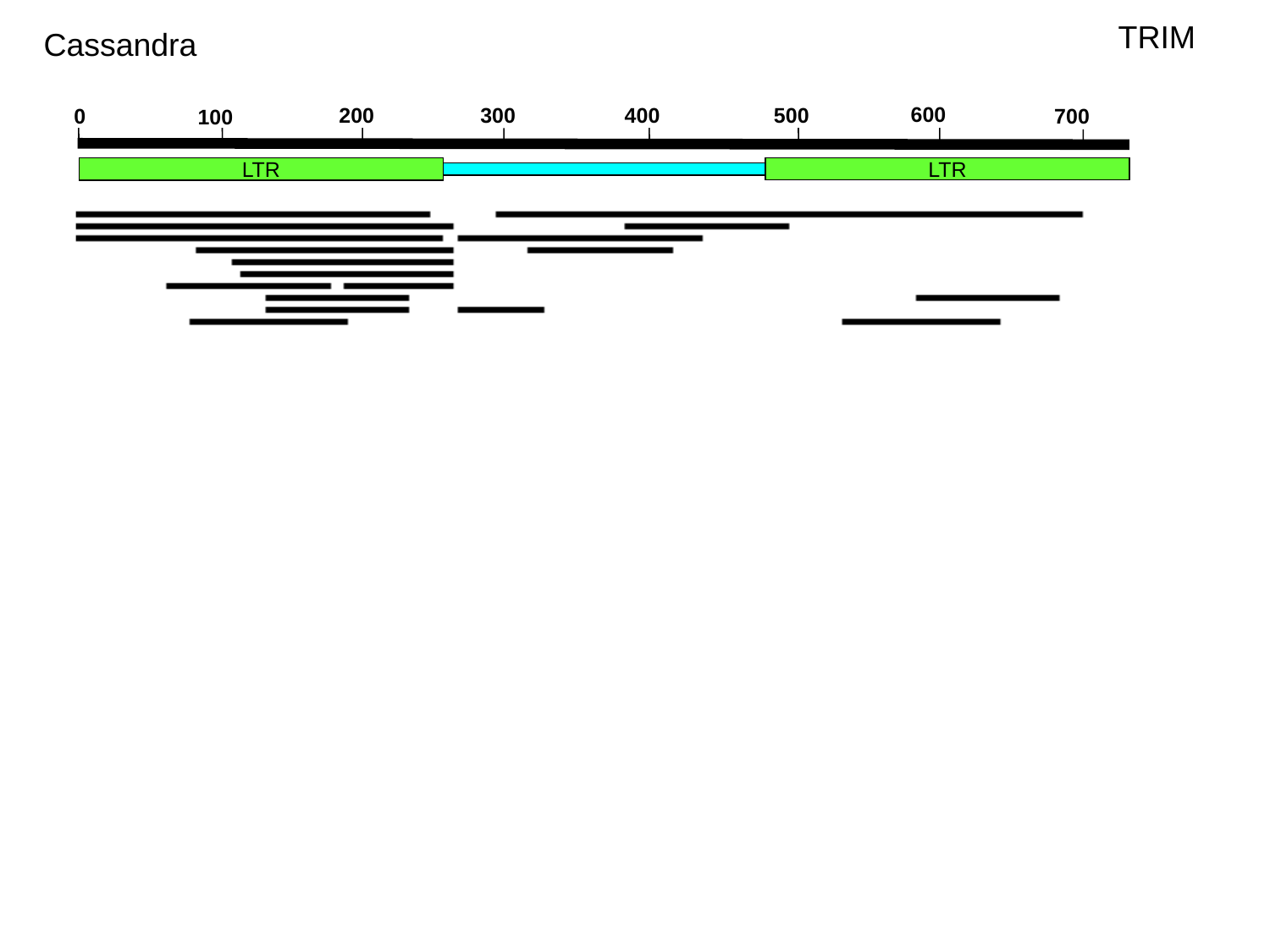

TRIM
Cassandra
600
200
500
300
400
0
700
100
LTR
LTR

## Slide 55
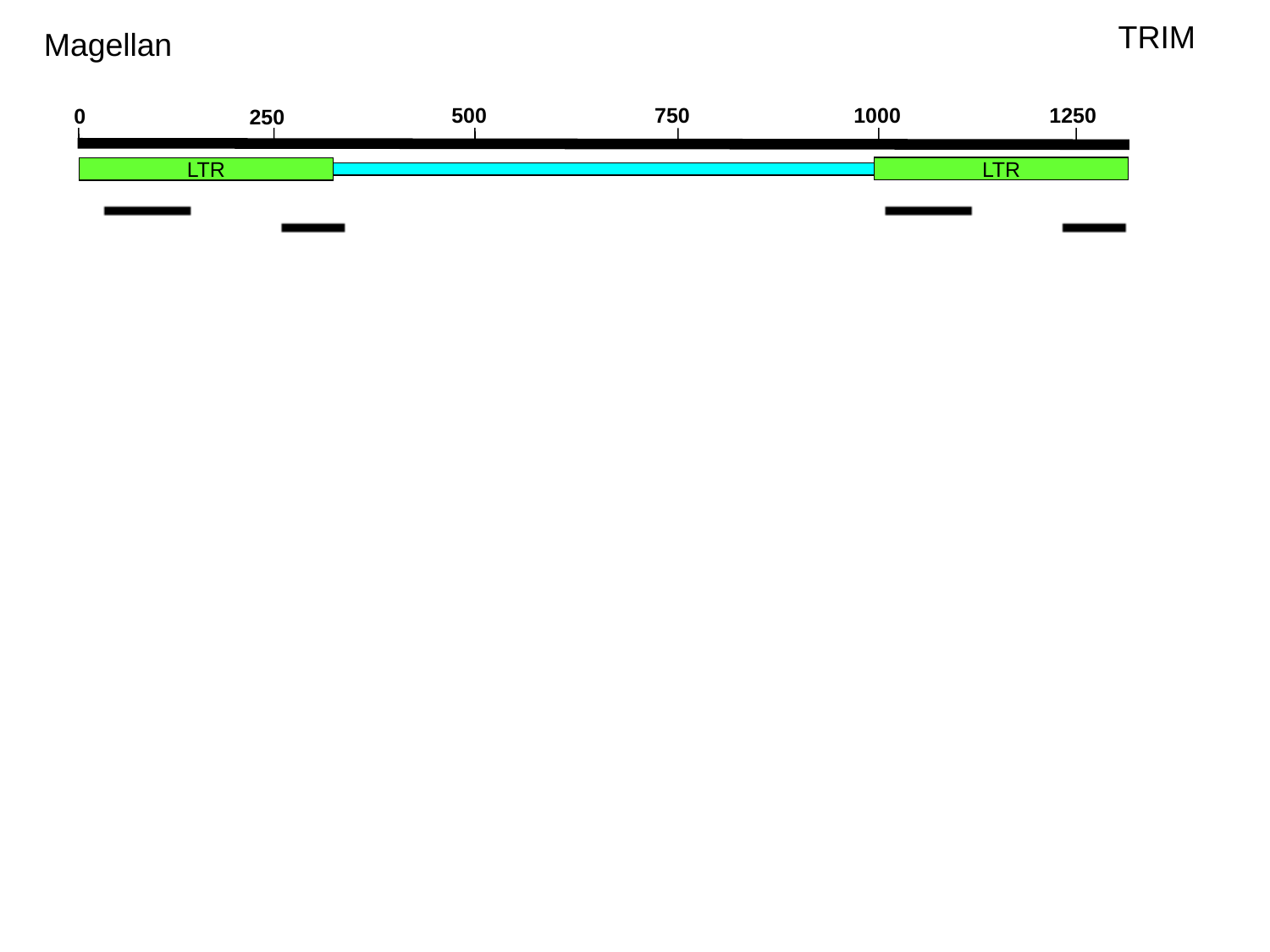

TRIM
Magellan
500
1250
750
1000
0
250
LTR
LTR

## Slide 56
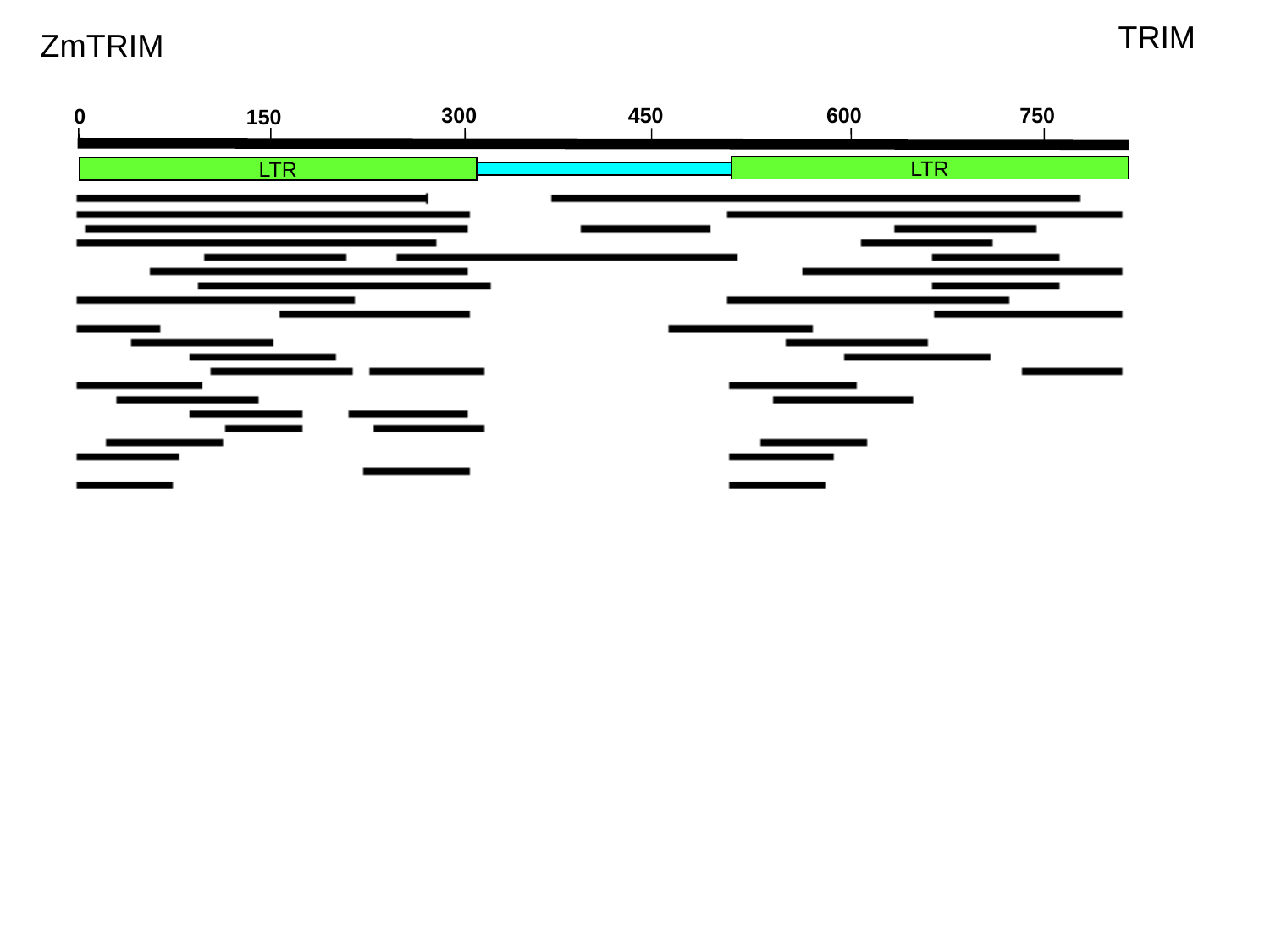

TRIM
ZmTRIM
300
750
450
600
0
150
LTR
LTR

## Slide 57
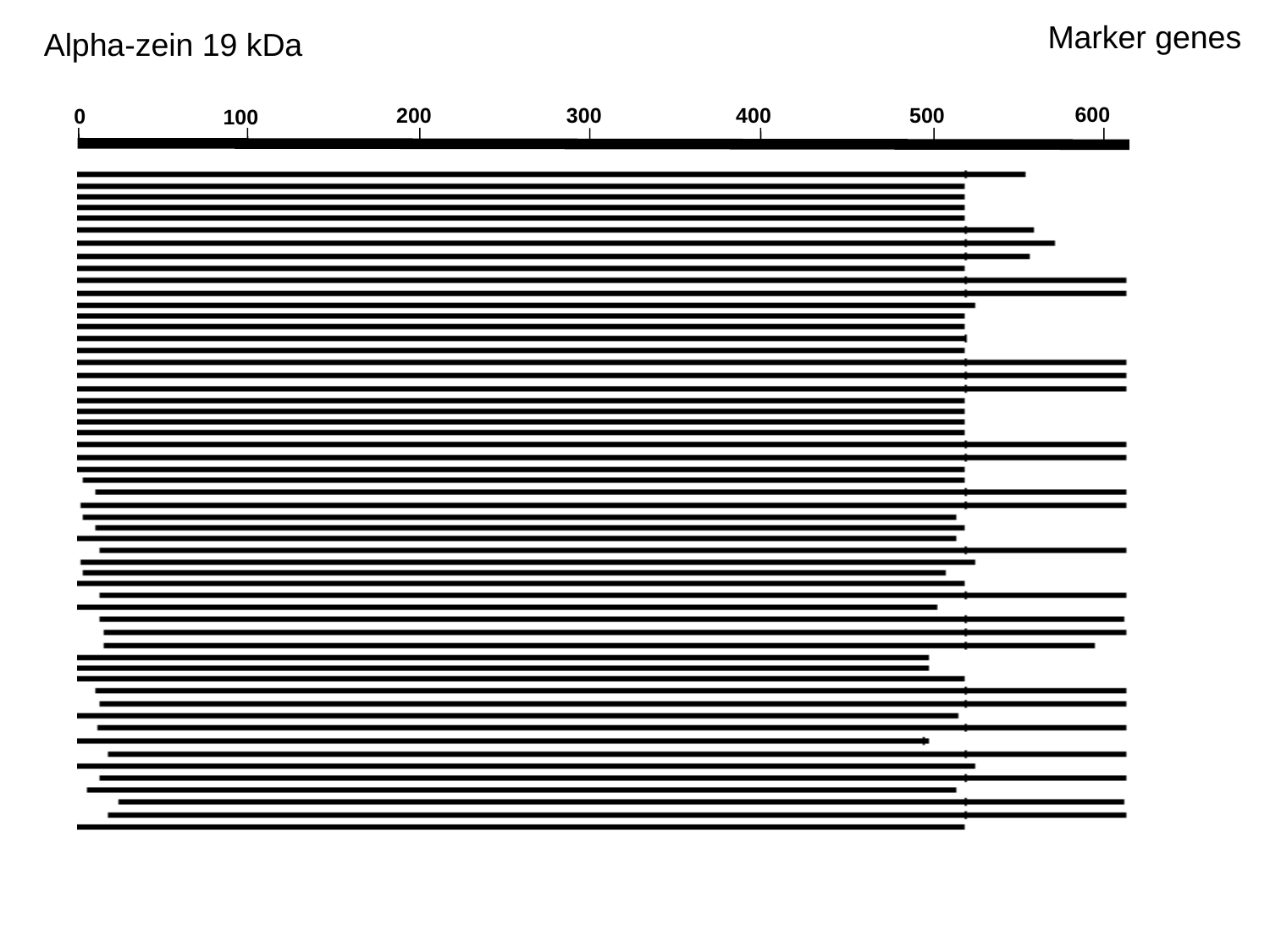

Marker genes
Alpha-zein 19 kDa
600
200
500
300
400
0
100

## Slide 58
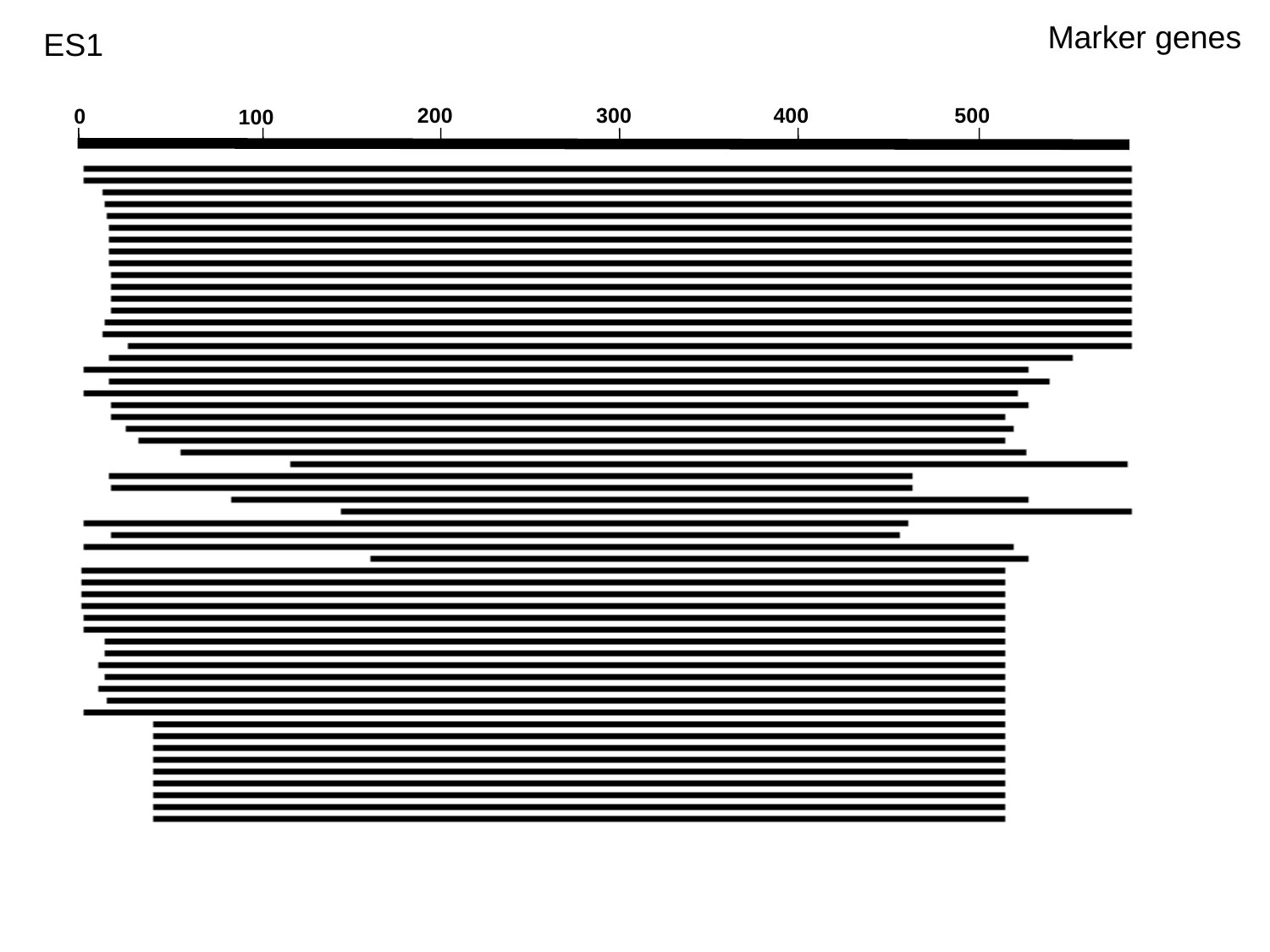

Marker genes
ES1
200
500
300
400
0
100

## Slide 59
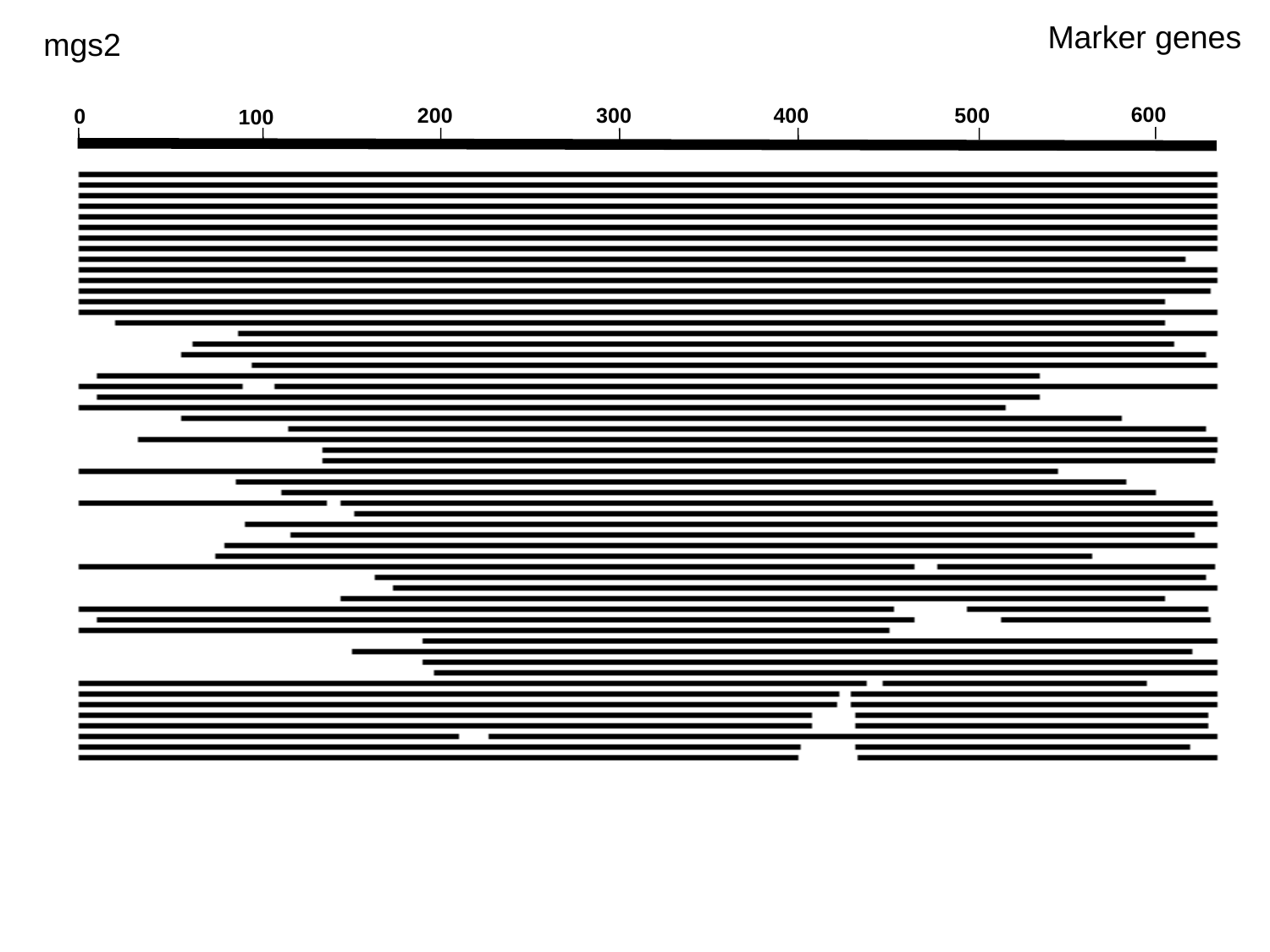

Marker genes
mgs2
600
200
500
300
400
0
100

## Slide 60
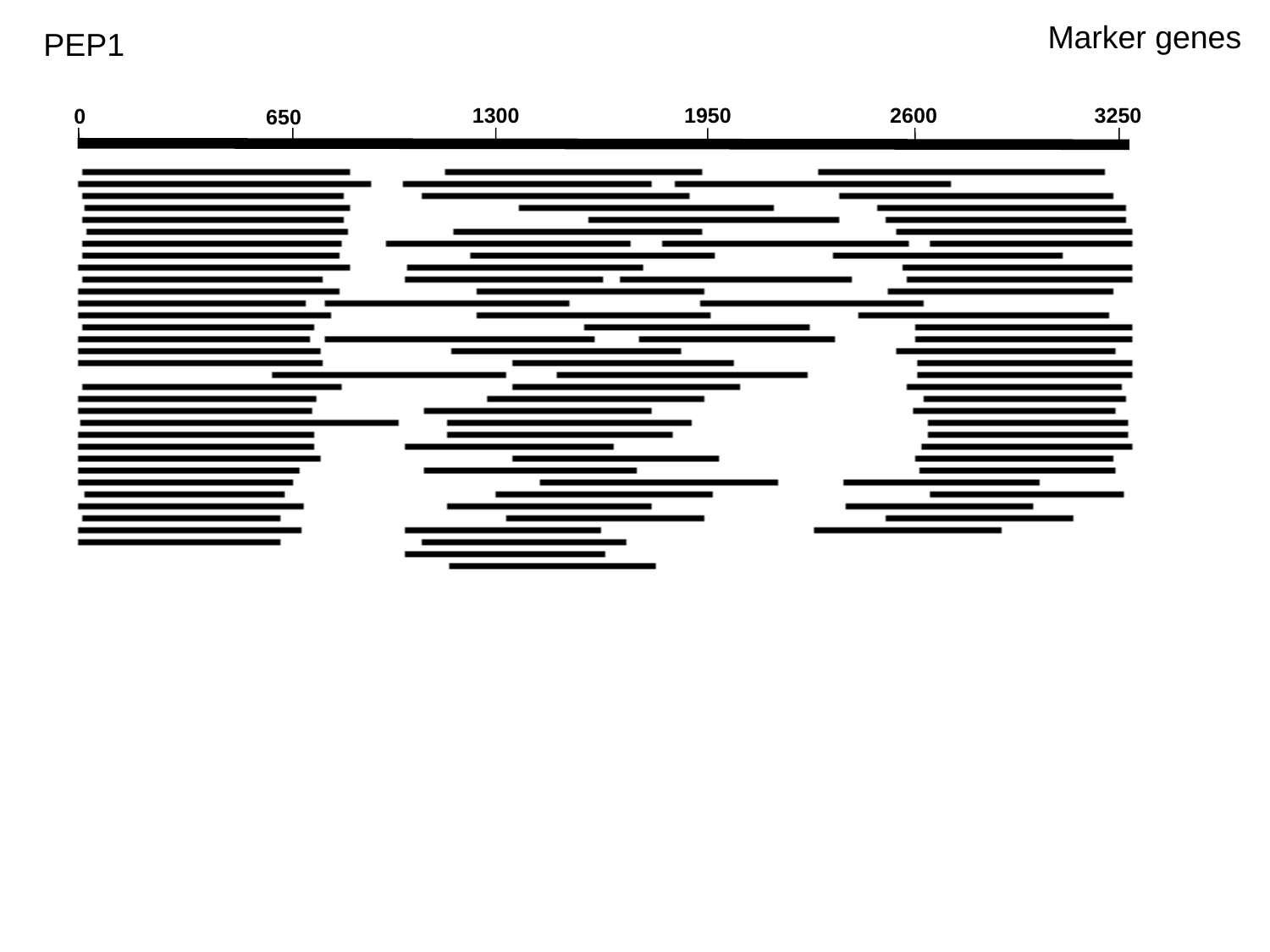

Marker genes
PEP1
1300
3250
1950
2600
0
650
