# Supplementary material for: Transcriptional activity of transposable elements in maize
Source: BMC Genomics. 2010 Oct 25;11:601. doi: 10.1186/1471-2164-11-601 (PMC3091746; doi:10.1186/1471-2164-11-601)
Supplement: Additional file 3 — Phylogenetic analysis of TE-ESTs and genomic sequences of different transposable elements. Phylogenetic analyses were performed using the neighbor-joining algorithm from distance matrices according to Kimura's two-parameter method. Randomly selected genomic sequences are indicated by the accession number of the sequence from which were obtained. ESTs are indicated by their accession numbers. The position of the ESTs is also indicated by circles. The colours of the circles indicate the organ from which the cDNA library was constructed. [file 1471-2164-11-601-S3.PPT]

## Slide 1
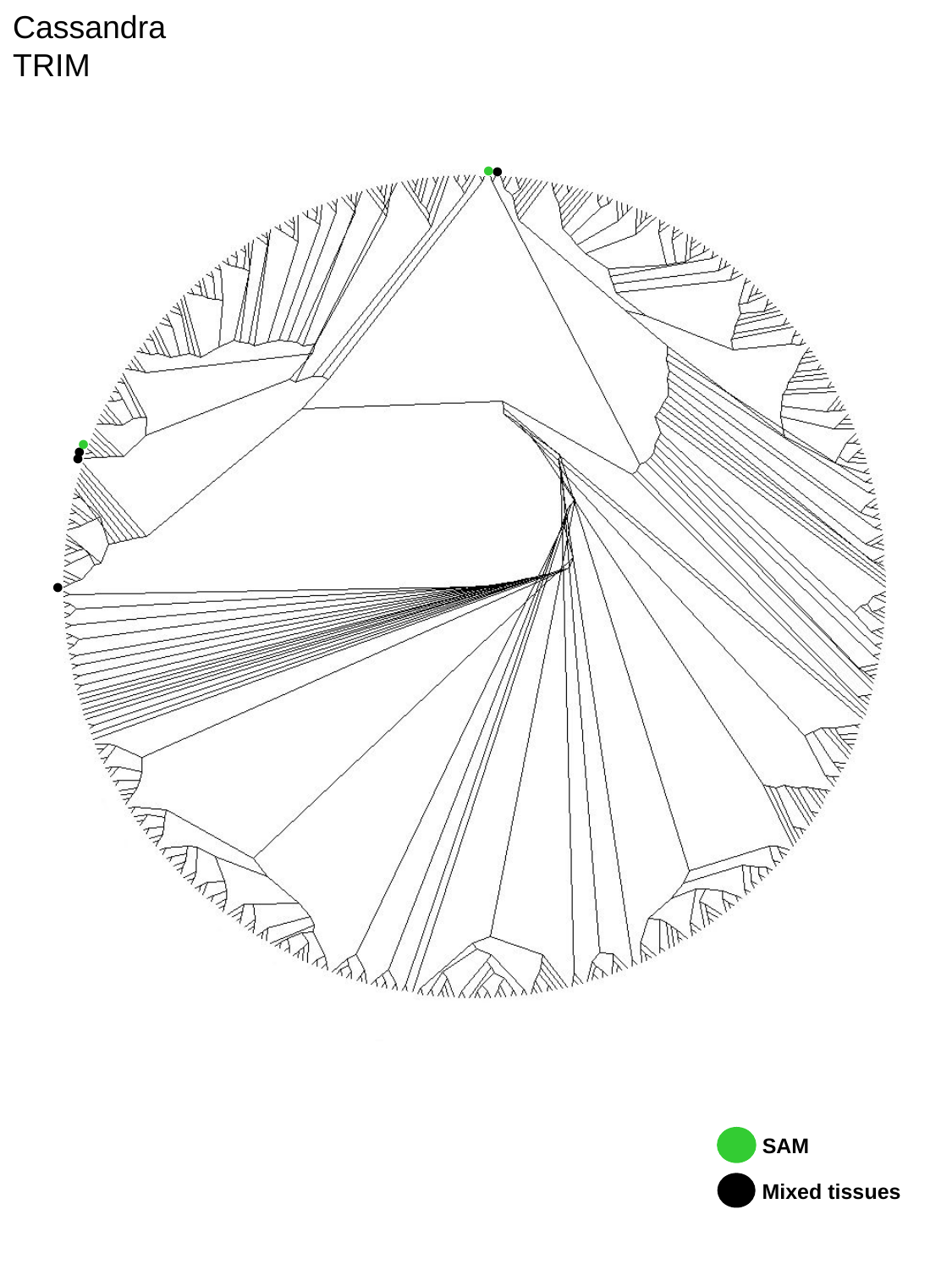

Cassandra
TRIM
●
●
●
●
●
●
SAM
7
Mixed tissues
5

## Slide 2
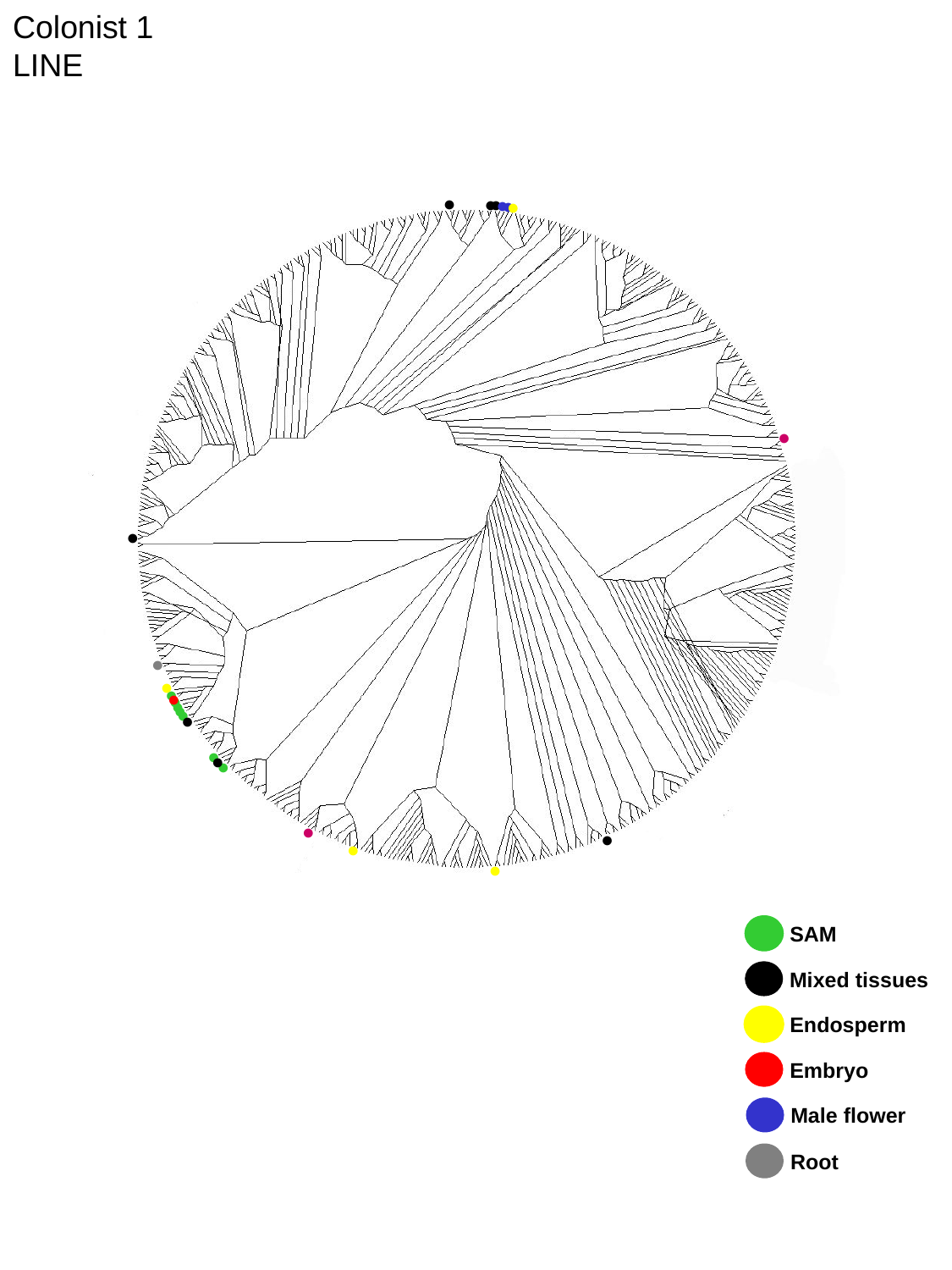

Colonist 1
LINE
●
●
●
●
●
●
●
●
●
●
●
●
●
●
●
●
●
●
●
●
●
●
●
●
SAM
7
Mixed tissues
5
Endosperm
Embryo
Male flower
Root

## Slide 3
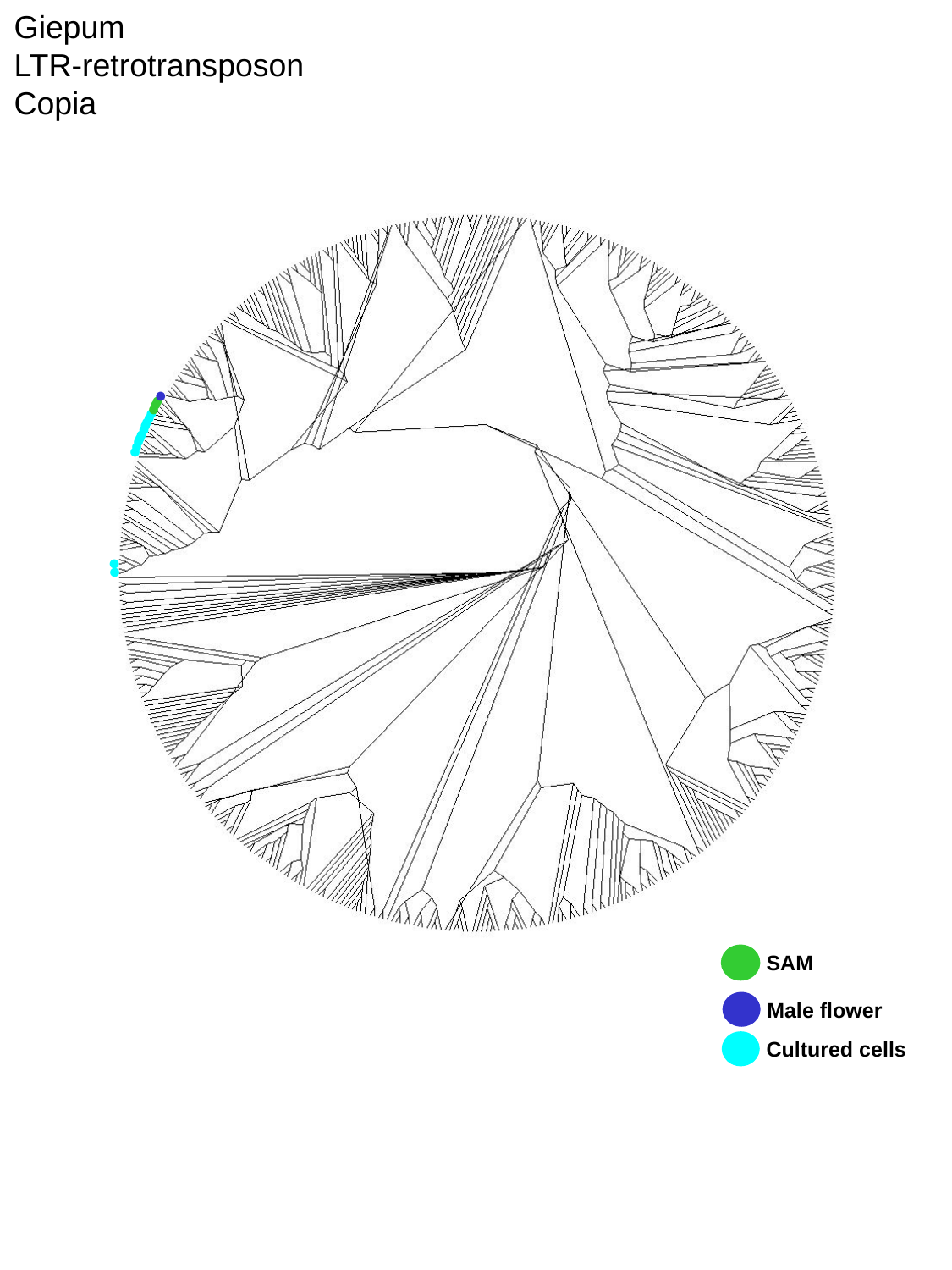

Giepum
LTR-retrotransposon
Copia
●
●
●
●
●
●
●
●
●
●
●
●
●
●
●
●
SAM
7
Male flower
Cultured cells

## Slide 4
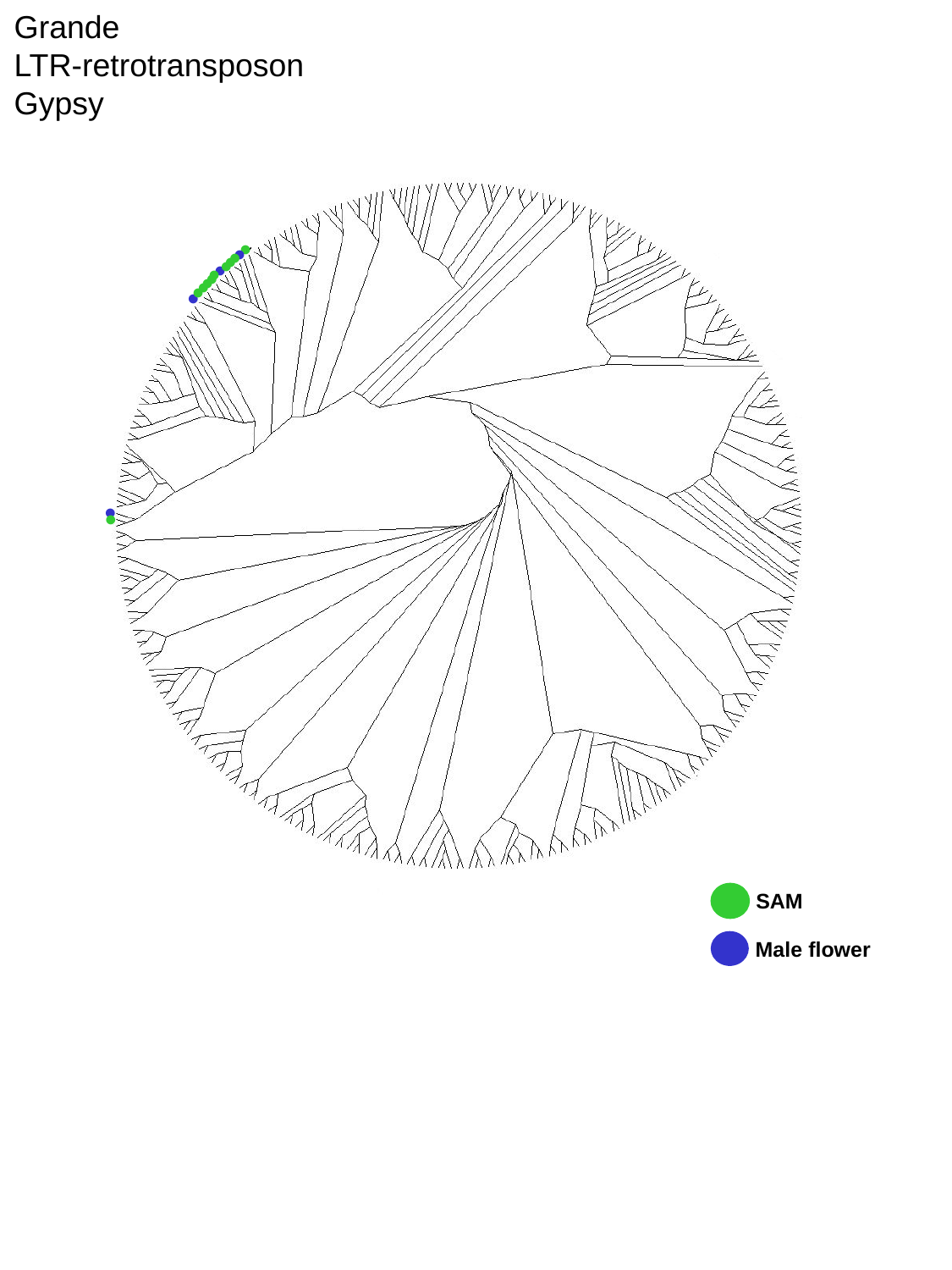

Grande
LTR-retrotransposon
Gypsy
●
●
●
●
●
●
●
●
●
●
●
●
●
●
SAM
7
Male flower

## Slide 5
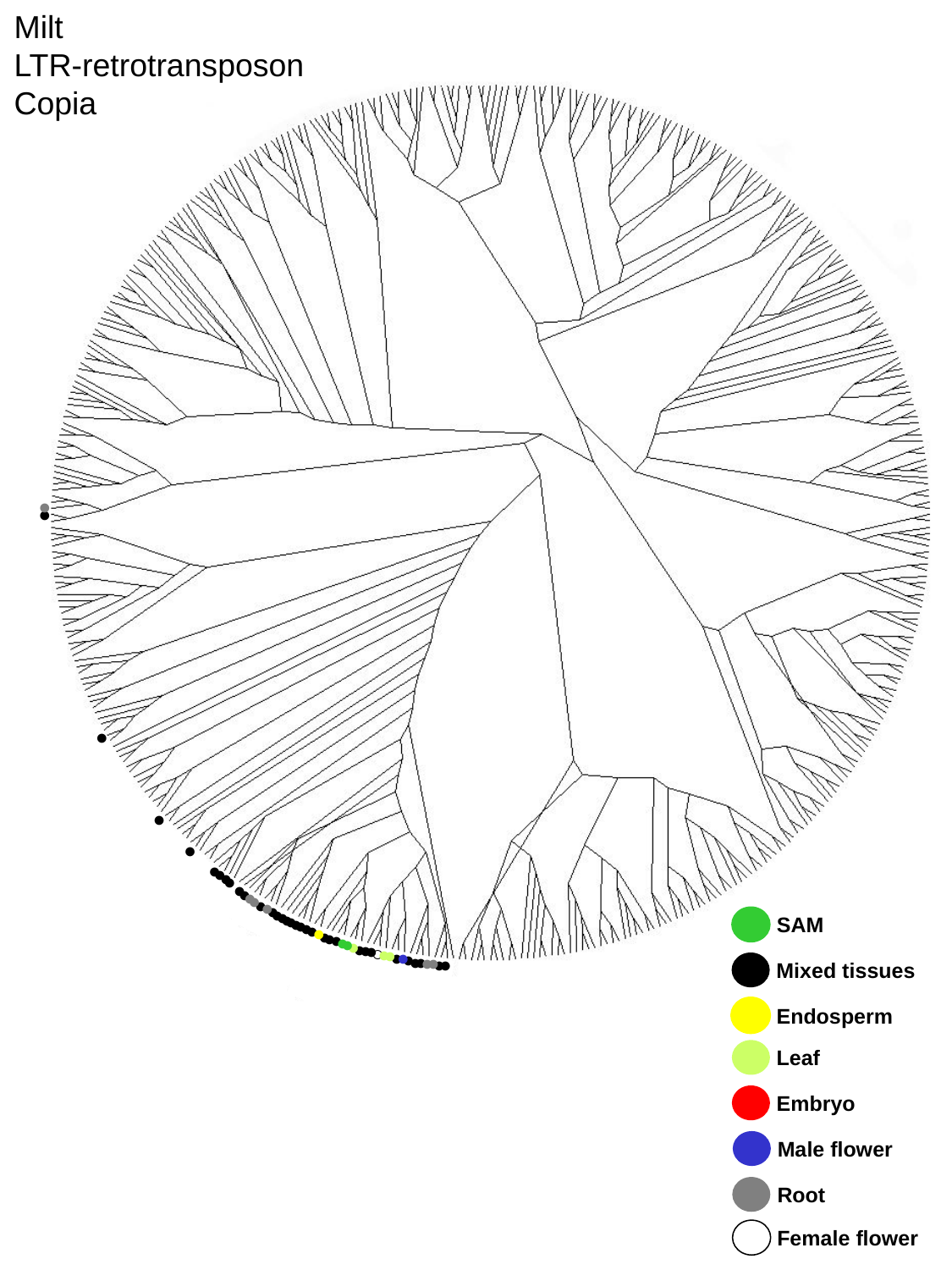

Milt
LTR-retrotransposon
Copia
●
●
●
●
●
●
●
●
●
●
●
●
●
●
●
●
●
●
●
●
●
SAM
●
7
●
●
●
●
●
●
●
●
●
●
●
●
○
●
●
●
●
●
●
●
●
●
●
●
Mixed tissues
5
Endosperm
Leaf
Embryo
Male flower
Root
Female flower

## Slide 6
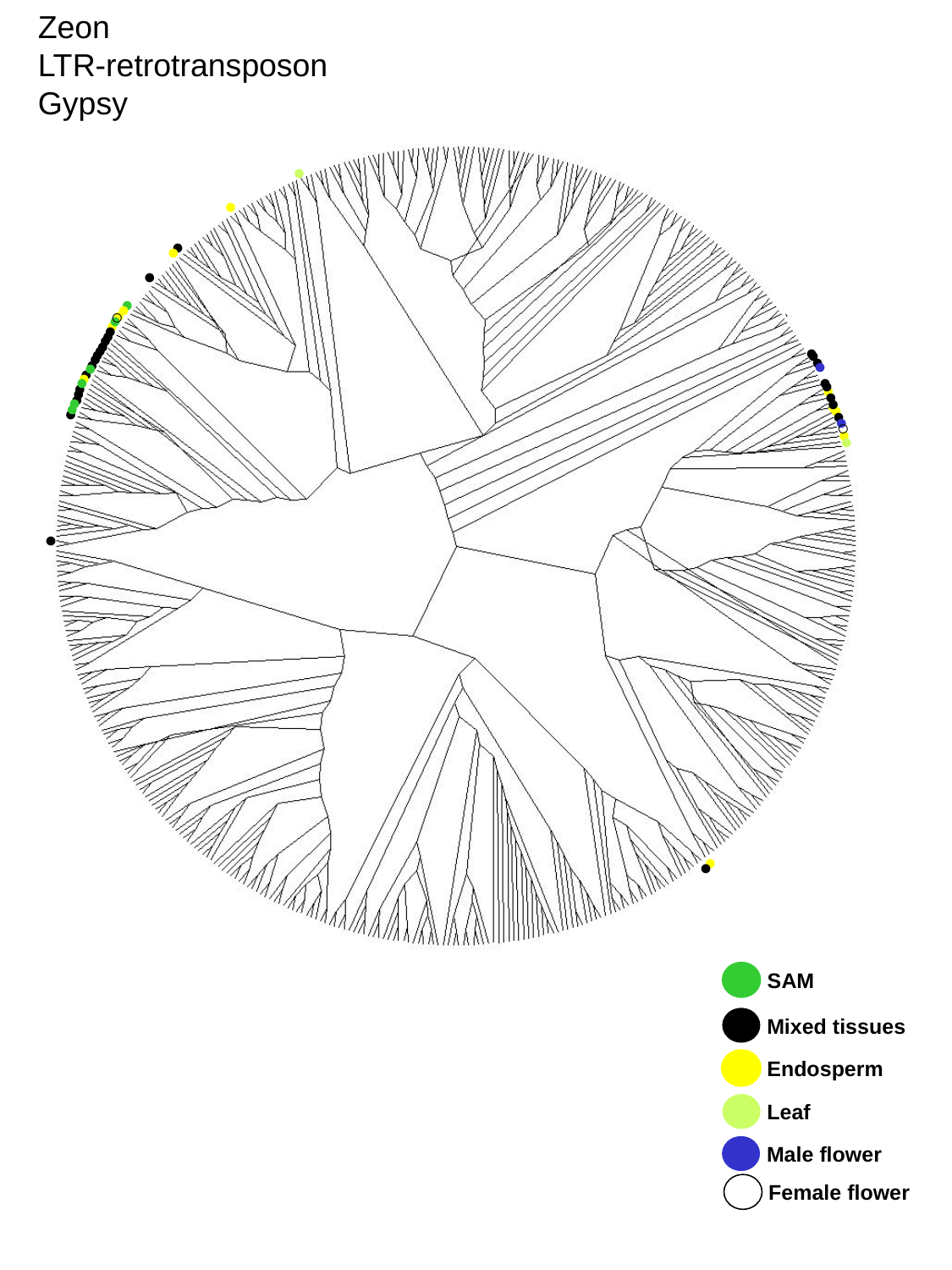

Zeon
LTR-retrotransposon
Gypsy
●
●
●
●
●
●
●
●
○
●
●
●
●
●
●
●
●
●
●
●
●
●
●
●
●
●
●
●
●
●
●
●
●
●
●
●
●
●
●
●
●
●
○
●
●
●
●
●
SAM
7
Mixed tissues
5
Endosperm
Leaf
Male flower
Female flower
